# (ReMoV)X_2_ (X = S, Se) Ternary Alloy Nanosheets for Enhanced Electrocatalytic Hydrogen Evolution Reaction

**DOI:** 10.1002/smll.202503399

**Published:** 2025-04-24

**Authors:** Junaid Ihsan, Ju Yeon Kim, In Hye Kwak, Irtiqa Mishal, Jun Hyeok Choi, Jung Eun Ahn, Sang‐Gil Lee, Seung Jo Yoo, Ik Seon Kwon, Jeunghee Park, Hong Seok Kang

**Affiliations:** ^1^ Department of Advanced Materials Chemistry Korea University Sejong 339‐700 Republic of Korea; ^2^ Research Center for Materials Analysis Korea Basic Science Institute Daejeon 305‐806 Republic of Korea; ^3^ Department of Energy Science & Engineering Kunsan National University 558 Daehak‐ro Gunsan Jeonbuk 54150 Republic of Korea; ^4^ Department of Nano and Advanced Materials Jeonju University Chonju Chonbuk 55069 Republic of Korea

**Keywords:** first‐principles calculations, hydrogen evolution reaction, metallic nanosheets, ReMoV ternary alloys, transition metal dichalcogenide

## Abstract

Modulating the electronic structure of 2D transition metal dichalcogenides via alloying can extend their potential applications. In this study, composition‐tuned ternary alloy nanosheets of (ReMoV)X_2_ (X = S and Se) are synthesized using solvothermal and colloidal reactions, respectively. Ternary alloying occurred with homogeneous atomic mixing over a wide range of compositions (*x*_V_ = 0.16–0.80). Compared to (ReV)X_2_ binary alloying, ternary alloying produces a more metallic phase with less oxidation. Increasing *x*_V_ induces a phase change into a more metallic 1T phase. The (ReMoV)S_2_ nanosheets demonstrate enhanced electrocatalytic activity toward the acidic hydrogen evolution reaction (HER) compared to (ReV)S_2_. Density functional theory calculations predict that ternary alloying increases the metallicity of the nanosheets. In addition, the Gibbs free energy calculation for hydrogen adsorption (ΔG_H*_) shows that ternary alloying effectively activates the basal S atoms for the HER, supporting the enhanced catalytic performance observed experimentally.

## Graphical abstract

Composition‐tuned (ReMoV)X_2_ (X = S, Se) ternary alloy nanosheets are synthesized using solution reactions. A homogeneous atomic mixing occurred in the metallic 1T phase. The electrocatalytic activity toward the acidic hydrogen evolution reaction is enhanced for the sulfide nanosheets. Density functional theory calculations supported the experimental data.

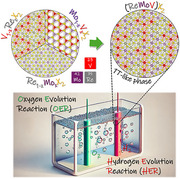

## 1 Introduction

The rapid growth of fossil fuel consumption has caused significant energy crisis and environmental pollution, necessitating the development of clean and renewable energy sources. Among alternative energy resources, H_2_ produces only water through its combustion and has the highest mass‐energy density (142 MJ kg^−1^). The generation of H_2_ via electrocatalytic water splitting (2H_2_O → 2H_2_ + O_2_) is probably the most environmentally friendly technique, and therefore, a tremendous amount of research is being conducted all over the world. An important issue is developing efficient and cheap electrocatalysts for the cathodic hydrogen evolution reaction (HER) because the most efficient HER electrocatalysts so far require Pt, an expensive noble metal.

Transition metal dichalcogenides (TMDs) have recently attracted attention for catalytic HER.^[^ ^1^, ^2^, ^3^, ^4^, ^5^, ^6^ ^]^ TMDs are typical 2D layered materials with the chemical formula MX_2_, where M is a group V–VII transition metal and X = S or Se. Each layer in TMD comprises a sublayer of M atoms sandwiched between two sublayers of X atoms. Pristine TMDs have intrinsically low electrical conductivity, and their exposed X sublayers are not often very catalytically active.^[^ ^1^, ^2^, ^3^ ^]^ These drawbacks are usually resolved by forming chalcogen vacancies, substitutional/adatom doping, metal/molecule ion intercalation, or alloying.^[^ ^4^, ^5^, ^6^ ^]^ Enhanced electrocatalytic activity toward HER or CO_2_ reduction has been demonstrated for a number of binary metal‐site alloy systems such as MoW,^[^ ^7^, ^8^ ^]^ MoRe,^[^ ^9^, ^10^, ^11^ ^]^ NbTa_,_^[^ ^12^ ^]^ MoNb,^[^ ^13^ ^]^ MoV,^[^ ^14^ ^]^ NbV,^[^ ^15^ ^]^ WV,^[^ ^16^ ^]^ WNb,^[^ ^17^ ^]^ WRe.^[^ ^18^ ^]^ and ReV.^[^ ^19^, ^20^ ^]^ Notably, ternary alloy nanosheets like (MoNbV)Se_2_ and (MoWV)Se_2_ via colloidal reaction have demonstrated a synergistic effect to improve the HER performance of constitutional binary alloys.^[^ ^21^, ^22^ ^]^ The use of a large variety of components allows researchers to optimize the electronic structure of TMD alloys for the electrocatalytic reaction.

Semiconducting ReX_2_ nanosheets exhibit excellent (photo)electrocatalytic performance.^[^ ^23^, ^24^, ^25^, ^26^, ^27^, ^28^, ^29^, ^30^, ^31^, ^32^, ^33^, ^34^, ^35^, ^36^, ^37^, ^38^, ^39^, ^40^, ^41^, ^42^, ^43^, ^44^, ^45^, ^46^, ^47^ ^]^ In a pioneering work, Yang et al. reported enhanced HER performance of (ReMo)S_2_ alloy monolayers at *x*_Mo_ (mole fraction of Mo) = 0.45 synthesized via chemical vapor deposition.^[^ ^9^ ^]^ Our research group synthesized (ReMo)S_2_ and (ReMo)Se_2_ nanosheets by hydrothermal reactions, and the best HER performance occurred at *x*_Mo_ = 0.5 and 0.1, respectively, because of the more metallic phase than the ReX_2_.^[^ ^10^, ^11^ ^]^ Lee et al. reported the colloidal synthesis of (WRe)S_2_ monolayers quantum dots with enhanced catalytic HER at *x*_Re_ = 0.49.^[^ ^18^ ^]^ Alloying with metallic VX_2_ can also improve the catalytic activity of ReX_2_. We reported the synthesis of (ReV)Se_2_ and (ReV)S_2_ nanosheets via colloidal and solvothermal reactions, respectively, showing an enhanced HER performance at *x*_V_ = 0.2–0.8.^[^ ^19^, ^20^ ^]^ By introducing one more component, (ReMoV)X_2_ nanosheets are expected to exhibit even better catalytic activity, but the related results have not been reported.

In this study, (ReMoV)S_2_ and (ReMoV)Se_2_ ternary alloy nanosheets were synthesized using solvothermal and colloidal reactions, respectively. Their composition was successfully tuned over a wide range (*x*_Re_ = 0.10 – 0.5, *x*_Mo_ = 0.10 – 0.5, and *x*_V_ = 0.16–0.8). Atomic‐resolution scanning transmission electron microscopy (STEM) was employed to examine the crystal structure with the atomic distributions of metals. We analyzed thoroughly their electronic structures using X‐ray photoelectron spectroscopy (XPS) and extended X‐ray absorption fine structure (EXAFS). The electrocatalytic activity toward the HER in acidic electrolytes was studied for both the sulfide and selenide alloys, in comparison with the (ReV)X_2_ binary alloys. We also monitored the electronic structures during the HER using ex situ EXAFS. First‐principles calculations were performed to predict the crystal structures, density of states, and Gibbs free energy (ΔG_H*_) of the ternary alloy nanosheets. The calculation results support the observed crystal/electronic structures as well as the HER performance of ternary alloys.

## 2 Results and Discussion

**Scheme 1:**
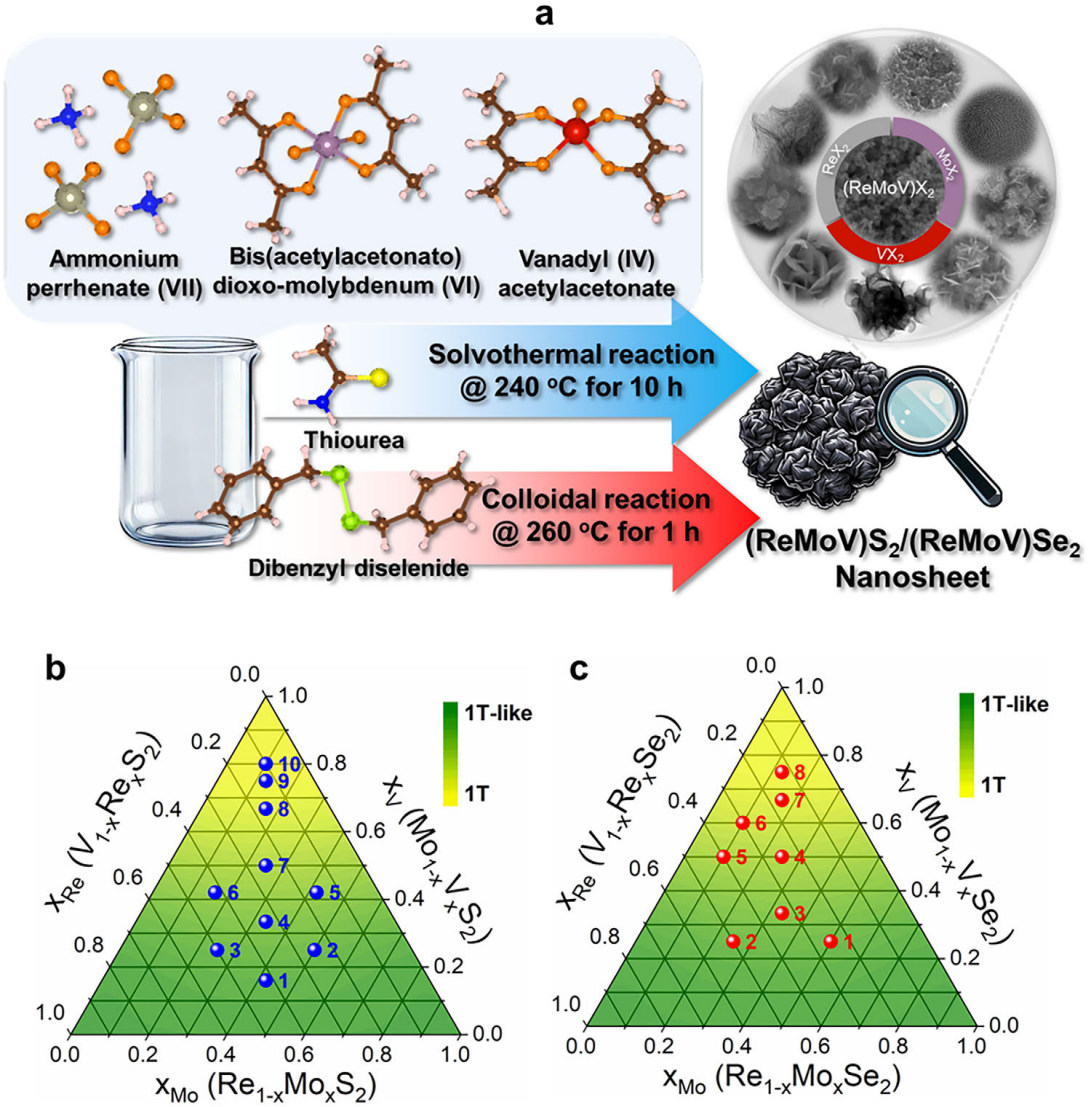
a) Schematic diagram for the synthesis of (ReMoV)S_2_ and (ReMoV)Se_2_ alloy nanosheets, respectively, using solvothermal and colloidal reactions. Ternary plot for the composition (*x*_Re_, *x*_Mo_, *x*_V_) of b) (ReMoV)S_2_ and c) (ReMoV)Se_2_ alloy nanosheets synthesized in the present work. For (ReMoV)S_2_, **1**S: (0.42, 0.42, 0.16), **2**S: (0.25, 0.5, 0.25), **3**S: (0.50, 0.25, 0.25), **4**S: (0.33, 0.33, 0.33), **5**S: (0.16, 0.42, 0.42), **6**S: (0.42, 0.16, 0.42), **7**S: (0.25, 0.25, 0.5), **8**S: (0.167, 0.167, 0.67), **9**S: (0.125, 0.125, 0.75), and **10**S: (0.10, 0.10, 0.80). For (ReMoV)Se_2_, **1**Se: (0.25, 0.5, 0.25), **2**Se: (0.50, 0.25, 0.25), **3**Se: (0.33, 0.33, 0.33), **4**Se: (0.25, 0.25, 0.5), **5**Se: (0.4, 0.1, 0.5), **6**Se: (0.3, 0.1, 0.6), **7**Se: (0.167, 0.167, 0.67), and **8**Se: (0.125,0.125, 0.75).

As depicted in **Scheme** **1**a, (ReMoV)S_2_ and (ReMoV)Se_2_ alloy nanosheets were synthesized using solvothermal and colloidal reactions, respectively. The precursors were ammonium perrhenate (NH_4_ReO_4_), bis(acetylacetonato)dioxo molybdenum (VI) (MoO_2_(C_5_H_7_O_2_)_2_), and vanadyl acetylacetonate (VO(C_5_H_7_O_2_)_2_). The procedures are the same as those of (ReV)X_2_, except for adding the Mo precursor.^[^ ^19^, ^20^ ^]^ Starting from the three unitary compositions ReX_2_, MoX_2_, and VX_2_, we prepared 10 samples of sulfide alloys with *x*_Re_ = 0.10–0.5, *x*_Mo_ = 0.10–0.5, and *x*_V_ = 0.16–0.8, as shown in the ternary diagram of Scheme 1b. For comparison, thermally annealed samples were also prepared by heating the as‐grown samples at 400 °C. Eight selenide alloys were also synthesized with *x*_Re_ = 0.10–0.5, *x*_Mo_ = 0.10–0.5, and *x*_V_ = 0.25–0.75, as shown in the ternary diagram of Scheme 1c. In the rest of the paper, the sulfide and selenide samples are sometimes referred to as “**a**S” and “**b**Se” (**a** = 1–10, **b** = 1–8), respectively. The ternary compositions may also be denoted as (ReMo)_1‐_ *_x_* V*_x_* X_2_ to highlight the V composition (*x*_V_).

**Figure 1:**
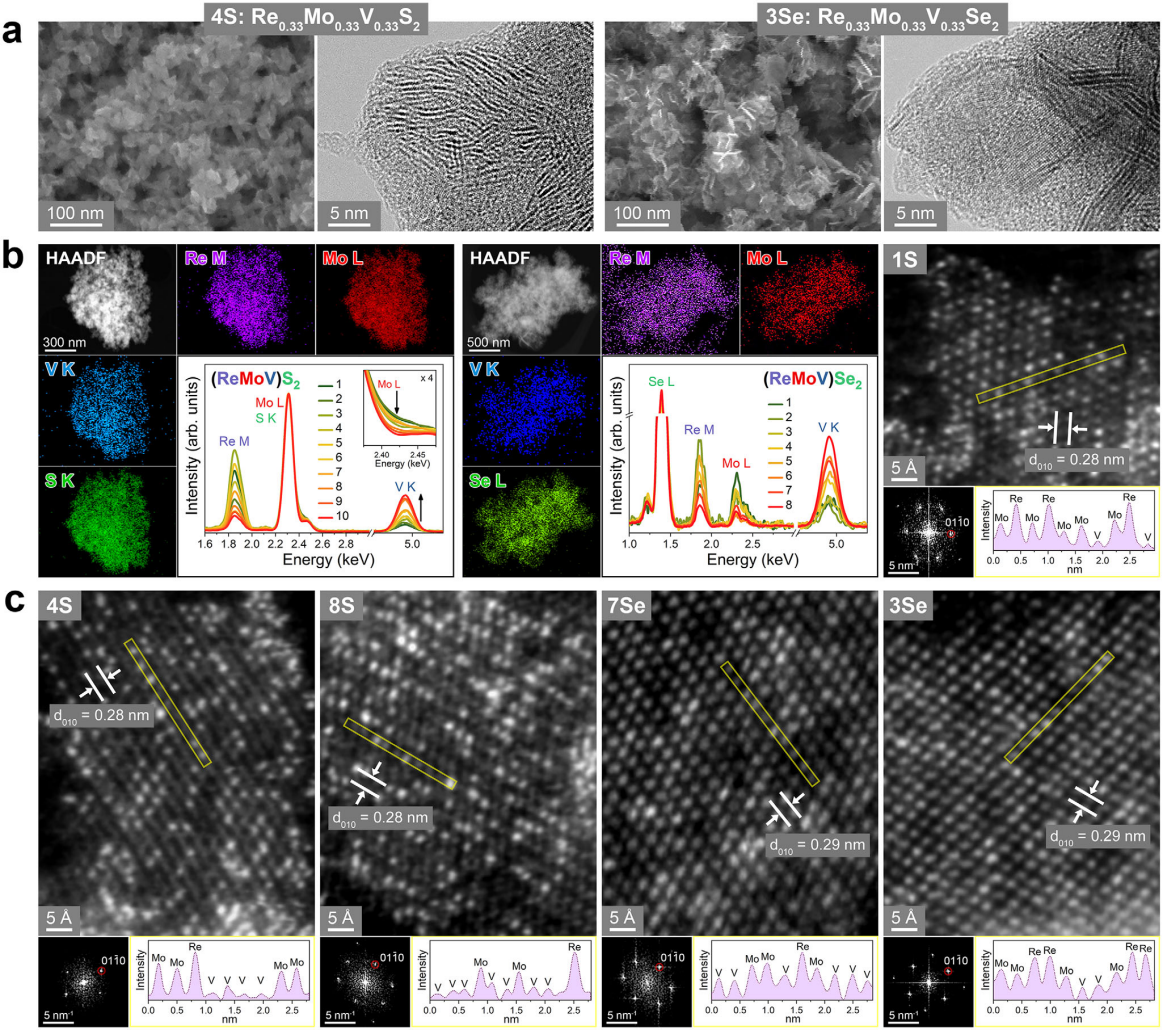
a) SEM and HRTEM images of Re_0.33_Mo_0.33_V_0.33_S_2_ (sulfide sample **4**S) and Re_0.33_Mo_0.33_V_0.33_Se_2_ (selenide sample **3**Se). b) HAADF STEM image and EDX elemental mapping of Re (M shell), Mo (L shell), V (K shell), and S (K shell) or Se (L shell) for **4**S and **3**Se. Insets: EDX spectra showing composition tuning in the 10 samples of (ReMoV)S_2_ and 8 samples of (ReMoV)Se_2_. For sulfides, the inset corresponds to the enlarged edge part of overlapped Mo L/S K shell, showing the composition tuning of Mo. c) Atomic‐resolution HAADF STEM images and corresponding FFT images for **1**S (*x*_V_ = 0.16), **4**S (*x*_V_ = 0.33), **8**S (*x*_V_ = 0.67), **3**Se (*x*_V_ = 0.33), and **7**Se (*x*_V_ = 0.67). The [01$\bar{1}$0] spots (FFT) matched to *d*_010_ = 0.28 nm for sulfide and 0.29 nm for selenide. Darker regions of VS_2_ become dominant as *x*_V_ increases. Line profile along the marked area, showing the random distribution of Re, Mo, and V atoms that were identified by comparing the intensity of adjacent atoms.

**Figure** **1**a shows the scanning electron microscopy (SEM) and high‐resolution transmission electron microscopy (HRTEM) images for **4**S and **3**Se (*x*_Re_ = *x*_Mo_ = *x*_V_ = 0.33). The layered nanosheets aggregated into flower‐like nanoparticles (nanoflowers). Figures S1 and S2 (Supporting Information) show the SEM and HRTEM images of other sulfide and selenide samples, respectively. The ReX_2_ nanosheets comprised 2–5 layers (thickness: ≈2 nm) that aggregated into nanoflowers with a size of ≈100 nm. By contrast, the thickness and size of the VX_2_ nanosheets were ≈10 and 200 nm, respectively. The MoS_2_ nanosheets (thickness: 2–5 nm) aggregated to form random‐sized nanoflowers (30–50 nm), whereas the MoSe_2_ nanosheets had a thickness of ≈2 nm and were bundled into nanoflowers ≈100 nm in size. After alloying, the size of the nanoflowers decreased to less than 50 nm. The interlayer distance (*d*_001_) of sulfide nanosheets was in a wider range and larger (≈10 Å) than that of selenide nanosheets (≈6 Å).

Energy‐dispersive X‐ray spectroscopy (EDX) data verified the successful composition tuning and homogeneous distribution of Re, Mo, V, and S (or Se) (see Figures S1 and S2, Supporting Information). EDX elemental mapping and the corresponding high‐angle annular dark‐field (HAADF) STEM images of **4**S and **3**Se are shown in Figure 1b. In the normalized EDX spectra, the relative intensities of Re, Mo, and V peaks changed throughout the series. The composition of each sample matched that of the precursor (Table S1, Supporting Information). Seven of the sulfide samples (**1**S–**7**S) have zero S vacancy (defined as C_VX_ = 1‐ $\frac{1}{2}\frac{\left[X\right]}{\left[Re]+[Mo]+[V\right]}$, where X = S or Se), and **8**S–**10**S have 3%–6% (see Table S1, Supporting Information). The ternary alloys in either the as‐grown or annealed conditions had smaller C_VS_ than that of Re_1‐_ *_x_* V*_x_* S_2_.^[^ ^20^ ^]^ By contrast, the selenide samples contained 4%–8% Se vacancies, which was comparable to that of Re_1‐_ *_x_* V*_x_* Se_2_.^[^ ^19^ ^]^ These results consistently showed that the chalcogen vacancies increased with increasing *x*_V_. We have previously proposed a model for this, in which the slower reaction of VX_2_ produced X vacancies by forming V─O bonds.^[^ ^19^, ^20^ ^]^

Figure 1c shows the atomic‐resolution HAADF STEM images of four sulfide samples **1**S (*x*_Re_ = 0.42, *x*_Mo_ = 0.42, *x*_V_ = 0.16), **4**S (0.33, 0.33, 0.33), **8**S (0.167, 0.167, 0.67) and two selenide samples **3**Se (0.33, 0.33, 0.33) and **7**Se (0.167, 0.167, 0.67). The S atoms (Z = 16) were not clearly identified because of the brighter Re (Z = 75), Mo (Z = 41), and V (Z = 23) atoms. The darker regions corresponding to the VX_2_ sites become dominant at higher *x*_V_. The 1T structure is obviously the major one in both the sulfide and selenide alloys. The corresponding fast Fourier transform (FFT) image shows the [01$\bar{1}$0] reflection spots of the 1T‐like phase. The FFT image provides *d*_010_ = 2.8 Å for sulfide and 2.9 Å for selenide, corresponding to the lattice constant *a* = 3.24 and 3.35 Å, respectively, which are close to the reference value of VS_2_ and VSe_2_, as discussed later. The line profiles along the marked area support the homogeneous mixing of Re, Mo, and V atoms, and the increased number of V sites with increasing *x*_V_.

**Figure 2:**
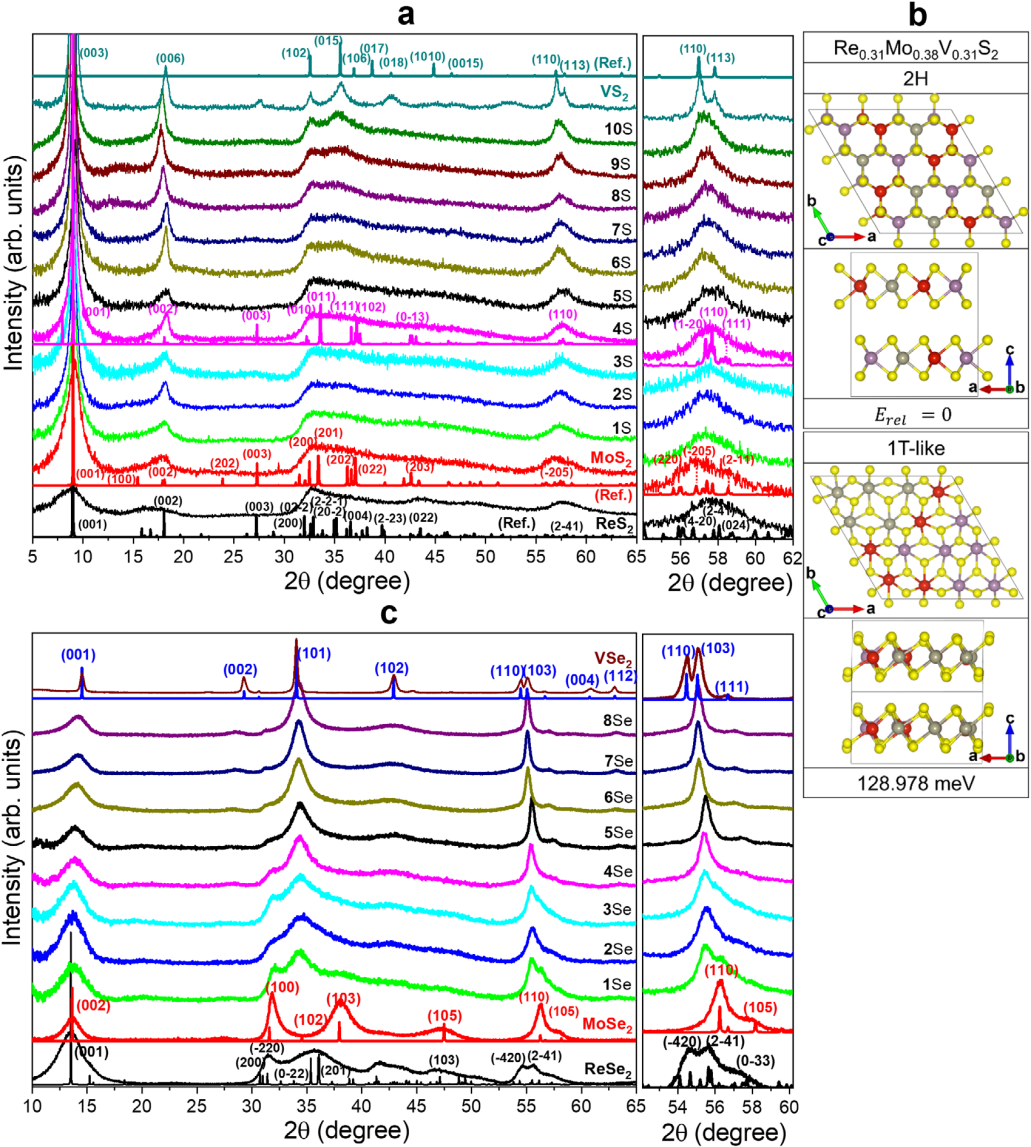
a) Left: full‐range XRD patterns of ReS_2_, MoS_2_, VS_2_, and (ReMoV)S_2_ samples, together with the reference peaks generated using the calculated lattice constants of 1T′′ ReS_2_, 1T′ MoS_2_, 3R‐1T VS_2_, and Re_0.31_Mo_0.38_V_0.31_S_2_ (for sample **4**S). Right: magnified XRD peak at 2θ = 54°–62° for (2 $\bar{4}$ 1)_1T′′_ of ReS_2_, ($\bar{2}$ 05)_1T′_ of MoS_2_, (110)_3R‐1T_/(113)_3R‐1T_ of VS_2_, and (110)_1T_ of **4**S. b) Crystal structures (ball‐and‐stick models) of the two‐layered (4 × 4 × 1) supercell and relative energy (E_rel_) for 2H and 1T‐like phase of Re_0.31_Mo_0.38_V_0.31_S_2_, projected along the basal plane (*c* axis, top) and *b*‐axis (bottom). Gray, violet, red, and yellow balls represent Re, Mo, V, and S atoms, respectively. c) Left: full‐range XRD patterns of ReSe_2_, MoSe_2_, VSe_2_, and (ReMoV)Se_2_ samples, together with the reference peaks calculated using the lattice constants of 1T′′ ReSe_2_, 2H MoSe_2_, and 1T VSe_2_. Right: magnified XRD peak at 2θ = 52°–60°.

The crystal phases of the ternary alloys were examined using XRD. **Figure** **2**a shows the data for ReS_2_, MoS_2_, VS_2_, and their ternary alloys. All samples showed a peak at 2θ = 9°, indicating that *d*_L_ was ≈10 Å. The lattice constants of ReX_2_, MoX_2_, and VS_2_ were determined using spin‐polarized density functional theory (DFT) calculations, as listed in Table S2 (Supporting Information). The data of ReS_2_ matched those of 1T″ phase, with (*a*, *b*, *c*) = (6.49, 6.39, 10.35) Å and (α, β, γ) = (105.10°, 91.82°, 118.82°). Except for the *c* value, all others are similar to those of JCPDS No. 24–0922; (*a*, *b*, *c*) = (6.455, 6.362, 6.401) Å and (α, β, γ) = (154.04°, 91.60°, 118.97°). For MoS_2_, the XRD peaks were matched to those of the 1T′ phase with *c* = 9.8 Å, based on the calculated lattice constants of (*a*, *b*, *c*) = (6.56, 3.19, 5.88) Å and γ = 119°. The broad peak feature of ReS_2_ and MoS_2_ was similar. In the case of VS_2_, the peaks matched the 3R‐stacking 1T (referred to as 3R‐1T) phase with *a* = 3.23 Å and *c* = 29.2 Å (*d*_L_ = 9.7 Å).^[^ ^20^ ^]^ After heating the as‐grown samples at 400 °C, *d*_L_ returned to 6 Å (see Figure S3, Supporting Information). The 1T′ phase MoS_2_ and 3R‐1T phase VS_2_ transformed into 2H MoS_2_ and V_3_S_4_/V_5_S_8_ phases, respectively. The expansion of *d*_L_ was already discussed in the work of (ReV)S_2_, using the intercalation of molecular ions (e.g., hydrated NH_4_^+^ with an ionic radius of 3.31 Å).^[^ ^20^ ^]^ Spin‐polarizedDFT calculations were also performed to show that intercalation of NH_3_ or NH_4_^+^ can occur over the entire composition range to expand the interlayer distance, and it also stabilizes the 3R‐1T phase VS_2_.^[^ ^20^ ^]^

Samples **6**S–**10**S show the peaks of 3R‐1T VS_2_, *e.g*., (006)_3R‐1T_ at 2θ = 18.2°, (102)_3R‐1T_ at 2θ = 32.5°, and (015)_3R‐1T_ at 2θ = 35.4°. **10**S exhibits a peak pattern closest to that of VS_2_. These XRD patterns suggest a phase transition to 3R‐1T VS_2_ at *x*_V_ = 0.42 (**6**S), similar to the case of Re_1‐_ *_x_* V*_x_* S_2_ (*x* = 0.5).^[^ ^20^ ^]^ The right panel shows the magnified XRD peak at 2θ = 54°–62° for (2 $\bar{4}$ 1)_1T′′_ of ReS_2_, ($\bar{2}$05)_1T′_ of MoS_2_, and (110)_3R‐1T_ and (113)_3R‐1T_ of VS_2_. The ReS_2_, MoS_2_, and VS_2_ samples have similar lattice constant for the basal planes (*a* and *b*): *a*_1T′′_/2 (= 3.25 Å) and *b*_1T′′_/2 (= 3.20 Å) of ReS_2_, *a*_1T′_/2 (= 3.28 Å) and *b*_1T′_ (= 3.17 Å) of MoS_2_, and *a*_3R_ (= 3.23 Å) of VS_2_. The negligible peak shift (Δ2θ = 0.4°) of alloy samples **1**S–**10**S is due to the similar lattice constants of these three unitary phases.

We performed spin‐polarized DFT calculations for the Re_0.31_Mo_0.38_V_0.31_S_2_ composition as a model for **4**S (Re:Mo:V = 1:1:1) to understand the crystal phases of the alloys. The (4 × 4 × 1) supercell monolayers (48 atoms) were stacked infinitely; however, interlayer intercalation was not considered because of the complexity. For the V atoms, the effective Hubbard U parameter (U_eff_) was set to 1 eV to correlate the localized *d* electrons. In our previous work, we showed that U_eff_ = 1 eV led to a better agreement with our experimental lattice constant of VSe_2_ than U_eff_ = 3 eV.^[^ ^20^ ^]^ Both 2H‐ and 1T‐like phases were considered for various configurations, as shown in Figure S4 (Supporting Information). The lattice parameters and the total and relative energies are summarized in Table S2 (Supporting Information).

Figure 2b shows the most stable configurations of the 2H and 1T‐like phases. They comprise randomly dispersed Re, Mo, and V atoms, which are consistent with the STEM images. The lattice constants of the 1T‐like structure are (*a*, *b*, *c*) = (3.23, 3.24, 5.93) Å and (*α*, *β*, *γ*) = (89.72°, 90.66°, 120.18°). Because *a* ≈ *b*, *α* ≈ *β ≈* 90°, and *γ ≈* 120°, this structure is closer to that of the 1T phase than that of 1T″ or 1T′. In the 1T″ and 1T′ phases, the metal−metal distance alternates along the *a*/*b*‐axes and *a*‐axis, respectively. In the ternary alloy, there exist only locally asymmetric metal–metal distances with almost no bond alternation, and thus the metal–metal distance was closer to that of the 1T phase (so we referred to it as 1T‐like). The 2H structure (*a* = 3.14 Å and *c* = 12.35 Å) is more stable than the 1Τ‐like structure by 128.978 meV per atom. We plotted the calculated XRD pattern using the lattice constants of the 1T‐like structure with an expanded *c* = 9.8 Å (see Figure 2a). The experimental XRD peaks of **4**S agree well with the calculated ones, indicating that the ternary samples prefer the 1T‐like structure, which is also consistent with the STEM images.

The XRD patterns of the selenide samples are shown in Figure 2c. The peaks of ReSe_2_ matched the 1T″ phase ReSe_2_ with (*a*, *b*, *c*) = (6.716 Å, 6.602 Å, 6.90 Å) and (α, β, γ) = (104.15°, 91.82°, 118.15°), close to the calculated ones. These values are close to those of the reference (JCPDS No. 74–0611, P̄1, *a* = 6.716 Å, *b* = 6.602 Å, *c* = 6.728 Å, α = 104.90°, β = 91.820°, γ = 118.94°). MoSe_2_ was in the 2H phase with (*a*, *c*) = (3.287 Å, 12.925 Å), corresponding to JCPDS No. 29–0914 (P6_3_/mmc). The peaks of 1T phase VSe_2_ matched to the reference (JCPDS No. 89–1641; P3_m1) with (*a*, *c*) = (3.3587 Å, 6.1075 Å). The *d*_L_ value of ReSe_2_ and MoSe_2_ is 6.5 Å, while that of VSe_2_ is 6.1 Å. As the sample number increases, the (001) peak shifts gradually from 2θ = 13.7° to 14.2°, indicating a decrease of *d*_L_ due to the phase transition to 1T VSe_2_.

The right panel displays the magnified patterns at 2θ = 52°–62°, showing that the peak becomes sharper after **4**Se (*x*_V_ = 0.5). This peak shifts from 2θ = 55.5° to 55.1° with increasing *x*_V_. The expansion of the basal lattice constants is due to the phase transition to 1T VSe_2_. Therefore, the phase transition to 1T VSe_2_ probably occurs at *x*_V_ = 0.5 (**4**Se), similar to the 1T″ → 1T phase transition of Re_1‐_ *_x_* V*_x_* Se_2_ that occurs at *x* = 0.5.^[^ ^19^ ^]^ All alloy samples show the 1T phase peak at 2θ = 34°, which cannot be assigned to either 1T′′ ReSe_2_ or 2H MoSe_2_. Similar to the sulfide series, the selenide series probably has a 1T‐like structure, which is supported by the STEM images. Therefore, as *x*_V_ increases, both the sulfide and selenide alloys undergo a transition from the 1T‐like to 1T VX_2_ phase. The favorable formation of the 1T‐like phase in the experiment is probably related to the kinetically controlled solution reaction.

**Figure 3:**
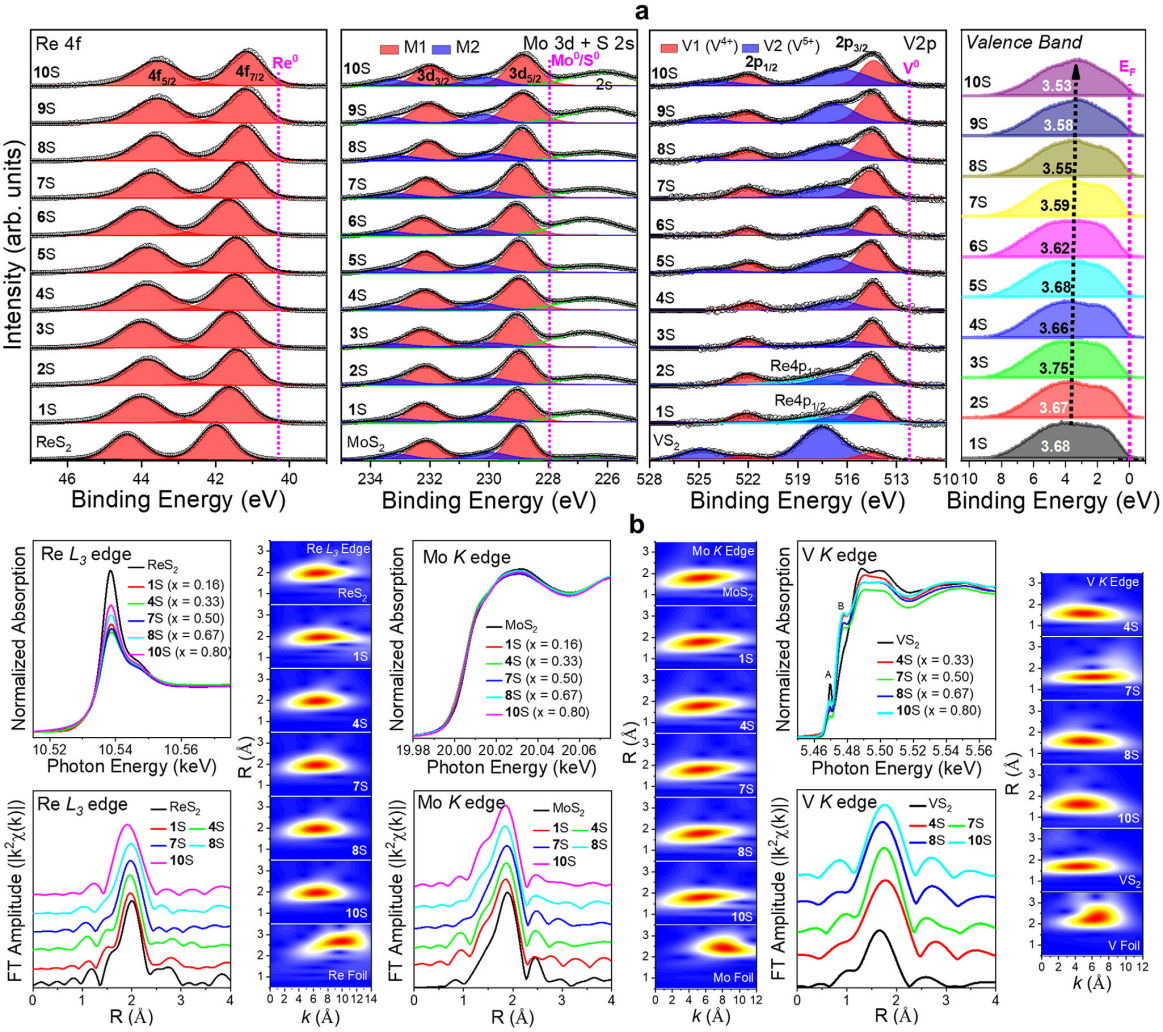
a) Fine‐scan XPS peaks of Re 4*f* (4*f*_7/2_ and 4*f*_5/2_ separated by 2.43 eV), Mo 3*d* (3*d*_5/2_ and 3*d*_3/2_ separated by 3.13 eV), V 2*p* (2*p*_3/2_ and 2*p*_1/2_ separated by 7.64 eV), and VBS for ReS_2_, MoS_2_, VS_2_, and sulfide samples (**1**S–**10**S). Positions of neutral Re^0^ (4*f*_7/2_ at 40.3 eV), Mo^0^ (3*d*_5/2_ at 228.0 eV), and V^0^ (2*p*_3/2_ at 512.2 eV) are marked by dotted vertical lines. The experimental data (open circles) are fitted by a Voigt function after a Shirley‐type baseline correction. The sum of the resolved bands is represented by a black line. In VBS, the Fermi level (E_F_) is marked by a dotted vertical line. The *d*‐band center (ɛ_d_) value is also labeled with a dotted line arrow to emphasize the redshift with increasing *x*_V_. b) XANES and (non‐phase‐corrected) *k* ^2^‐weighted FT EXAFS above the Re L_3_‐edge (10.53 keV), Mo K‐edge (20.01 keV), and V K‐edge (5.46 keV), and their corresponding WT EXAFS (*k*‐space vs *R*‐space) for sulfide samples **1**S, **4**S, **7**S, **8**S, and **10**S.

The whole XPS results are shown in Figure S5 (Supporting Information). **Figure** **3**a presents the Re 4*f*, Mo 3*d*, and V 2*p* spectra of the (ReMoV)S_2_ alloys and the corresponding unitary samples. The Re 4*f* peaks correspond to Re‐S bonding structures. The alloys exhibited a more redshifted peak than ReS_2_, probably owing to the higher metallicity of the 1T‐like phase than the semiconducting 1T″ phase ReS_2_. As the sample number increases, the Re peaks redshift continuously, indicating a more metallic nature at higher V compositions. The redshift (41.1 eV at *x*_V_ = 0.8) is more significant than that (41.4 eV at *x*_V_ = 0.8) of binary counterparts, indicating more metallic properties than the binary alloys.^[^ ^20^ ^]^ The Mo 3*d* peaks were resolved into the M1 and M2 bands that correspond to Mo─S bonds and defects, respectively. The S 1*s* peak appears at 226.5 eV, which is redshifted from that of S^0^ at 228.0 eV. As *x*_V_ increases, the M1 band of Mo 3*d*_5/2_ redshifts from 229.0 eV (metallic 1T′ phase MoS_2_ and **1**S) to 228.8 eV (**10**S), indicating the increased metallicity at higher *x*_V_. The V 2*p* peaks were resolved into the V1 (V─S bond with V^4+^) and V2 (V─O bond with V^5+^) bands; V1 (at 514 eV) and V2 (at 517–518 eV) bands for the V 2*p*_3/2_ peak. In samples **1**S and **2**S, the band at 519 eV is ascribed to Re 4*p*_1/2_ because of the higher Re compositions. As the sample number increases, the fraction of the V2 band increases. For VS_2_, the V1 band almost disappears. We calculated the oxidation number of V atoms using the ratio of V1 and V2 bands and found that it increases from 4.3 (**1**S) to 4.5 (**10**S). In the case of Re_1‐_ *_x_* V*_x_* S_2_, this value is 4.5–4.7. Therefore, sample oxidation is reduced in the ternary alloy samples. The XPS data of (ReMoV)Se_2_ alloys showed features similar to those of (ReMoV)S_2_ (Figure S5, Supporting Information). The oxidation number of V atoms is lower for the ternary alloy samples than for their Re_1‐_ *_x_* V*_x_* Se_2_ binary counterparts.

The rightmost panel of Figure 3a corresponds to the valence band spectrum (VBS) of the ternary sulfide alloys. The onset of the VBS can be used to predict the valence band maximum (VBM) below the Fermi energy level (E_F_), which corresponds to zero binding energy. All the samples showed VBM = 0 eV owing to the metallic phase. Band integration of the VBS from 0 to 10 eV provided the *d‐*band center (ɛ_d_). The ɛ_d_ value shifted slightly toward E_F_ (−3.7 eV → −3.5 eV vs E_F_) with increasing *x*_V_. The ternary alloys exhibited smaller (negative) ɛ_d_ values compared to Re_1‐_ *_x_* V*_x_* S_2_ (–4.2 − –3.8 eV), indicating a more metallic character (Figure S5, Supporting Information). Ternary alloying of selenide also lifted the ɛ_d_ value (–3.3 − –3.2 eV) toward E_F_, where Re_1‐_ *_x_* V*_x_* Se_2_ has ɛ_d_ = –3.8 − –3.2 eV, with VBM = 0 eV. The XPS analysis results are summarized as follows. Metallicity increased with *x*_V_, similar to that of the Re_1‐_ *_x_* V*_x_* X_2_ binary alloys. The ternary alloy nanosheets are more metallic and less oxidized than the binary alloys.

Figure 3b shows the EXAFS above the Re L_3_‐edge, Mo K‐edge, and V‐K edge for ReS_2_, MoS_2_, VS_2_, and their ternary alloy samples. The corresponding *k*‐ and *R*‐space wavelet‐transform (WT) EXAFS spectra are also plotted with the data for Re, Mo, and V foils for comparison. In the X‐ray absorption near edge structure (XANES) of Re and Mo, the alloy samples exhibited a lower intensity of the white line than their unitary counterparts, which is correlated with their more metallic character. As *x*_V_ increases to 0.5 (sample **7**S), the intensity of Re L_3_‐edge and Mo K‐edge decreases, indicating an increase in metallicity. The increased intensity at *x*_V_ = 0.8 could be due to the depleted electron density by oxidation. In the XANES of V K‐edge, two pre‐edge peaks appear at 5.47 keV (marked by **A**, originated from 1*s* → 3*d* transition) and 5.48 keV (marked by **B**, 1*s* → 4*s*‐4*p* hybrid orbitals). Compared to VS_2_, the alloy samples exhibited a reduced intensity of the **A** peak, probably owing to the larger number of electrons occupying the 3*d* orbitals of the more metallic alloys. The increased intensity of the **B** peak can be explained by better structural symmetry due to less oxidation.

The Fourier‐transform extended XAFS (FT EXAFS) results show the metal–S bonding peaks. The Re‐S and Mo‐S peaks showed lower intensities than those of the unitary samples, probably owing to their reduced number of bonds and lower crystallinity. By contrast, the higher intensity of the V‐S peak resulted from lower oxidation, which increased the number of V─S bonds. Figure S6 and Table S3 (Supporting Information) summarize the fitting curves and fitted values, respectively. The value of *d*_Re‐S_ decreases from 2.39 Å in ReS_2_ to 2.38 Å in **10**S, and the average value of *d*_Mo‐S_ is 2.41 Å for all compositions. The WT EXAFS data showed no shift of the Re‐S peak at *k* = 6.5 Å and the Mo‐S peak at *k* = 5 Å, suggesting negligible changes in electronic structures at the Re and Mo sites, respectively. In the alloy samples, *d*_V‐S_ is ≈2.32 Å, whereas that in VS_2_ is 2.29 Å. In the *k*‐space of WT EXAFS, samples **4**S and **7**S show the V‐S peak at 5–6 Å^−1^, which is shifted from that of **10**S and VS_2_ at 4 Å^−1^. Because the S atom is heavier than the O atom, it induces a large scattering amplitude at high *k*. Hence, this peak shift is ascribed to lower oxidation, which is consistent with the larger *d*_V‐S_ value.

**Figure 4:**
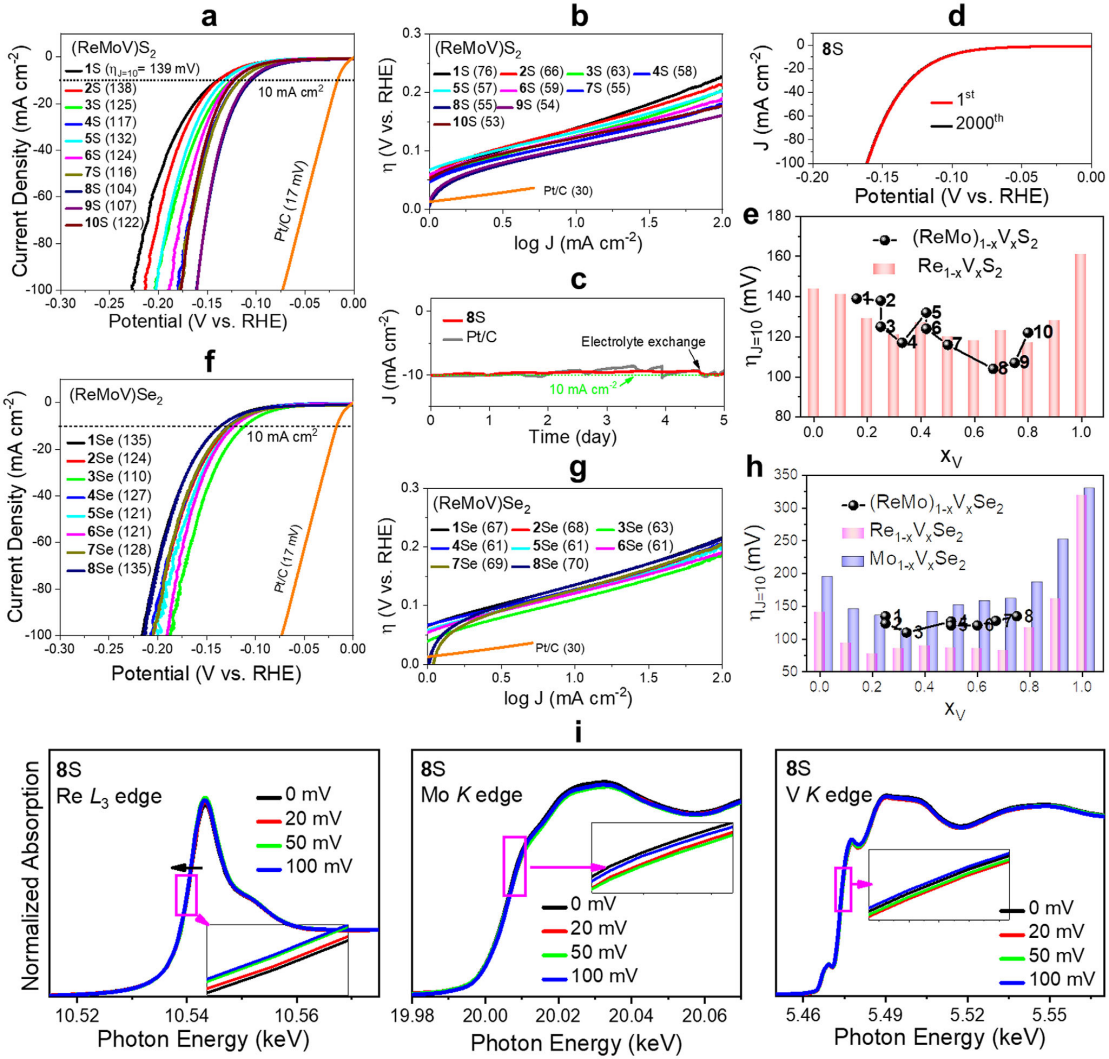
a) LSV curves (scan rate: 2 mV s^−1^) versus RHE for (ReMoV)S_2_ samples, and Pt/C toward HER in H_2_‐saturated 0.5 m H_2_SO_4_. The values in parenthesis correspond to η*_J_*_= 10_. b) Tafel plots (η vs log *J*) derived from the LSV curves, based on the equation η = *b* log(*J*/*J*_0_), where *b* is the Tafel slope, and *J*_0_ is the exchange current density (extrapolated value at η = 0). Linear fit provides the *b* values (in parenthesis). c) CA responses of *x*_V_ = 0.67 (sample **8**S) and Pt/C at η_J = 10_ (104 mV) for 5 days and d) comparison of 1st and 2000th cycled LSV curves. e) η_J = 10_ versus *x*_V_ for (ReMo)_1‐_ *_x_* V*_x_* S_2_ (sphere symbols) and Re_1‐_ *_x_* V*_x_* S_2_ samples (columns, ref. [20]). f) LSV curves and g) Tafel plots for (ReMoV)Se_2_ samples. The corresponding η_J = 10_ and *b* values are listed in the parentheses. h) η_J = 10_ versus *x*_V_ for (ReMo)_1‐_ *_x_* V*_x_* Se_2_ (sphere symbols), Re_1‐_ *_x_* V*_x_* Se_2_ ref. [19]/Mo_1‐_ *_x_* V*_x_* Se_2_ ref. [14] samples (columns). i) Ex situ XANES above the Re L_3_‐edge (10.53 keV), Mo K‐edge (20.01 keV), and V K‐edge (5.46 keV) for *x*_V_ = 0.67 (sample **8**S) after applying an overpotential (𝜂 = 0–100 mV vs RHE) under HER conditions (H_2_‐saturated 0.5 m H_2_SO_4_).

Table S4 (Supporting Information) summarizes the electrocatalytic parameters of the ternary alloy and unitary samples toward the HER in an acidic electrolyte (0.5 M H_2_SO_4_). **Figure** **4**a displays linear sweep voltammetry (LSV) curves of the sulfide samples ((ReMoV)S_2_), namely the current density (*J*) plotted as a function of the applied potential (vs reversible hydrogen electrode (RHE)). The applied potential is equal to the overpotential (η), and η at *J* = 10 mA cm^−2^ is denoted as η_J = 10_. All alloy samples have lower η_J = 10_ values (104–138 mV) than those of ReS_2_ (144 mV), MoS_2_ (172 mV), and VS_2_ (161 mV) (see Figure S7, Supporting Information). In Figure 4b, the Tafel plot (η vs log *J*) provided the Tafel slope (*b*) by a linear fit. Most of the alloy samples exhibited lower *b* values (53–76 mV dec^−1^) than those of ReS_2_ (103 mV dec^−1^), MoS_2_ (54 mV dec^−1^), and VS_2_ (77 mV dec^−1^). As a reference, we also measured the η_J = 10_ and *b* values of commercial 20 wt.% Pt/C to be 17 mV and 30 mV dec^−1^, respectively. The best HER performance was observed for sample **8**S (*x*_V_ = 0.67) with η_J_ *_=_*_10_ = 104 mV. The enhancement effect of alloying is consistent with previous observations of (MoNbV)Se_2_ and (MoWV)Se_2_ ternary alloys and many binary alloys (see Table S5, Supporting Information).^[^ ^7^, ^8^, ^9^, ^10^, ^11^, ^12^, ^13^, ^14^, ^15^, ^16^, ^17^, ^18^, ^19^, ^20^, ^21^, ^22^ ^]^

The charge transfer resistance (*R*_ct_) and double‐layer capacitance (*C*_dl_) were measured using electrochemical impedance spectroscopy and cyclovoltammetry (Figures S8 and S9, Supporting Information, respectively). The *R*_ct_ and *C*_dl_ values showed a good correlation with the LSV data. Therefore, the enhanced HER performance of the ternary alloys is due to more efficient charge transfer and larger double‐layer capacitance. The electrochemically active surface area (ECSA) was estimated using *C*_dl_, as described in the Supporting Information (Experimental Section). In Figure S7 (Supporting Information), the LSV data were plotted using *J*_ECSA_ (defined as *J* divided by ECSA), showing the enhanced HER performance of ternary alloys compared to ReS_2_ and MoS_2_. The ternary alloying with *x*_V_ = 0.6–0.8 (samples **8**S and **9**S) is consistently most effective in enhancing the HER performance. The chronoamperometry (CA) data of the best sample **8**S show negligible current attenuation at η_J = 10_ after 5 days, which is like Pt/C (Figure 4d). The current level recovered when a fresh electrolyte was used after 4.6 days, indicating that the current decrease came from the pH change. The LSV curve after the 2000th scan showed negligible change (Figure 4e). The XRD, EDX, and XPS data confirmed that both the crystal structure and composition were retained after the CA test (Figure S10, Supporting Information). Furthermore, we evaluated the Faradaic yield (FY) and turnover frequency (TOF) at η = 0.15 V as described in the Supporting Information. The TOF value was estimated as 0.017 H_2_ s^−1^ based on FY = 96% (Supporting Information).

In Figure 4e, the η_J_ *_=_*_10_ value as a function of *x*_V_ is overlaid on the data for Re_1‐_ *_x_* V*_x_* S_2_.^[^ ^20^ ^]^ As *x*_V_ increases, the HER performance is enhanced owing to the increased metallicity. However, due to sample oxidation at higher *x*_V_, the best HER performance was probably observed for sample **8**S. The ternary alloys at *x*_V_ = 0.67 (sample **8**S) and 0.75 (sample **9**S with η_J_ *_=_*_10_ = 107 mV) have lower η_J_ *_=_*_10_ than the values of Re_1‐_ *_x_* V*_x_* S_2_ (117–128 mV at *x*_V_ = 0.6–0.8). At the same *x*_V_ value, the Re‐rich samples are always more HER‐active than the Mo‐rich ones;, *i.e*., η_J_ *_=_*_10_ = 138 and 125 mV for sample **2**S (0.25, 0.5, 0.25) and **3**S (0.50, 0.25, 0.25), and η_J_ *_=_*_10_ = 132 and 124 mV for samples **5**S (0.16, 0.42, 0.42) and **6**S (0.42, 0.16, 0.42), respectively. The HER performance data for the annealed binary/ternary samples are plotted in Figures S8,S9 and S11 (Supporting Information), showing a similar feature to that of as‐grown samples. The *d*‐band center (ɛ_d_) theory has often been used to explain optimal HER performance.^[^ ^48^, ^49^, ^50^ ^]^ As the ɛ_d_ of the catalyst shifts closer to E_F_ (i.e., becomes less negative), the catalyst‐H interaction becomes stronger owing to the more metallic character. As shown in Figure S5 (Supporting Information), the ɛ_d_ of the ternary alloys is closer to E_F_ than that of the binary alloys, suggesting that the ternary alloying increased the metallicity than the binary alloys. Therefore, the alloying of (ReV)S_2_ with MoS_2_ enhanced the HER performance because of the increased metallicity.

Figure 4f shows the LSV curves of the (ReMoV)Se_2_ ternary alloy samples. All alloys exhibited η_J = 10_ = 110–136 mV. The Tafel slope *b* was 61–70 mV dec^−1^ (Figure 4g). Those values are lower than those of ReSe_2_ (141 mV and 67 mVdec^−1^), MoSe_2_ (130 mV and 73 mVdec^−1^), and VSe_2_ (320 mV and 103 mVdec^−1^), as shown in Figure S12 (Supporting Information), indicating the positive effects of the ternary alloying. The HER performance of selenide alloys was less dependent on the composition than that of the sulfide alloys. The *R*_ct_ and *C*_dl_ values did not change significantly (Figures S8 and S9, Supporting Information). Figure 4h compares the η_J_ *_=_*_10_ values at different *x* for the (ReMo)_1‐_ *_x_* V*_x_* Se_2_, Re_1‐_ *_x_* V*_x_* Se_2_, and Mo_1‐_ *_x_* V*_x_* Se_2_ samples.^[^ ^14^, ^19^ ^]^ These ternary alloy samples have a lower HER performance than the Re_1‐_ *_x_* V*_x_* Se_2_ samples, while they show much enhancement compared to Mo_1‐_ *_x_* V*_x_* Se_2_. Figure S12 (Supporting Information) shows the *J*_ECSA_ data for these (ReMoV)Se_2_ samples, consistently showing less enhancement effect than that of Re_1‐_ *_x_* V*_x_* Se_2_.

As shown in Figure S5 (Supporting Information), the ternary alloying shifts the ɛ_d_ values closer to E_F_ than that binary alloying. According to Sabatier's rule, the catalytic performance is optimized when the interaction between H and the catalyst has an intermediate strength.^[^ ^48^, ^49^, ^50^ ^]^ If ɛ_d_ is too negative or less negative, the catalyst‐H interaction would be too weak or strong. Ternary alloying lowers ɛ_d_ to less negative values (–3.2−–3.3 eV), and thus, the interaction becomes stronger owing to excessive metallicity. Another possible model is based on the number of active sites. As we discussed in our previous work on Re_1‐_ *_x_* V*_x_* Se_2_, the Se vacancies on the Re atoms are the HER active sites.^[^ ^19^ ^]^ The lower HER performance than that of Re_1‐_ *_x_* V*_x_* Se_2_ is due to the decreased concentration of active Re sites via the alloying with MoSe_2_. The higher HER performance than that of Mo_1‐_ *_x_* V*_x_* Se_2_ is due to the increased concentration of active Re sites via the alloying with ReSe_2_.

Ex situ XAFS measurements were performed on sample **8**S after applying an overpotential (0–100 mV) under the HER conditions to gain insight into the electronic structures around the metal atoms during the HER. Figure 4i shows the XANES above the Re L_3_‐, Mo K‐, and V K‐edge. The Re L_3_‐edge was redshifted as 𝜂 increased to 100 mV (see inset). The Mo and V peaks also showed redshifts as 𝜂 increased to 100 mV, but less significantly compared to the Re L_3_‐edge. Nevertheless, all metal atoms became more metallic as 𝜂 increased. The FT EXAFS data with the fitting curves and fitted values are displayed in Figure S6 and Table S3 (Supporting Information). The interatomic distance of Re‐S increased due to the reduction, while that of Mo‐S and V‐S changed very little. This suggests that the Re atoms improve more effectively the HER activity compared to the Mo atoms. This model can explain why the Re‐rich samples are more HER active than the Mo‐rich ones. Furthermore, the less active Mo sites also support our model for the less enhancement of (ReMoV)Se_2_ samples.

**Figure 5:**
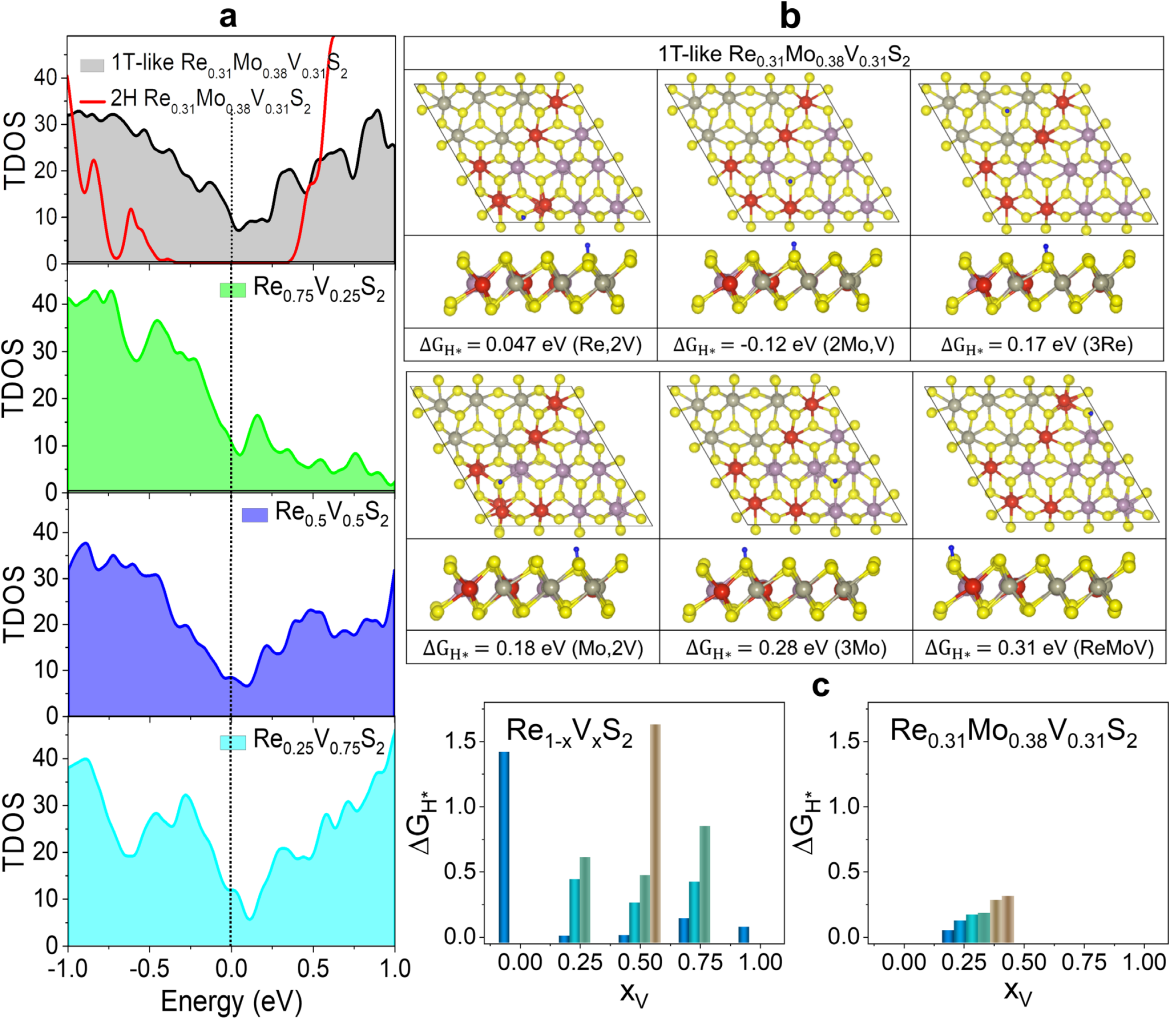
a) TDOS near the Fermi level for 2H and 1T‐like phases of Re_0.31_Mo_0.38_V_0.31_S_2_, Re_0.75_V_0.25_S_2_, Re_0.5_V_0.5_S_2_, and Re_0.25_V_0.75_S_2_ ref. [20]. The Fermi level (black line) is set to zero. b) Geometry of H adsorption on Re_0.31_Mo_0.38_V_0.31_S_2_. The ΔG_H*_ value is given for each site. Gray, violet, red, and yellow balls represent Re, Mo, V, and S atoms, respectively. The small blue ball represents the adsorbed H atom. c) Histograms representing ΔG_H*_ for various S sites of Re_1‐_ *_x_* V*_x_* S_2_ ref. [20] and Re_0.31_Mo_0.38_V_0.31_S_2_ models.

For the Re_0.31_Mo_0.38_V_0.31_S_2_ model, the total electronic density of states (TDOS) near the Fermi level (E_F_) was calculated, as shown in **Figure** **5**a. The 1T‐like Re_0.31_Mo_0.38_V_0.31_S_2_ ternary alloy exhibits a significant TDOS at E_F_, indicating metallicity. The 2H phase alloy is a semiconductor. For comparison, Figure 5a displayed the TDOS of binary alloys Re_0.25_V_0.75_S_2_, Re_0.5_V_0.5_S_2_, and Re_0.25_V_0.75_S_2_, which were reported in our previous work.^[^ ^20^ ^]^ As *x*_V_ increases, the TDOS at E_F_ > 0 increases significantly, which could be related to the higher metallicity. Compared to the binary alloys with *x*_V_ = 0.25 and 0.5, the ternary alloy at *x*_V_ = 0.31 shows a higher TDOS at E_F_ > 0. Therefore, the incorporation of MoS_2_ enhances the metallicity, which is consistent with the experimental results.

The Gibbs free energy change for H adsorption (|ΔG_H*_|) is a well‐known parameter to predict the optimal HER activity using the condition of |ΔG_H*_| ≈ 0. For ReS_2_, the basal S atoms are not favorable for H adsorption (ΔG_H*_ = 1.42 eV).^[^ ^10^, ^20^ ^]^ The basal S atoms of 1T VS_2_ are HER active, in contrast, with ΔG_H*_ = 0.072 eV.^[^ ^20^ ^]^ In the case of VS_2_, significant oxidation reduces the HER performance. As shown in Figure 5b, alloying with 1T‐like phase alloy activates six different S sites with ΔG_H*_ = 0.047–0.31 eV. The S atoms coordinated with (Re, 2 V), (2Mo, V), (3Re), (Mo, 2V), (3Mo), and (ReMoV) are all HER‐active. In the case of (ReV)S_2_ binary alloys, the S atoms coordinated with (3V) or (Re, 2V) are HER‐active, but many of the S atoms coordinated with (3Re) or (2Re, V) are not.^[^ ^20^ ^]^ The ΔG_H*_ values are summarized in Figure 5c, suggesting that the ternary alloys possess more HER active sites than binary alloys, probably due to the more metallicity. Furthermore, the S site coordinated with Re has a higher HER activity than that coordinated with Mo: ΔG_H*_ = 0.047 and 0.18 eV for (Re, 2V) and (Mo, 2V) coordination, and ΔG_H*_ = 0.17 and 0.28 eV for (3Re) and (3Mo) coordination, respectively. This shows that compared to Mo, Re can more effectively activate the coordinated S atoms, which is consistent with the experimental results.

## 3 Conclusion

We synthesized (ReMoV)S_2_ ternary alloy nanosheets with full composition tuning using a solvothermal reaction and (ReMoV)Se_2_ nanosheets using a colloidal reaction. An increase of *x*_V_ (from 0.16 to 0.80) induced a phase change into a metallic 1T phase. Atomically resolved STEM images revealed a homogeneous mixing of the three metals at the atomic scale in the 1T phase. Spin‐polarized DFT calculations predicted consistently that ternary alloying produces the metallic 1T‐like structure. The XPS and XAFS data that the alloying of (ReV)X_2_ with MoX_2_ produced a more metallic phase with less oxidation, and the *d*‐band center (ɛ_d_) more closely approached E_F_. As *x*_V_ increases, the nanosheets become more metallic. The (ReMoV)S_2_ nanosheets exhibited enhanced HER catalytic activity in 0.5 m H_2_SO_4_, characterized by η_J_ *_=_*_10_ = 104 mV versus RHE at *x*_V_ = 0.67. For the (ReMoV)Se_2_ nanosheets, less enhancement of HER was observed. The enhanced HER performance of (ReMoV)S_2_ was ascribed to the increased metallicity. Ex situ EXAFS data suggests that the metallicity of Re atoms improves the catalytic activity of (ReMoV)S_2_. The calculated ΔG_H*_ showed that ternary alloying effectively optimized H adsorption on the basal S atoms, supporting the experimental HER data.

## 4 Experimental Section

### Synthesis of (ReMoV)X_2_ Nanosheets

Ammonium perrhenate (NH_4_ReO_4_), Bis(acetylacetonato)dioxo molybdenum (VI) (MoO_2_(acac)_2_, MoO_2_(C_5_H_7_O_2_)_2_), vanadyl (IV) acetylacetonate (VO(acac)_2_, VO(C_5_H_7_O_2_)_2_) (total amount: 1 mmol) were prepared as metal precursors. For sulfide nanosheets, metal precursor and thioacetamide (CH_3_CSNH_2_, 8 mmol) were dissolved in 1‐methyl‐2‐pyrrolidinone (10 mL) and reacted in a Teflon‐lined stainless steel autoclave reactor at 240 °C for 12 h. For selenide nanosheets, metal precursor and 2 mmol of dibenzyl diselenide ((PhCH_2_)_2_Se_2_) were dissolved in 5 mL of oleylamine (OAm) and was degassed at 70 °C. 5 mL of oleylamine (OAm; C_18_H_35_NH_2_) in a three‐necked flask was degassed and heated at 260 °C. The precursor solution (2 mL) was injected into the preheated OAm with an injection rate of 0.4 mL min^−1^ for 5 min, and the mixture was reacted for 1 h. After washing, the sulfide/selenide products were annealed in a quartz tube inside an electrically heated furnace under Ar flow at 400 °C for 1 h. Detailed experimental and calculation methods are provided in the Supporting Information.

## Conflict of Interest

The authors declare no conflict of interest.

## Supporting information



Supporting Information

## Data Availability

The data that support the findings of this study are available from the corresponding author upon reasonable request.
