# Supplementary material for: (ReMoV)X2 (X = S, Se) Ternary Alloy Nanosheets for Enhanced Electrocatalytic Hydrogen Evolution Reaction
Source: Small. 2025 Apr 24;21(24):2503399. doi: 10.1002/smll.202503399 (PMC12177864; doi:10.1002/smll.202503399)
Supplement: Supplementary file 1 — Supporting Information [file SMLL-21-2503399-s001.docx]

*Supporting Information*

(ReMoV)X_2_ (X = S, Se) Ternary Alloy Nanosheets for Enhanced Electrocatalytic Hydrogen Evolution Reaction

Junaid Ihsan,^†,‡^ Ju Yeon Kim,^†,‡^ In Hye Kwak, ^†,≠^ Irtiqa Mishal,^‡^ Jun Hyeok Choi,^‡^ Jung Eun Ahn,^‡^ Sang-Gil Lee,^≠^ Seung Jo Yoo,^≠^ Ik Seon Kwon,^*,§^ Jeunghee Park^*,‡^ and Hong Seok Kang,^*,⁑^

^‡^ Department of Advanced Materials Chemistry, Korea University, Sejong 339-700, Republic of Korea; *E-mail: [parkjh@korea.ac.kr](mailto:parkjh@korea.ac.kr)

^§^ Department of Energy Science & Engineering, Kunsan National University, 558 Daehak-ro, Gunsan, Republic of Korea; *E-mail: [iskwon@kunsan.ac.kr](mailto:iskwon@kunsan.ac.kr)

^≠^ Research Center for Materials Analysis, Korea Basic Science Institute (KBSI), Daejeon 34133, Republic of Korea

^⁑^ Department of Nano and Advanced Materials, Jeonju University, Chonju, Chonbuk 55069, Republic of Korea; *E-mail: [hsk@jj.ac.kr](mailto:hsk@jj.ac.kr)

^†^ J. Ihsan, J. Y. Kim, and I. H. Kwak contributed equally as the first author.

**Contents**

**Experimental Section**

**Table S1**. Composition of (ReMoV)S_2_ and (ReMoV)Se_2_ nanosheets.

**Table S2**. Parameters of (4×4×1) supercells.

**Table S3**. Fitting parameters of EXAFS data.

**Table S4**. Summary of HER performance of (ReMoV)S_2_ and (ReMoV)Se_2_ nanosheets.

**Table S5**. Comparison of HER performance with the previous works.

**Figure S1**. SEM, HRTEM, and EDX data of (ReMoV)S_2_ nanosheets.

**Figure S2**. SEM, HRTEM, and EDX data of (ReMoV)Se_2_ nanosheets.

**Figure S3**. XRD data of as-grown and annealed (ReMoV)S_2_ nanosheets.

**Figure S4.** Crystal structure of (4×4×1) supercells of Re_0.31_Mo_0.38_V_0.31_S_2_.

**Figure S5**. XPS data of (ReMoV)S_2_ and (ReMoV)Se_2_ nanosheets.

**Figure S6**. Fitting curves of EXAFS data.

**Figure S7**. HER data of ReS_2_, MoS_2_, VS_2_, and (ReMoV)S_2_ nanosheets

**Figure S8**. Nyquist plots of (ReMoV)S_2_ and (ReMoV)Se_2_ nanosheets.

**Figure S9**. Cyclic voltammetry curves of (ReMoV)S_2_ and (ReMoV)Se_2_ nanosheets.

**Figure S10**. Characterization of sample **8**(S) after CA test.

**Figure S11**. HER data of annealed (ReMoV)S_2_ nanosheets and analysis.

**Figure S12**. HER data of ReSe_2_, MoSe_2_, VSe_2_, and (ReMoV)Se_2_ nanosheets

**References**

**I. Experimental Section**

***Reagents***: Chemicals were purchased from Sigma-Aldrich or Alfa Co.

***Solvothermal Synthesis of Sulfide Nanosheets****.* 1 mmol of (NH_4_)ReO_4_ (ammonium perrhenate (VII), molecular weight (MW) = 268.2359 g/mol, 99%), MoO_2_(C_5_H_7_O_2_)_2_  (Bis(acetylacetonato) dioxo molybdenum (VI), MoO_2_(acac)_2_, MW = 326.15 g/mol, 99%), and VO(C_5_H_7_O_2_)_2_ (Vanadyl acetylacetonate (bis(2,4-pentanedionato) vanadium (IV) oxide, VO(acac)_2_, MW= 265.16 g/mol) mixture and 8 mmol of CH_3_CSNH_2_ (thioacetamide, TA, MW = 75.13 g/mol) was dissolved in 10 mL of N-methyl-2-pyrrolidone (NMP, MW = 99.13 g/mol, bp = 202 °C) *via* stirring (400 rpm) for 1 h. The molar ratio of NH_4_ReO_4_, MoO_2_(acac)_2_ and VO(acac)_2_ was varied by keeping a total 1 mmol. The reaction mixture was transferred to a Teflon-lined stainless steel autoclave reactor. A solvothermal reaction was performed at 240 °C for 10 h in an electric oven. The product was collected by centrifugation, washed thoroughly with deionized anhydrous ethanol (HPLC grade) by several times, and then vacuum-dried at room temperature. Sulfur anions (S^2−^) are prepared directly by the decomposition reaction of TA. The Re^7+^ and Mo^6+^ ions are reduced to +4 charged cations by non-bonding electrons of N atoms in TA. The product (powders) was placed in a quartz tube inside electrically heated furnace and annealed under Ar flow (flow rate = 200 sccm) at 400 °C for 1 h.

***Colloidal Synthesis of Selenide Nanosheets****.* A hot injection colloidal reaction was conducted using Schlenk line under Ar flow. 5 mL of oleylamine (OAm; C_18_H_35_NH_2_; molecular weight (MW) = 267.493 g mol^-1^, technical grade 70%, density = 0.813 g mL^-1^) in a three-necked flask was degassed at 120 °C for 30 min, then the temperature was raised to 220-340 °C. 0.5 mmol of NH_4_ReO_4_, MoO_2_(acac)_2_, and VO(C_5_H_7_O_2_)_2_ mixture, and 1 mmol of (PhCH_2_)_2_Se_2_ (dibenzyl diselenide; MW = 340.2 g mol^-1^, 95%) were dissolved in 5 mL OAm and the mixture was kept at 70 °C for 2 h. The molar ratio of metal precursors was varied by keeping a total 0.5 mmol. 2 mL of precursor solution was injected into the OAm solution in flask (prepared with an injection rate of 0.4 mL min^-1^ for 5 min, and the mixture was stirred at 260 °C. Total reaction time is 1 h. The reaction solution was cooled down to room temperature and the black products was separated by centrifugation. The products were washed with 1:1 (v/v) ethanol:toluene mixed solvent for four times, and dried using an evaporator. The product (powders) was placed in a quartz tube inside electrically heated furnace and annealed under Ar flow (flow rate = 200 sccm) at 400 °C for 1 h.

***Characterization****.* The products were characterized by high-resolution scanning electron microscopy (SEM, Jeol), field-emission transmission electron microscopy (FE TEM, Libra 200 MC TEM, Carl Zeiss), and high-voltage transmission electron microscopy (HV-TEM, Jeol JEM ARM 1300S, 1.25 MV). Energy-dispersive X-ray fluorescence spectroscopy (EDX) with elemental maps was measured using an Ultra Corrected Energy Filtering TEM operated at 200 kV that equipped with ZrO/W-field emitter system (Schottky emitter), EDS detector system (X-Max 80T, Oxford), and side CCD camera (ORIUS SC200D. Gatan). Fast Fourier-transform (FFT) images were generated by the inversion of the TEM images using Digital Micrograph GMS1.4 software (Gatan Inc.).

Spherical Aberration (Cs)-corrected scanning TEM (STEM) analysis was carried out using a Titan 80-300TM (FEI, The Netherlands) microscope operated at 200 kV. The STEM convergence semi-angle (α) used was ~18 mrad. The minimum and maximum acceptance semi-angles (β) were ~20 and 122 mrad, respectively. The dwell time per pixel was set to 0.7 µs for imaging. We used the low total dose and dwell time while measuring the STEM. The image was usually taken at a dose rate of 6 × 10^4^ e-/nm^2^·s with a total dose of 1.8 × 10^5^ e-/nm^2^. Elemental analysis was performed on an elemental analyzer (Elementar Analysensysteme GmbH, Model No. Elementar Vario EL cube).

High-resolution X-ray diffraction (XRD) patterns were obtained using the 9B beamlines of the Pohang Light Source (PLS)-II with monochromatic radiation (λ = 1.52150 Å). XRD pattern measurements were also carried out in a Rigaku D/MAX-2500 V/PC using Cu K_α_ radiation (λ = 1.54056 Å). X-ray photoelectron spectroscopy (XPS) measurements were performed using the 10A2 and 8A1 beam lines of the PLS-II. The photon energy of synchrotron radiation was calibrated using the standard sample; Au film, Au 4*f*_7/2_ peak at 84.0 eV. X-ray absorption near edge structure (XANES) measurements were performed using at the 8C nanoprobe XAFS beamline (BL8C) and 7D beam line of the PLS-II. The X-ray beam was monochromated using a Si(111) double crystal, where the harmonic rejection was attained by detuning the beamline optics so that the intensity of the incident beam was reduced by 30%. For all the measurements, the slits had an opening of 0.5 mm (vertical) × 1 mm (horizontal). All the measurements were performed at room temperature in transmission mode and the detectors were ionization-chamber-based. For the Re L_3_-edge, Mo K-edge, and V K-edge measurements, the monochromator energies were calibrated using Re, Mo, and V foils, respectively. XANES analysis and EXAFS fitting were performed using the Athena and Artemis packages, which provide an interface to IFEFFIT.

**Electrochemical Measurements.** Experiments were carried in a three-electrode cell connected to an electrochemical analyzer (CompactStat, Ivium Technologies). HER electrocatalysis in 0.5 M H_2_SO_4_ electrolyte was measured using a linear sweeping from 0 to -0.6 V (vs. RHE) with a scan rate of 2 mV s^–1^. A saturated calomel electrode (SCE, KCl saturated, Pine Instrument) was used as a reference electrode. A Pt coil (with fritted glass) was used as a counter electrode. The electrolyte was purged with H_2_ (ultrahigh grade purity 99.999%) during the measurement. The Pt counter electrode was encapsulated with a fritted glass tube (Figure E1), so that the Pt deposition on the working electrode was prevented.


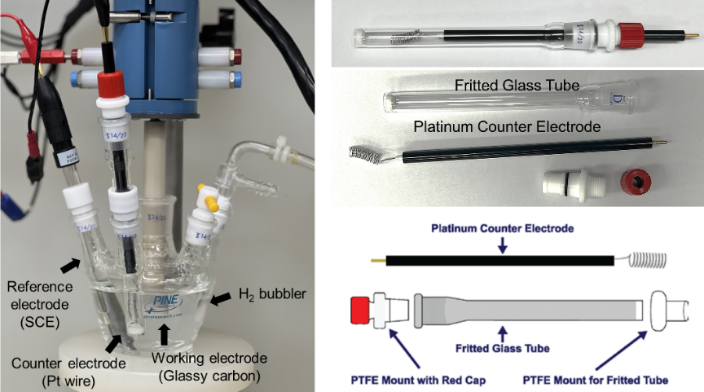


**Figure E1.** Photographs for our three-electrodes cell and Pt counter electrode encapsulated with a fritted glass tube, as shown on the right panel.

The applied potentials (E) reported in our work were referenced to the reversible hydrogen electrode (RHE) through standard calibration as described elsewhere. We calibrate the potential of the reference electrode *vs.* standard hydrogen electrode (SHE). Cyclic voltammetry (CV) curves were obtained at a scan rate of 2 mV s^−1^, in the high-purity H_2_ saturated electrolyte with a Pt wire as the working electrode. The average value of the potential at which the current crossed at zero was -0.278 V. Therefore E (*vs.* RHE) = E (*vs.* SCE) + 0.278 V.

4 mg sample was mixed with 2 mg carbon black (Vulcan XC-72) dispersed in Nafion (50 μL) and isopropyl alcohol (0.95 mL). The catalyst materials (0.65 mg cm^-2^) were deposited on a glassy carbon rotating disk electrode (RDE, area = 0.1963 cm^2^, Pine Instrument),), and a rotation speed of 1600 rpm was used for the linear sweep voltammetry (LSV) measurements. The Pt/C (20 wt.% Pt in Vulcan carbon black, Sigma-Aldrich) tested as reference sample using the same procedure. The LSV curves were reproducible for four separate loadings of samples on the GC RDE electrode. For chronoamperometric stability test and *ex-situ* XAFS measurements, we fabricated the working electrode by depositing the samples (1 mg cm^-2^) on 1 × 1 cm^2^ area of hydrophilic/waterproof carbon cloth (WIZMAC Co., thickness = 0.35 mm, through-plane resistance = 1 mΩ) that was cut with a size of 1 × 3 cm^2^. The *ex situ* XAFS samples were prepared after applying the potential (0, 20, 50, and 100 mV vs. RHE) for 20 min. The samples were washed with ethanol and DI water several times and dried at room temperature.

Electrochemical impedance spectroscopy (EIS) measurements were carried out for the electrode in an electrolyte by applying an AC voltage of 10 mV in the frequency range of 100 kHz to 0.1 Hz at a bias voltage of ~~-~~0.10 V (*vs.* RHE). To measure double-layer capacitance via CV, a potential range in which no apparent Faradaic processes occur was determined from static CV. All measured current in this non-Faradaic potential region is assumed to be due to double-layer capacitance. The charging current, *i_c_*, is then measured from CVs at multiple scan rates. The working electrode was held at each potential vertex for 10 s before beginning the next sweep. The charging current density (*i_c_*) is equal to the product of the scan rate (ν) and the electrochemical double-layer capacitance (C*_dl_*), as given by equation *i_c_* = ν C*_dl_*. The difference (ΔJ_0.15_) between the anodic charging and cathodic discharging currents measured at 0.15 V or 0.05 V (vs. RHE) was used for *i_c_*. Thus, a plot of ΔJ_0.15_ as a function of ν yields a straight line with a slope equal to 2 × C*_dl_*. The scan rates were 20−100 mV s^-1^.

***ECSA calculation****.* To estimate the actual electrochemical surface area (ESCA), we used the *C*_dl_ value. The roughness factor (= ECSA) is defined as the surface area ratio between the catalyst and the flat TMD electrodes. This can be obtained using$\frac{C_{dl}}{C_{s}} ,$where C*_dl_* and C*_s_* are electrochemical double-layer capacitance of the catalysts (measured by the procedure described above) and the flat surface, respectively. The *C_s_* value is assumed to be 0.060 mF cm^-2^ for all samples.^S1a^ The specific surface of the electrode can be estimated by multiplying the geometrical surface (A_geom_ = 0.1963 cm^2^) to ECSA; A_geom_ × ECSA.

**Table E1**. ECSA calculated using the equation of ECSA = *C_dl_*/*C*_s_, where *C_s_* is the flat standard capacitor (60 μF cm^-2^).

(a) (ReMoV)S_2_

| No. | Composition | | | As grown | | Annealed | |
| --- | --- | --- | --- | --- | --- | --- | --- |
|  | Re | Mo | V | *C_dl_* (mF cm^-2^) | ECSA | *C_dl_* (mF cm^-2^) | ECSA |
| ReS_2_ | 1 | 0 | 0 | 28.63 | 477.17 | 18.00 | 300.00 |
| MoS_2_ | 0 | 1 | 0 | 44.07 | 734.50 | 20.58 | 343.00 |
| VS_2_ | 0 | 0 | 1 | 6.17 | 102.83 | 11.40 | 190.00 |
| 1 | 0.42 | 0.42 | 0.16 | 27.87 | 464.50 | 6.59 | 109.83 |
| 2 | 0.25 | 0.5 | 0.25 | 35.14 | 550.66 | 6.81 | 113.50 |
| 3 | 0.50 | 0.25 | 0.25 | 39.68 | 660.16 | 21.44 | 357.33 |
| 4 | 0.33 | 0.33 | 0.33 | 45.74 | 762.416 | 22.54 | 375.67 |
| 5 | 0.16 | 0.42 | 0.42 | 49.46 | 824.41 | 20.00 | 333.33 |
| 6 | 0.42 | 0.16 | 0.42 | 49.67 | 811.25 | 39.42 | 657.00 |
| 7 | 0.25 | 0.25 | 0.50 | 47.82 | 730.16 | 24.77 | 412.83 |
| 8 | 0.167 | 0.167 | 0.67 | 61.31 | 1021.83 | 55.78 | 929.83 |
| 9 | 0.125 | 0.125 | 0.75 | 52.96 | 882.66 | 58.52 | 975.33 |
| 10 | 0.10 | 0.10 | 0.80 | 38.22 | 637.07 | 19.49 | 324.83 |

(b) (ReMoV)Se_2_

| No. | Composition | | |  | |
| --- | --- | --- | --- | --- | --- |
|  | Re | Mo | V | *C_dl_* (mF cm^-2^) | ECSA |
| ReSe_2_ | 1 | 0 | 0 | 3.38 | 56.33 |
| MoSe_2_ | 0 | 1 | 0 | 8.06 | 134.33 |
| VSe_2_ | 0 | 0 | 1 | 1.86 | 31.00 |
| 1 | 0.25 | 0.50 | 0.25 | 14.22 | 237.00 |
| 2 | 0.50 | 0.25 | 0.25 | 12.02 | 200.33 |
| 3 | 0.33 | 0.33 | 0.33 | 15.75 | 262.50 |
| 4 | 0.25 | 0.25 | 0.50 | 13.92 | 232.00 |
| 5 | 0.40 | 0.10 | 0.50 | 11.58 | 193.00 |
| 6 | 0.30 | 0.10 | 0.60 | 12.89 | 214.83 |
| 7 | 0.165 | 0.165 | 0.67 | 12.07 | 201.16 |
| 8 | 0.125 | 0.125 | 0.75 | 14.22 | 237.00 |

***Faradic Efficiency (FE) Measurement***. The hydrogen (H_2_) gas evolution was monitored using gas chromatography (GC, Young Lin YL6500 GC. Thermal conductivity detector (TCD) and ﬂame ionization detector (FID) were equipped with a Molseive 13X column and Porapak N column. The electrolyte was purged with helium gas (≥99.999%). A pulsed discharge detector (VICI, Valco Instruments Co., Inc.) and a GC column (SUPELCO Molecular Sieve 13X) were used. The quantity of H_2_ was calibrated using standard H_2_/He mixtures.


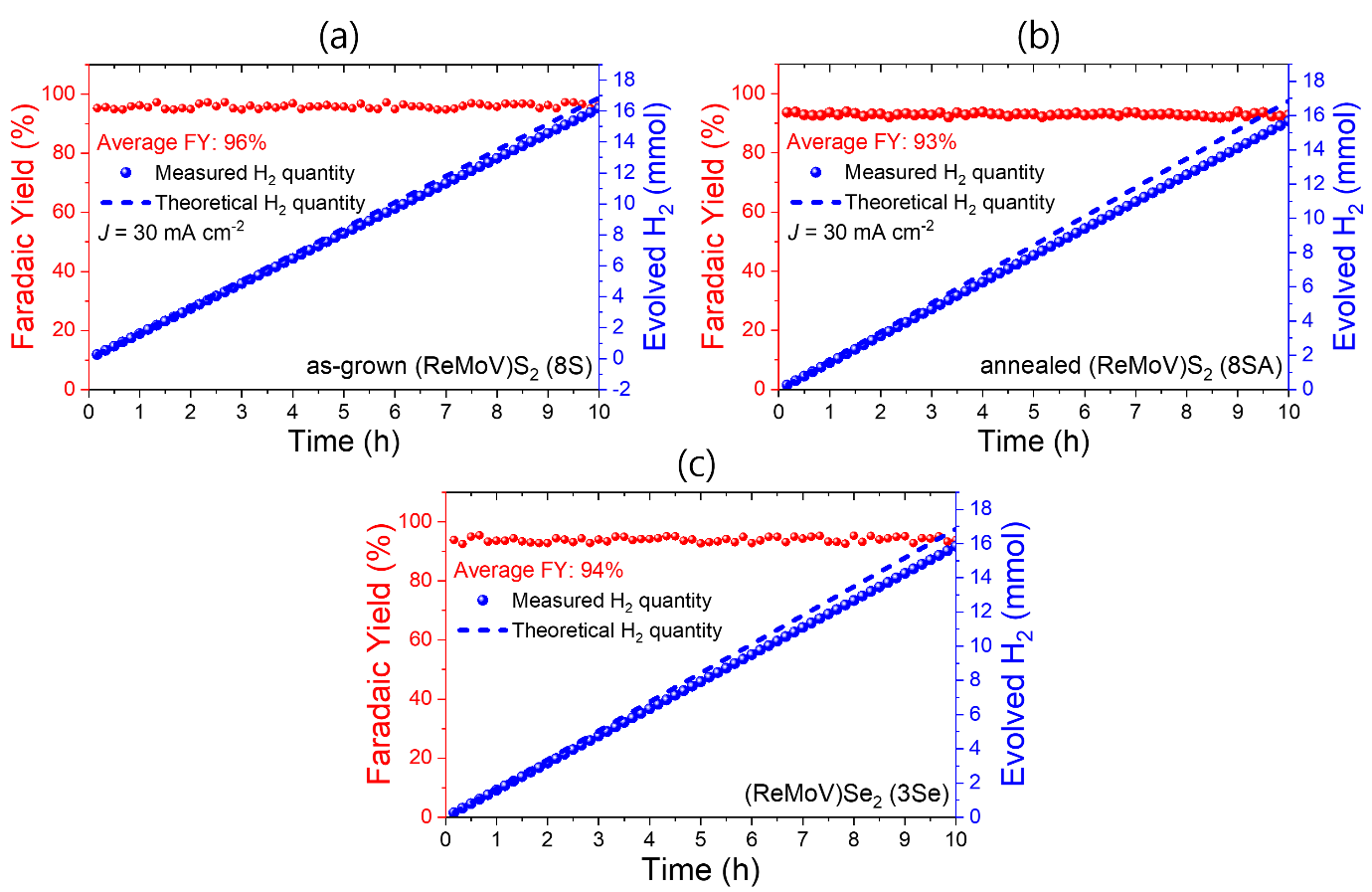
**Figure E2**. Faradaic yield (FY in %) for H_2_ generation anf the corresponding H_2_ evolution (in mmol) *vs.* time, measured for samples (a) **8**S, (b) **8**SA, and (c) **3**Se, and.

**Figure E2** displays the H_2_ evolution data (in mmol) *vs.* time, measured for samples at *J* = 30 mA cm^-2^. The corresponding Faradaic yield (FY) for H_2_ generation was calculated using the equations:$FY (H_{2})= \frac{2 \times N_{H_{2}}\times96485}{Q}$ where *N*_H2_ is the amounts (in mol) of H_2_, and *Q* is the generated charge (= current × time) in Coulomb. The average FY was 96% for **8**S, 93% for **8**SA (annealed **8**S), and 94% for **3**Se (the slash line in the figure represents the calculated H_2_ evolution from the currents assuming FY= 100%).

***TOF Calculation***. The active site density and per-site turnover frequency (TOF) have been estimated as follows. It should be emphasized that since the nature of the active sites of the catalyst is not clearly understood yet and the real surface area for the nanostructured catalyst is hard to accurately determine, the following result is just an estimation.

To estimate the density of active surface site, we used the *ECSA* value. The number of catalytic sites on the surface of flat catalyst can be calculated based on the crystal structure of 1T phase. Using the lattice parameters of 1T-like phase Re_0.31_Mo_0.38_V_0.31_S_2_ (we calculated as *a* = 3.23 Å, *b* = 3.24 Å, γ = 120.18° *≈* 120 °) and assuming one active site (basal S atom) per unit, the density of surface (*N*) active sites is: 1/(0.5 × *a* × *b* ×sin γ) × 10^16^ cm^-2^, where *a* is in a unit of Å.^S1b^ In the case of selenide (**3**Se), the lattice constant is assumed to be *a* = *b* = 3.35 Å, γ = 120 °, based on the XRD data. The density of active sites (*m*) on geometric area is calculated as: *N* × *ECSA*. The TOF can be caculated from the total number of hydrogen gas (H_2_) molecules (*n*_H2_) turns overs at a required potential as follows.

TOF = *n*_H2_/*m* = FY × {*J* (mA cm^-2^) × 3.12 × 10^15^ (H_2_ s^-1^ per mA cm^-2^) × geometrical area of electrode (= 0.1963 cm^2^)}/*m*,

where FY is Faradaic yield (determined by experiment), *n*_H2_ was calculated from the current density (*J*) according to *n*_H2_ = *J* (mA cm^-2^)/1000 mA × 1 (C s^-1^) × 1 mol e^-^/96486 C × 1 mol H_2_/2 mol e^-^ × 6.022 × 10^23^ H_2_ molecules/1 mol H_2_ × electrode area = *J* (mA cm^-2^) × 3.12 × 10^15^ (H_2_ s^-1^) × electrode area (= 0.1963 cm^2^).

We summarized the results in **Table E3**.

**Table E3**. TOF of samples at η = 0.15 V calculated using the density of surface active (*m*), assuming that FY = 96% for **8**S, 93% for **8**SA, and 94% for **3**Se.

| No. | *ECSA* | *N*×10^15^ | *m*×10^15^ | *J* (mA cm^-2^) | FY | *n_H2_*×10^15^ | TOF (H_2_ s^-1^) |
| --- | --- | --- | --- | --- | --- | --- | --- |
| **8**S | 1021.83 | 2.21 | 2258.2 | 65.73 | 0.96 | 38.65 | 0.017 |
| **8**SA | 929.83 | 2.21 | 2054.9 | 20.28 | 0.93 | 11.55 | 0.0056 |
| **3**Se | 262.50 | 2.18 | 572.25 | 37.71 | 0.94 | 21.71 | 0.038 |

***Calculation****.* First-principles calculations were performed through spin-polarized density functional theory (DFT) to investigate how the incorporation of V atoms changes the phase from 1T′′ to 1T. The Vienna *Ab-initio* Simulation Package (VASP)^S2,S3^ is mainly used for this calculations. The electron-ion interactions were described using the projector-augmented wave (PAW)^S4^ method with a plane-wave kinetic energy cutoff of 400 eV. The effect of attractive van der Waals (vdW) interaction was taken into account by employing Grimme’s D3 correction (PBE-D3).^S5^ Structural optimization was performed until the final energy change was < 10^-6^ eV. Both ionic and lattice relaxation was performed using the PBE-D3 exchange-correlation functional and GGA+U method.^S6^ For V, the effective Hubbard parameter (U_eff_ = U - *J* ) was 1.0 eV in accordance with the literature.^S7^ Monkhorst-Pack k-point sampling of 3×3×3 and 3×3×5 was used for the geometry optimization in the 2H and 1T-like phase, respectively.

For the slab geometry, a vacuum space of 20 Å was used along the *Z* (//*c*) direction (perpendicular to the slabs) to ensure that there is no noticeable interaction between periodic images of slabs. The Methfesser-Paxton method with a broadening of 0.1 eV is used for slabs. Total energy of a system was taken by extrapolating the smearing parameter to zero K. The convergence with respect to SCF was attained when the total-energy change between cycles is less than 1×10^-6^ eV. A Monkhorst-Pack *k*-point sampling of 3×3×1 was used.

The change in Gibbs free energy during the reaction provides useful information about the energy and spontaneity of the reaction (whether it can happen without additional energy). Under standard conditions, the HER consists of two steps, the adsorption of H on the catalyst in the Volmer reaction (*), followed by the Heyrovsky/Tafel reaction to release hydrogen molecules (1/2 H_2_ +*). Therefore, we should construct the Gibbs free energy profile along the reaction coordinate by calculating the relative free energies of the reactant and intermediates in the Volmer reaction. The Gibbs free energy profile along the reaction coordinate can be calculated according to the equation:^S8^ $\Delta G= {\Delta E}_{DFT}+{\Delta E}_{ZPE}+ {\Delta H}_{corr}-T\Delta S$**,** where $E_{DFT}$ is the total energy of hydrogen atom adsorption calculated from DFT, $\Delta E_{ZPE}$ is the zero point energy change, ${\Delta H}_{corr}$ is H-correction, *i.e.* the reaction enthalpy change from 0 to 298 K, and $T\Delta S$ is the entropy change between adsorbed hydrogen and hydrogen in the gas phase under standard conditions, based on the Debye model. $H_{corr}=\int_{0}^{298} C_{V}\mathrm{dT}$ was calculated from the vibrational heat capacity using the calculated vibrational frequencies by Harmonic Approximation.^S9^ The H_2_ molecule is treated as an ideal gas, while the adsorbed H is treated using the harmonic approximation. VASP calculations were used to determine the vibrational frequencies of adsorbed H atom on the system.

**Table S1**. Composition (mole fraction and mole ratio) of samples determined using EDX data. The mole fraction of Re, Mo, and V precursors is defined as mole fraction of corresponding precursor (NH_4_ReO_4_ or MoO_2_(C_5_H_7_O_2_)_2_, VO(C_5_H_7_O_2_)_2_). The parenthesis value in X/metal ratio corresponds to the chalcogen (X = S or Se) vacancies (%) calculated using the definition as 1 – ½ [X]/([Re]+[Mo]+[V]).

(a) (ReMoV)S_2_

| No. | Precursors | | | As-grown samples | | | | Annealed samples | | | |
| --- | --- | --- | --- | --- | --- | --- | --- | --- | --- | --- | --- |
|  | Re | Mo | V | Re | Mo | V | S/metal (%) | Re | Mo | V | S/metal (%) |
| ReS_2_ | 1 | 0 | 0 | 1 | 0 | 0 | 2.00 (0) | 1 | 0 | 0 | 1.90 (5) |
| MoS_2_ | 0 | 1 | 0 | 0 | 1 | 0 | 2.49 (0) | 0 | 1 | 0 | 2.20 (0) |
| VS_2_ | 0 | 0 | 1 | 0 | 0 | 1 | 1.69 (16) | 0 | 0 | 1 | 1.42 (29) |
| 1 | 0.42 | 0.42 | 0.16 | 0.48 | 0.36 | 0.15 | 2.21 (0) | 0.49 | 0.35 | 0.16 | 2.14 (0) |
| 2 | 0.25 | 0.50 | 0.25 | 0.29 | 0.46 | 0.25 | 2.27 (0) | 0.29 | 0.46 | 0.25 | 2.16 (0) |
| 3 | 0.50 | 0.25 | 0.25 | 0.54 | 0.22 | 0.24 | 2.25 (0) | 0.51 | 0.23 | 0.26 | 2.16 (0) |
| 4 | 0.33 | 0.33 | 0.33 | 0.38 | 0.29 | 0.33 | 2.19 (0) | 0.38 | 0.29 | 0.33 | 2.13 (0) |
| 5 | 0.16 | 0.42 | 0.42 | 0.20 | 0.37 | 0.43 | 2.21 (0) | 0.19 | 0.38 | 0.42 | 2.06 (0) |
| 6 | 0.42 | 0.16 | 0.42 | 0.44 | 0.13 | 0.43 | 2.10 (0) | 0.43 | 0.14 | 0.42 | 2.00 (0) |
| 7 | 0.25 | 0.25 | 0.50 | 0.28 | 0.22 | 0.50 | 2.06 (0) | 0.27 | 0.23 | 0.50 | 2.02 (0) |
| 8 | 0.165 | 0.165 | 0.67 | 0.20 | 0.14 | 0.66 | 1.95 (2.5) | 0.18 | 0.14 | 0.68 | 1.90 (5) |
| 9 | 0.125 | 0.125 | 0.75 | 0.13 | 0.10 | 0.75 | 1.88 (6) | 0.13 | 0.10 | 0.77 | 1.72 (13) |
| 10 | 0.10 | 0.10 | 0.80 | 0.10 | 0.09 | 0.80 | 1.88 (6) | 0.10 | 0.08 | 0.82 | 1.79 (10) |


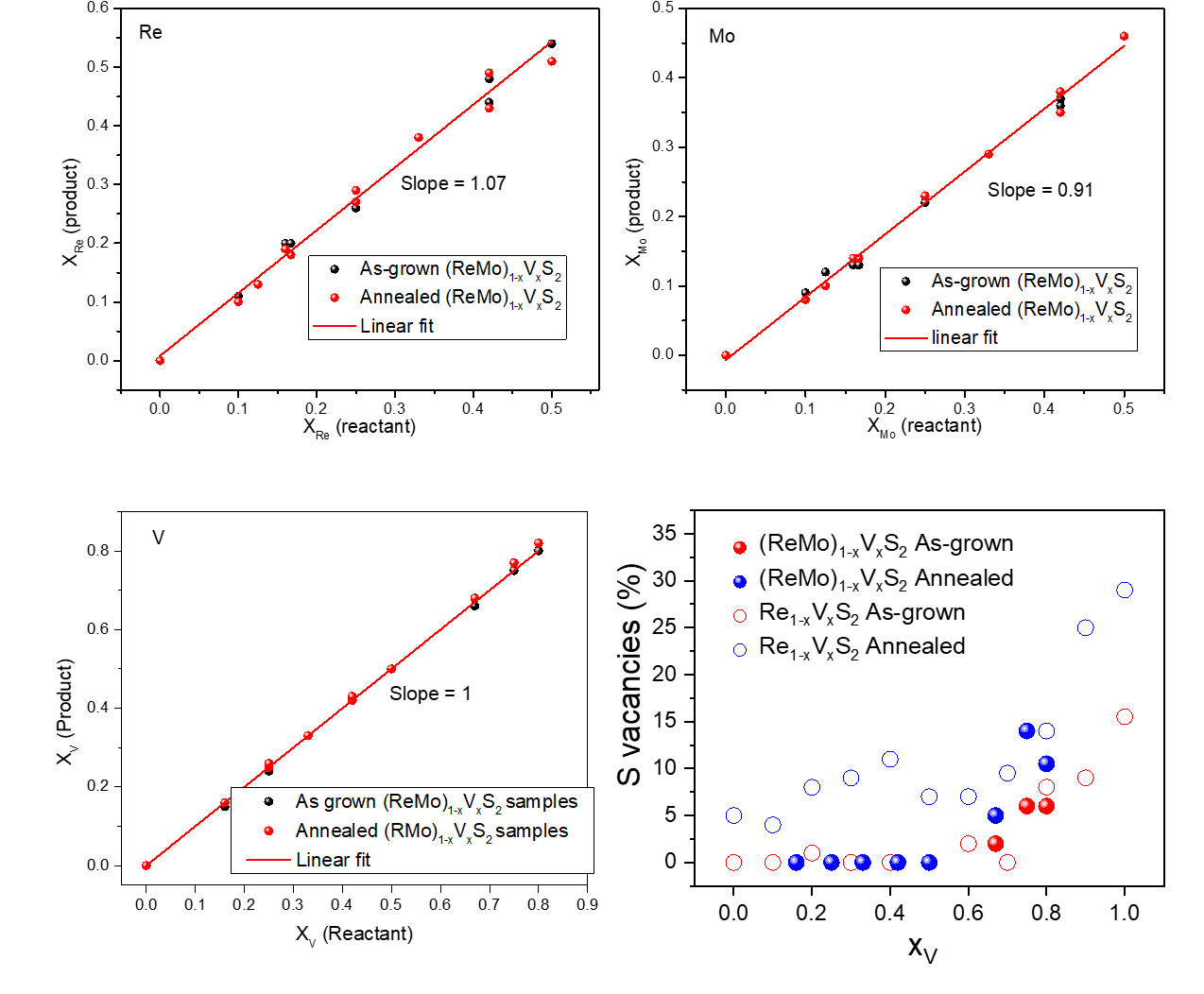


The mole fraction of Re, Mo, and V in the as-grown/annealed samples is correlated with those of precursors. The S vacancies of the (ReMo)_1-_*_x_*V*_x_*S_2_ and Re_1-_*_x_*V*_x_*S_2_ samples are plotted as a function of *x*_V_. The S vacancies increase with *x*_V_. The S vacancies of ternary samples are smaller than those of Re_1-_*_x_*V*_x_*S_2_ binary samples.

(b) (ReMoV)Se_2_

| No. | Precursors | | | Samples | | | |
| --- | --- | --- | --- | --- | --- | --- | --- |
|  | Re | Mo | V | Re | Mo | V | S/metal (%) |
| ReSe_2_ | 1 | 0 | 0 | 1 | 0 | 0 | 2.0 (0) |
| MoSe_2_ | 0 | 1 | 0 | 0 | 1 | 0 | 1.90 (5) |
| VSe_2_ | 0 | 0 | 1 | 0 | 1 | 1 | 1.91 (4.5) |
| 1 | 0.25 | 0.25 | 0.25 | 0.26 | 0.49 | 0.25 | 1.93 (3.5) |
| 2 | 0.50 | 0.25 | 0.25 | 0.50 | 0.25 | 0.25 | 1.90 (5) |
| 3 | 0.33 | 0.33 | 0.33 | 0.43 | 0.29 | 0.28 | 1.88 (6) |
| 4 | 0.25 | 0.25 | 0.50 | 0.28 | 0.30 | 0.42 | 1.91 (4.5) |
| 5 | 0.40 | 0.10 | 0.50 | 0.39 | 0.12 | 0.49 | 1.85 (7.5) |
| 6 | 0.30 | 0.10 | 0.60 | 0.33 | 0.10 | 0.57 | 1.83 (8.5) |
| 7 | 0.167 | 0.167 | 0.67 | 0.17 | 0.19 | 0.64 | 1.87 (6.5) |
| 8 | 0.125 | 0.125 | 0.75 | 0.13 | 0.14 | 0.73 | 1.88 (6) |


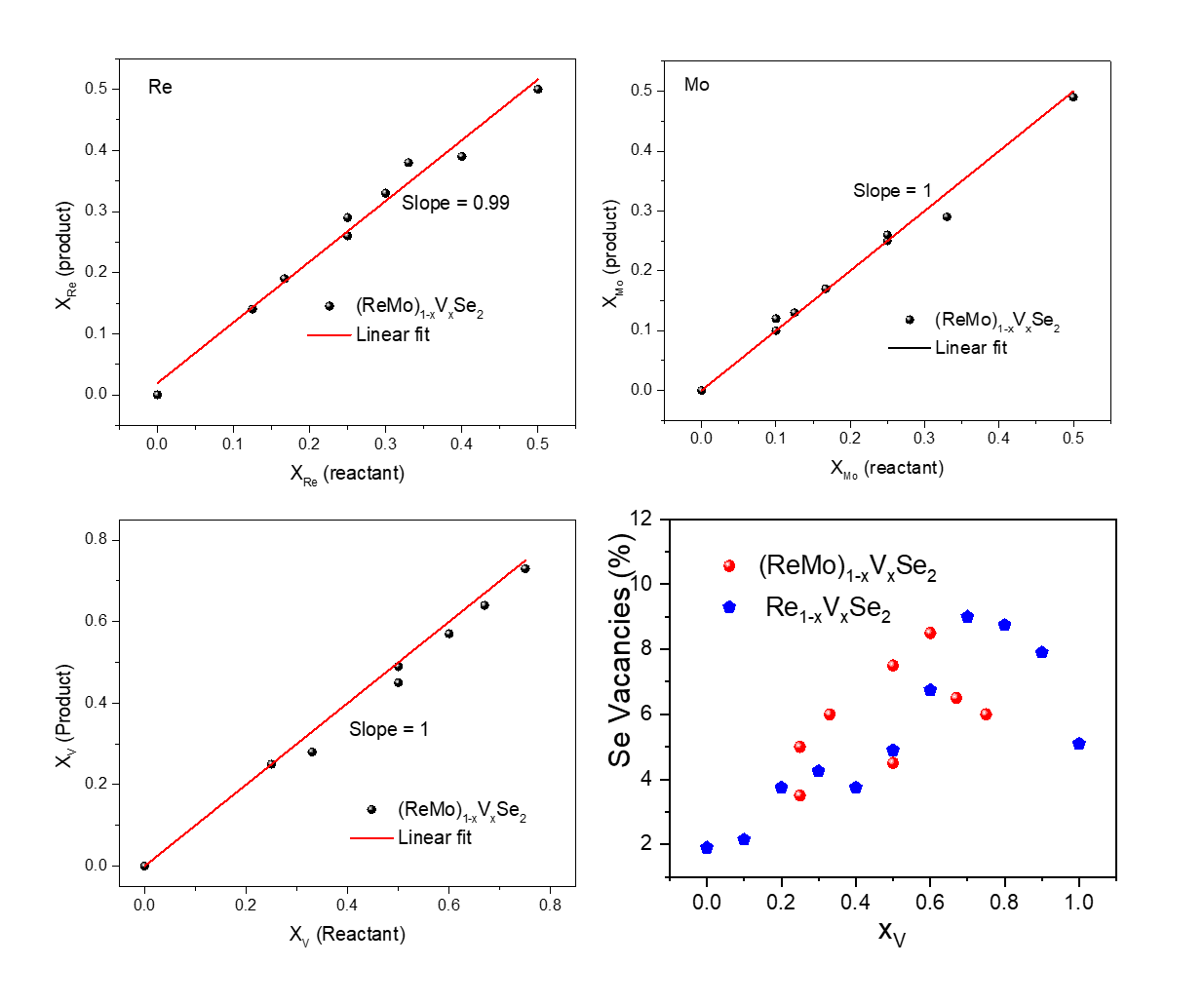


The mole fraction of Re, Mo, and V in the as-grown/annealed samples is well correlated with those of precursors. The Se vacancies of the (ReMo)_1-_*_x_*V*_x_*Se_2_ and Re_1-_*_x_*V*_x_*Se_2_ samples are plotted as a function of *x*_V_. The Se vacancies of alloy samples increase with *x*_V_. The Se vacancies of ternary samples are close to those of binary samples

**Table S2**. Lattice constant and energy of (a) ReX_2_, MoX_2_, VX_2_, (b) 2H- and (c) 1T-like configurations of Re_0.31_Mo_0.38_V_0.31_S_2_ whose structures were built using (4×4×1) supercell. Total energy (E) was for two layers.

(a)

|  | Phase | *a, b*, *c* (Å) | α, β, γ (°) | E_rel_ (eV)*^a^* | E_rel_/atom (meV) |
| --- | --- | --- | --- | --- | --- |
| ReS_2_ | 1T′′ | 12.98, 12.78, 6.43 | 105.10, 91.82, 118.82 |  |  |
| MoS_2_ | 2H | 12.56, 12.56, 12.35 | 90, 90, 120 | 0 | 0 |
|  | 1T′ | 13.13, 12.77, 5.88 | 90, 90, 119 | 17.908 | 186.541 |
| VS_2_ | 1T | 12.84, 12.84, 5.77 | 90, 90, 120 |  |  |
|  | NH_4_- 1T-3R | 13.06, 13.06, 19.08 | 90, 90, 120 |  |  |
| ReSe_2_ | 1T′′ | 13.50, 13.23, 6.70 | 104.98, 91.98, 118.7 |  |  |
| MoSe_2_ | 2H | 13.18, 13.18, 12.99 | 90, 90, 120 | 0 | 0 |
|  | 1T′ | 13.60, 13.06, 12.98 | 90, 90, 119 | 11.867 | 123.615 |
| VSe_2_ | 1T | 13.60, 13.60, 12.30 | 90, 90, 120 |  |  |

*^a^* For E_rel_ = E (1T′) – E (2H), where the total energy (E) of 1T′ MoX_2_ monolayer is multiplied by 2 times.

(b)

| No. | *a* = *b*, *c* (Å) | α, β, γ (°) | E (eV) | E_rel_(eV) | E_rel_/atom (meV) |
| --- | --- | --- | --- | --- | --- |
| 1 | 12.56, 12.35 | 90, 90, 120 | -700.687 | 0 | 0 |
| 2 | 12.56, 12.35 | 90, 90, 120 | -700.667 | 0.019 | 0.200 |
| 3 | 12.55, 12.33 | 90, 90, 120 | -700.553 | 0.134 | 1.395 |
| 4 | 12.56, 12.34 | 90, 90, 120 | -700.331 | 0.355 | 3.701 |
| 5 | 12.56, 12.34 | 90, 90, 120 | -699.961 | 0.726 | 7.560 |
| 6 | 12.56, 12.31 | 90, 90, 120 | -698.797 | 1.889 | 19.678 |
| 7 | 12.56, 12.32 | 90, 90, 120 | -698.739 | 1.947 | 20.284 |
| 8 | 12.57, 12.32 | 90, 90, 120 | -698.527 | 2.160 | 22.500 |
| 9 | 12.57, 12.31 | 90, 90, 120 | -697.875 | 2.811 | 29.283 |

(c)

| No. | *a*, *b*, *c* (Å) | α, β, γ (°) | E (eV) | E×2 (eV) | E_rel_(eV) | E_rel_/atom  (meV) |
| --- | --- | --- | --- | --- | --- | --- |
| 1 | 12.91, 12.97, 5.93 | 89.72, 90.66, 120.18 | -344.152 | -688.305 | 0 | 0 |
| 2 | 12.96, 12.94, 5.88 | 89.11, 90.32, 120.24 | -344.146 | -688.292 | 0.013 | 0.134 |
| 3 | 13.01, 12.90, 5.84 | 90.73, 89.36, 119.97 | -344.103 | -688.205 | 0.099 | 1.035 |
| 4 | 13.02, 12.76, 5.97 | 90.10, 88.83, 119.31 | -344.056 | -688.112 | 0.193 | 2.009 |
| 5 | 12.97, 12.74, 6.35 | 105.09, 91.65, 119.44 | -343.843 | -687.687 | 0.618 | 6.435 |
| 6 | 13.03, 12.92, 5.91 | 90.36, 89.03, 119.95 | -343.828 | -687.657 | 0.648 | 6.751 |
| 7 | 12.95, 12.74, 6.33 | 104.66, 91.80, 119.37 | -343.776 | -687.553 | 0.752 | 7.831 |
| 8 | 12.93, 12.77, 6.34 | 104.77, 91.08, 119.62 | -343.643 | -687.285 | 1.019 | 10.619 |
| 9 | 12.96, 12.57, 6.35 | 104.26, 93.79, 119.00 | -343.619 | -687.239 | 1.066 | 11.101 |

**Table S3**. Fitting parameters of EXAFS data (**Figure S6**) for (a) as-grown (ReMoV)S_2_ samples and *ex situ* sample **8**(S); *^a^*Distance between scattering atoms, *^b^*Coordination number. *^c^*Edge energy shift, representing between the energy grids of experimental and theoretical data. *^d^*Debye-Waller factor, which measure the static and thermal disorder.

1. As-grown (ReMoV)S_2_ samples;

Re L_3_ edge

| No. | *x*_V_ | Scattering path | R(Å)*^a^* | CN*^b^* | ΔE(eV)*^c^* | σ^2^ (Å^2^)*^d^* |
| --- | --- | --- | --- | --- | --- | --- |
| ReS_2_ | 0 | Re-S | 2.386 | 5.9 ± 0.65 | 3.76 | 0.007 |
| 1 | 0.16 | Re-S | 2.367 | 5.1 ± 0.6 | 6.8 | 0.006 |
| 4 | 0.33 | Re-S | 2.375 | 5.2 ± 0.9 | 7.4 | 0.005 |
| 7 | 0.5 | Re-S | 2.385 | 5.7 ± 0.6 | 7.5 | 0.006 |
| 8 | 0.67 | Re-S | 2.382 | 5.7 ± 0.6 | 7.8 | 0.005 |
| 10 | 0.8 | Re-S | 2.375 | 6.0 ± 0.6 | 6.4 | 0.006 |

Mo K edge

| No. | *x*_V_ | Scattering path | R(Å) | CN | ΔE(eV) | σ^2^ (Å^2^) |
| --- | --- | --- | --- | --- | --- | --- |
| MoS_2_ | 0 | Mo-S | 2.414 | 6.0 ± 0.8 | 0.082 | 0.007 |
| 1 | 0.16 | Mo-S | 2.408 | 5.9 ± 0.6 | 0.50 | 0.006 |
| 4 | 0.33 | Mo-S | 2.406 | 5.7 ± 1.0 | 0.81 | 0.007 |
| 7 | 0.5 | Mo-S | 2.410 | 5.9 ± 0.7 | 1.84 | 0.007 |
| 8 | 0.67 | Mo-S | 2.413 | 5.6 ± 0.7 | 1.82 | 0.006 |
| 10 | 0.8 | Mo-S | 2.416 | 5.6 ± 0.6 | 0.97 | 0.006 |

V K edge

| No. | *x*_V_ | Scattering path | R(Å) | CN | ΔE(eV) | σ^2^ (Å^2^) |
| --- | --- | --- | --- | --- | --- | --- |
| VS_2_ | 1 | V-S | 2.294 | 3.0 ± 0.3 | 1.44 | 0.008 |
| 4 | 0.33 | V-S | 2.330 | 4.5 ± 1.1 | -2.83 | 0.008 |
| 7 | 0.50 | V-S | 2.317 | 3.5 ± 0.3 | -3.28 | 0.006 |
| 8 | 0.75 | V-S | 2.326 | 3.0 ± 0.4 | -1.9 | 0.006 |
| 10 | 0.8 | V-S | 2.332 | 4.1 ± 1.2 | -2.11 | 0.011 |

(b) *Ex-situ* sample **8**(S)

Re L_3_ edge

| η (mV) | Scattering path | R(Å)*^a^* | CN*^b^* | ΔE(eV)*^c^* | σ^2^ (Å^2^)*^d^* |
| --- | --- | --- | --- | --- | --- |
| 0 | Re-S | 2.379 | 5.8 +/- 0.5 | 7.2 | 0.006 |
| 20 | Re-S | 2.373 | 5.6 +/- 0.4 | 7.4 | 0.006 |
| 50 | Re-S | 2.386 | 5.6 +/- 0.6 | 8.2 | 0.006 |
| 100 | Re-S | 2.381 | 5.9 +/- 0.6 | 7.5 | 0.007 |

Mo L_3_ edge

| η (mV) | Scattering path | R(Å)*^a^* | CN*^b^* | ΔE(eV)*^c^* | σ^2^ (Å^2^)*^d^* |
| --- | --- | --- | --- | --- | --- |
| 0 | Mo-S | 2.413 | 5.5 +/- 0.8 | 1.1 | 0.007 |
| 20 | Mo-S | 2.414 | 5.0 +/- 0.9 | 0.8 | 0.006 |
| 50 | Mo-S | 2.419 | 5.1 +/- 0.9 | 2.7 | 0.007 |
| 100 | Mo-S | 2.409 | 4.8 +/- 0.6 | 1.5 | 0.004 |

V K edge

| η (mV) | Scattering path | R(Å)*^a^* | CN*^b^* | ΔE(eV)*^c^* | σ^2^ (Å^2^)*^d^* |
| --- | --- | --- | --- | --- | --- |
| 0 | V-S | 2.317 | 4.3 +/- 0.4 | -3.4 | 0.008 |
| 20 | V-S | 2.320 | 4.5 +/- 0.5 | -2.7 | 0.010 |
| 50 | V-S | 2.322 | 4.5 +/- 0.5 | -3.2 | 0.009 |
| 100 | V-S | 2.321 | 4.7 +/- 0.5 | -2.8 | 0.011 |

**Table S4**. HER performance of (a) as-grown and (b) annealed (ReMoV)S_2_, and (c) (ReMoV)Se_2_ samples. *^a^* Overpotential (mV vs. RHE) at *J* = 10 mA cm^-2^; *^b^* Tafel slope for HER; *^c^* Charge transfer resistance obtained using Nyquist plot of EIS data; *^d^* Double layer capacitance; *^e^* Overpotential (mV vs. RHE) at *J_ECSA_* = 0.05 mA cm^-2^.

(a) as-grown (ReMoV)S_2_,

| Sample No. | η_J=10_ (mV)*^a^* | *b* (mV dec^-1^)*^b^* | *R_ct_* (Ω)*^c^* | *C_dl_* (mF cm^-2^)*^d^* | η_JECSA=0.05_ (mV)*^e^* |
| --- | --- | --- | --- | --- | --- |
| ReS_2_ | 144 | 103 | 26.30 | 28.63 | 187 |
| MoS_2_ | 172 | 54 | 39.22 | 44.03 | 200 |
| VS_2_ | 161 | 77 | 22.84 | 6.17 | 140 |
| 1 | 139 | 76 | 17.95 | 27.87 | 168 |
| 2 | 138 | 66 | 15.15 | 35.14 | 174 |
| 3 | 125 | 63 | 10.77 | 39.68 | 144 |
| 4 | 117 | 58 | 7.79 | 45.74 | 151 |
| 5 | 132 | 59 | 12.37 | 49.46 | 172 |
| 6 | 124 | 57 | 9.47 | 49.67 | 160 |
| 7 | 116 | 55 | 7.16 | 47.82 | 144 |
| 8 | 104 | 55 | 5.60 | 61.31 | 143 |
| 9 | 107 | 54 | 5.23 | 52.96 | 140 |
| 10 | 122 | 53 | 7.80 | 38.22 | 149 |

(b) annealed (ReMoV)S_2_,

| Sample No. | η_J=10_ (mV)*^a^* | *b* (mV dec^-1^)*^b^* | *R_ct_* (Ω)*^c^* | *C_dl_* (mF cm^-2^)*^d^* | η_JECSA=0.05_ (mV)*^e^* |
| --- | --- | --- | --- | --- | --- |
| ReS_2_ | 172 | 104 | 48.64 | 18.00 | 193 |
| MoS_2_ | 187 | 80 | 127.93 | 20.58 | 199 |
| VS_2_ | 164 | 80 | 70.37 | 11.40 | 182 |
| 1 | 214 | 97 | 102.9 | 6.59 | 190 |
| 2 | 207 | 90 | 90.43 | 6.81 | 185 |
| 3 | 153 | 66 | 19.74 | 21.44 | 169 |
| 4 | 158 | 73 | 21.54 | 22.54 | 177 |
| 5 | 177 | 93 | 47.27 | 20.00 | 197 |
| 6 | 147 | 80 | 22.37 | 39.42 | 192 |
| 7 | 153 | 77 | 23.20 | 24.77 | 178 |
| 8 | 123 | 86 | 15.54 | 55.78 | 184 |
| 9 | 129 | 77 | 14.37 | 58.52 | 184 |
| 10 | 156 | 67 | 21.02 | 19.49 | 170 |

(c) (ReMoV)Se_2_

| Sample No. | η_J=10_ (mV)*^a^* | *b* (mV dec^-1^)*^b^* | *R_ct_* (Ω)*^c^* | *C_dl_* (mF cm^-2^)*^d^* | η_JECSA=0.1_ (mV)*^e^* |
| --- | --- | --- | --- | --- | --- |
| ReSe_2_ | 141 | 67 | 11.26 | 3.88 | 125 |
| MoSe_2_ | 131 | 73 | 18.27 | 8.06 | 143 |
| VSe_2_ | 320 | 103 | 28422 | 1.86 | 260 |
| 1 | 135 | 67 | 102.9 | 15.40 | 162 |
| 2 | 124 | 68 | 90.43 | 12.74 | 145 |
| 3 | 110 | 63 | 19.74 | 7.60 | 139 |
| 4 | 127 | 61 | 21.54 | 10.98 | 152 |
| 5 | 121 | 61 | 47.27 | 10.73 | 140 |
| 6 | 121 | 61 | 22.37 | 8.87 | 142 |
| 7 | 128 | 69 | 23.20 | 13.76 | 143 |
| 8 | 135 | 70 | 21.02 | 15.20 | 156 |

**Table S5**. Comparison of HER performance (in 0.5 M H_2_SO_4_ electrolyte) of ReS_2_-based and alloy TMDs in the literatures.

| Ref. No. | Materials | η_J=10_/η_J=100_ (mV) | Tafel slope  (mV dec^-1^) |
| --- | --- | --- | --- |
| S10 | Exfoliated ReS_2_ | 260 | 75 |
| S11 | Hydrothermal ReS_2_ /carbon fiber paper | 116 (light) | 137 |
| S12 | Hydrothermal ReS_2_/carbon fiber | 220 (dark)  167 (light) | 80 (dark)  77 (light) |
| S13 | CVD-grown Re-vacant ReS_2_/Si | 147 | 69 |
| S14 | Exfoliated ReS_2_/carbon fiber | 173 | 69.5 |
| S15 | NiFe LDH-hydrothermal ReS_2_ | 101 | 81 (1M KOH) |
| S16 | Colloidal V(2.3%):Co(6.7%)-doped ReS_2_ | 31 | -- |
| S17 | Wet-chemically grown WS_2_  MoS_2_  W_0.4_Mo_0.6_S_2_ | Onset: 150  197  96 | 68.4  82  50.6 |
| S18 | MoO_3_-selenized W_0.13_Mo_0.87_Se_2_ on CVT | 129 | 53 |
| S19 | Exfoliated ReS_2_  ReSe_2_  ReSSe | 320  123  84 | 106.9  50.8  50.1 |
| S20 | CVD-grown ReS_2_  1T MoS_2_  Re_0.55_Mo_0.45_S_2_ | ~250  ~200  169 | ~200  ~70  56 |
| S21 | Exfoliated VS_2_  VSe_2_  VSSe | 561  781  180 | 136  174  87 |
| S22 | Hydrothermal ReS_2_  MoS_2_  Re_0.5_Mo_0.5_S_2_ | 211  187  98 | 108  80  54 |
| S23 | Hydrothermal ReSe_2_  MoSe_2_  Re_0.9_Mo_0.1_Se_2_ | 107  188  77 | 61  77  56 |
| S24 | Colloidal MoSe_2_  VSe_2_  Mo_0.7_V_0.3_Se_2_ | 195  330  114 | 54  76  43 |
| S25 | Colloidal WSe_2_  VSe_2_  W_0.7_V_0.3_Se_2_ | 168  387  128 | 83  107  80 |
| S26 | Solid-state reaction ReSe_2_  WSe_2_  Re_0.7_W_0.3_Se_2_ | 203  ~350  141 | 72.7  128  65.3 |
| S27 | CVD-grown ReS_2_  MoS_2_  1T’-Re_1-x_Mo_x_S_2_/2H-MoS_2_ | ~300  ~200  84 | 128  142  58 |
| S28 | Colloidal WSe_2_  ReSe_2_  W_0.51_Re_0.49_Se_2_ | ~400  ~360  ~160 | ~150  ~125  44 |
| S29 | Colloidal ReSe_2_  VSe_2_  Re_0.8_V_0.2_Se_2_ | 141  320  77 | 67  103  62 |
| S30 | Solvothermal ReS_2_  VS_2_  Re_0.2_V_0.8_S_2_ | 144  117  161 | 103  54  77 |
| This work | ReS_2_  MoS_2_  VS_2_  Re_0.165_ Mo_0.165_V_0.67_S_2_ | 144  172  161  104 | 103  54  77  55 |

(a)


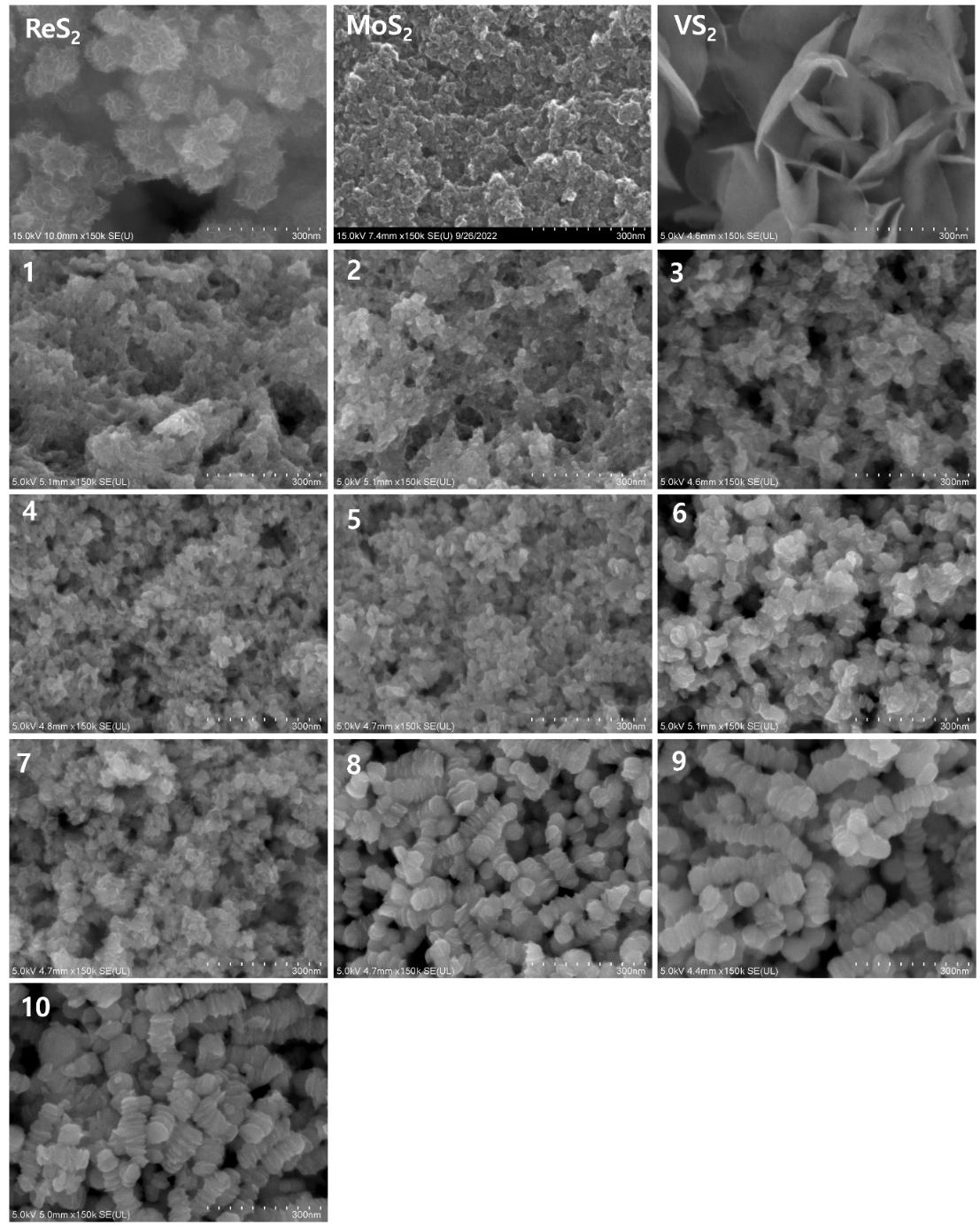


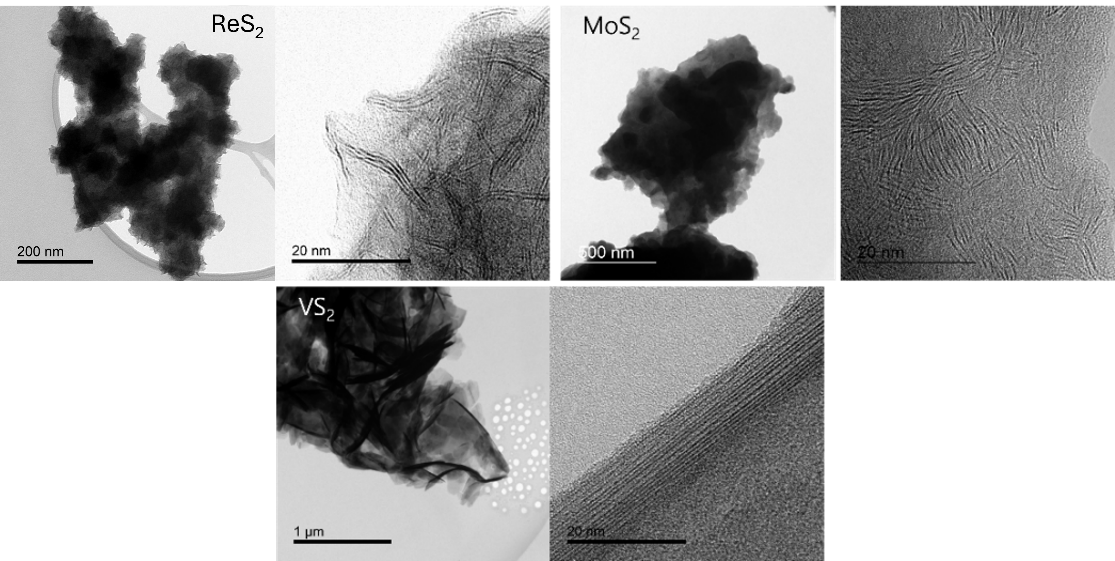


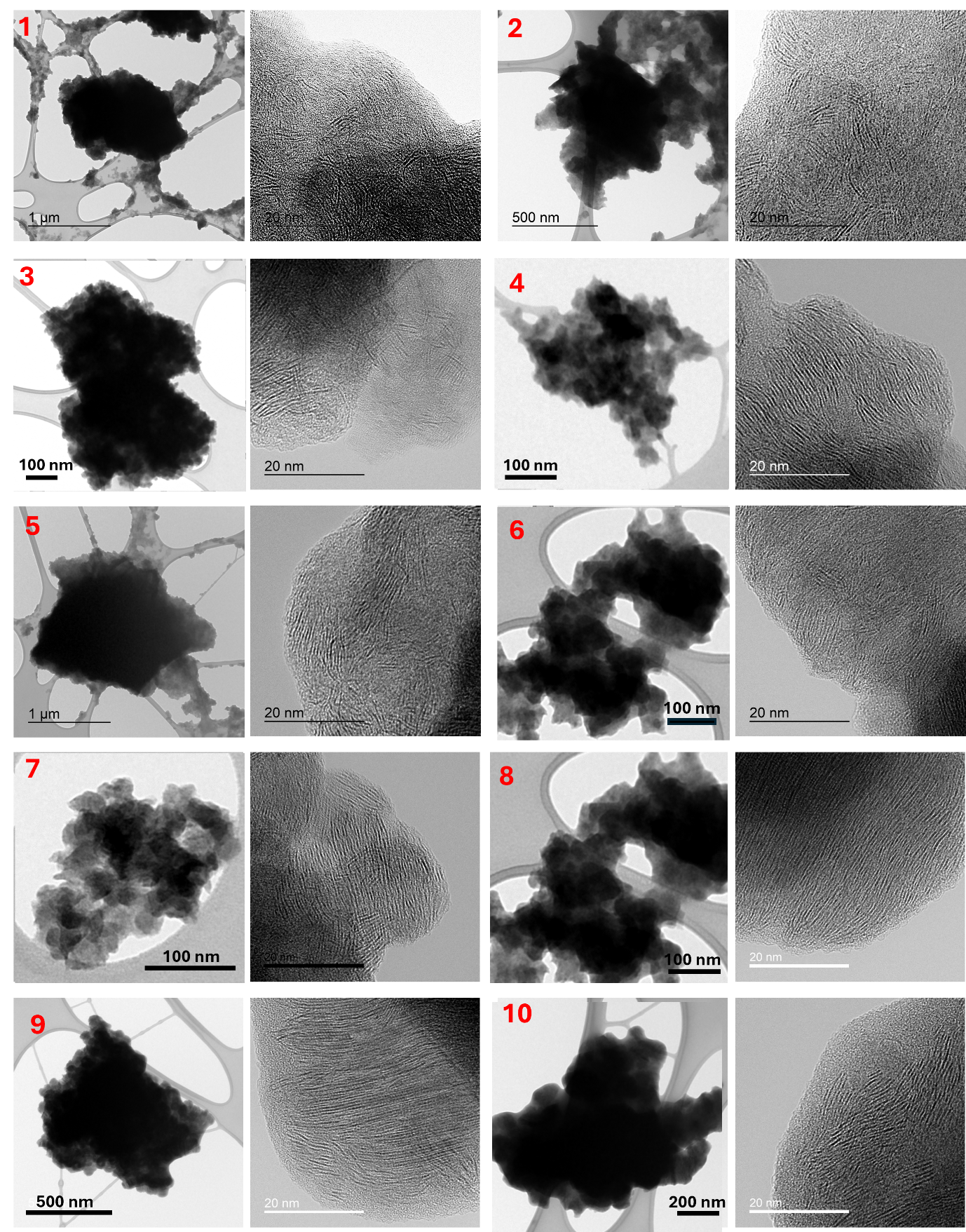


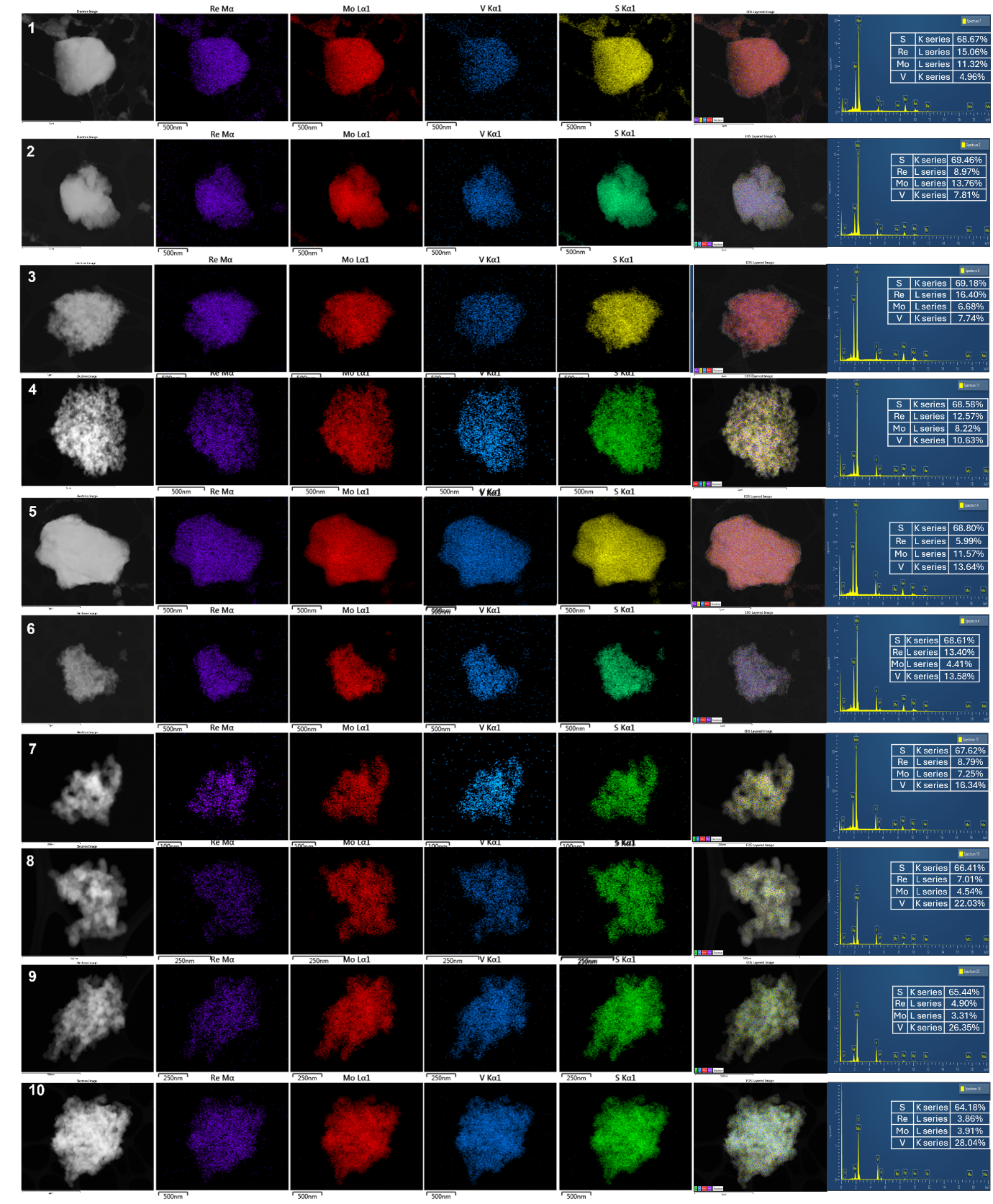


(b)


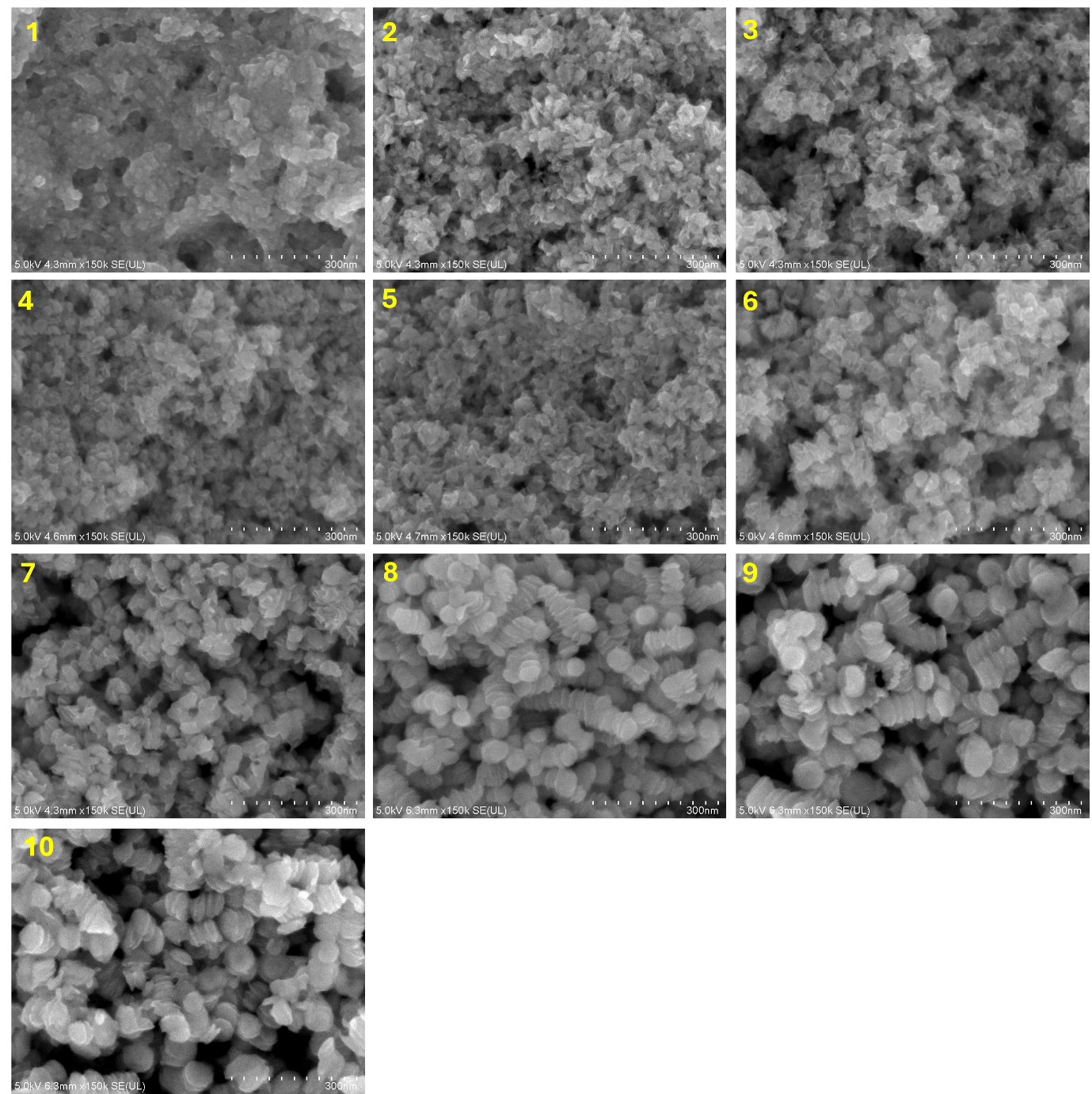


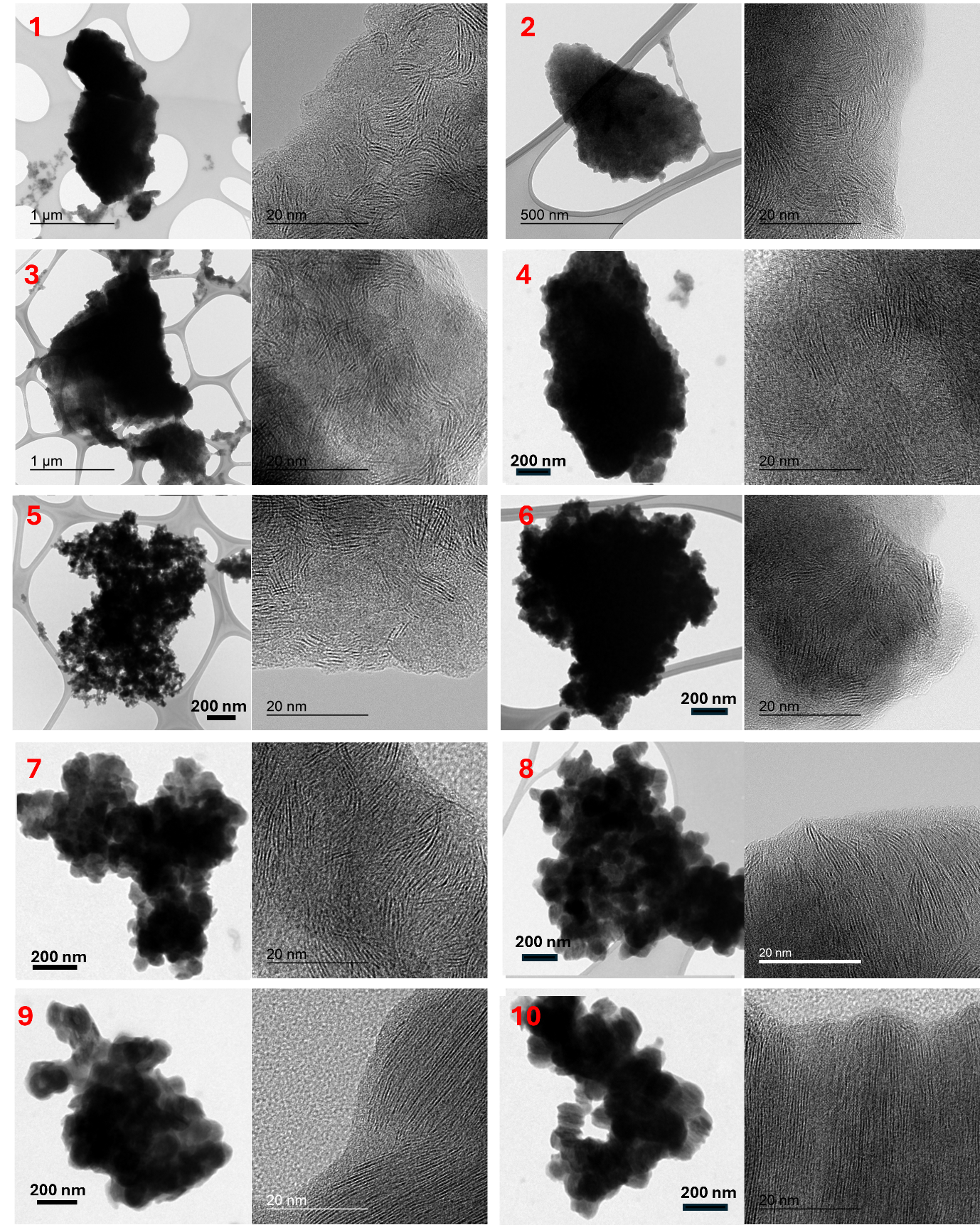


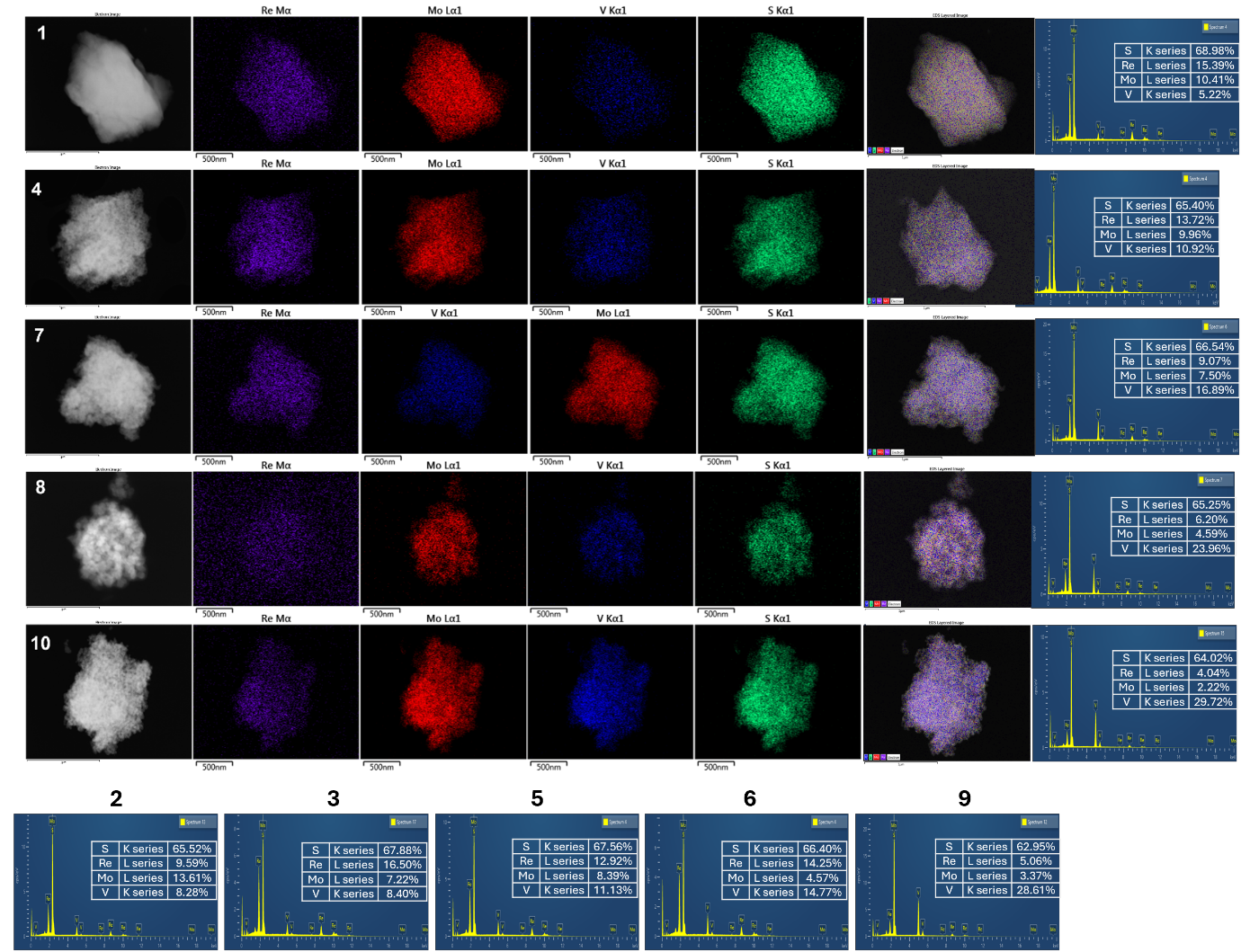
**Figure S1**. SEM, TEM, and EDX data of (a) as-grown and (b) annealed (ReMoV)S_2_ samples. HAADF STEM images and EDX elemental mapping, and corresponding spectrum. The Re (M shell), Mo (L shell), V (K shell), and S (K shell) distributed homogeneously in entire nanosheets. The metal composition was successfully controlled.

The annealing didn’t change the morphology and metal composition. The ReS_2_ nanosheets comprised 2–5 layers (thickness: ~2 nm) that aggregated into nanoflowers with a size of ~100 nm. The MoS_2_ nanosheets (thickness: 2–5 nm) aggregated to form random-sized nanoflowers (30–50 nm). The thickness and size of the VS_2_ nanosheets were approximately 10 and 200 nm, respectively. After ternary alloying, the size of the nanoflowers decreased to less than 50 nm. The interlayer distance (*d*_001_) of as-grown nanosheets was ~10 Å. After annealing, *d*_001_ decreased to 6 Å. As-grown **1**–**7** samples show [S]/[Metal] ≥ 2 (no S vacancies), and **8**–**10** samples show [S]/[Metal] = 1.95, 1.88, and 1.88 (corresponding to C_VS_ = 2.5, 6, and 6%), respectively. Annealed **1**–**7** samples have no S vacancy, and **8**–**10** samples show [S]/[Metal] = 1.90, 1.72, and 1.79 (corresponding to C_VS_ = 5, 13, and 10%), respectively (see Table S1).


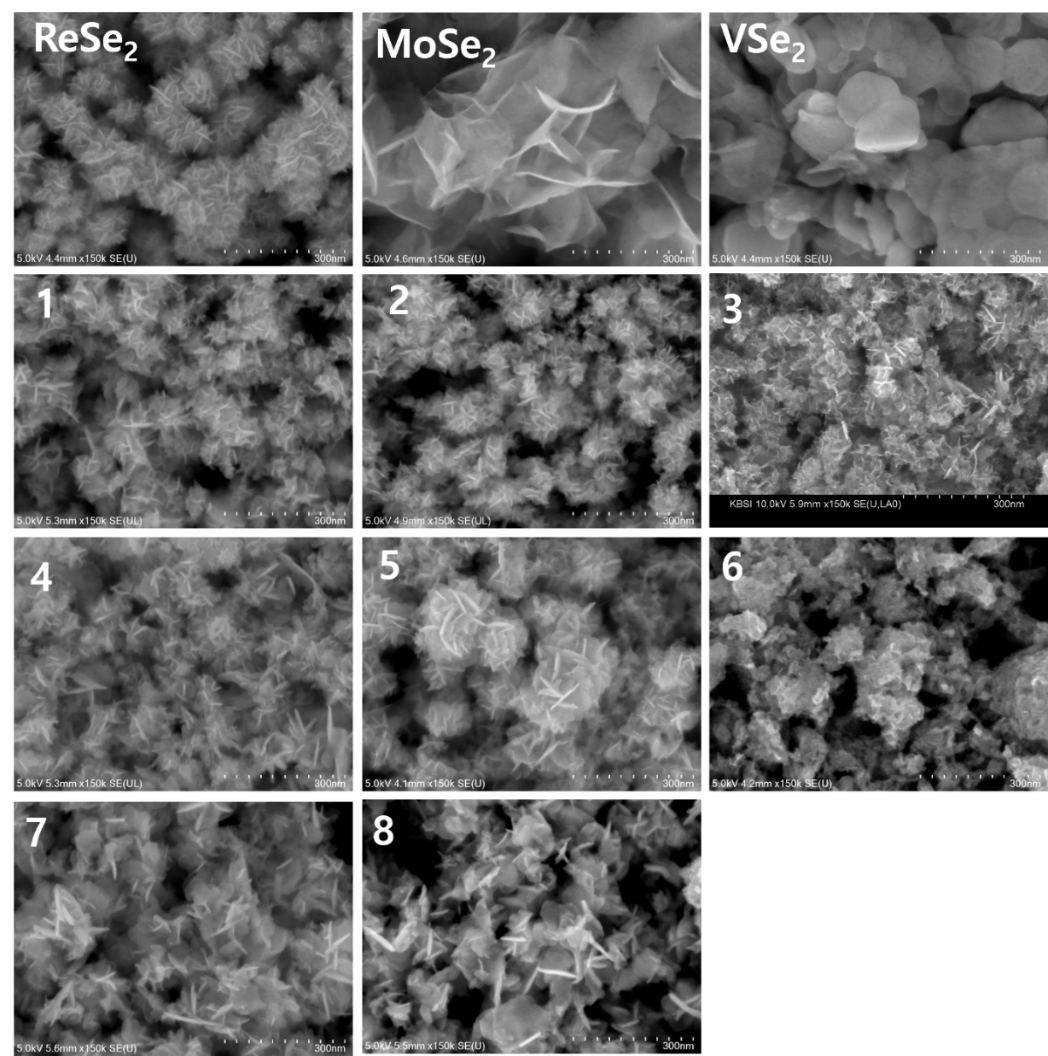

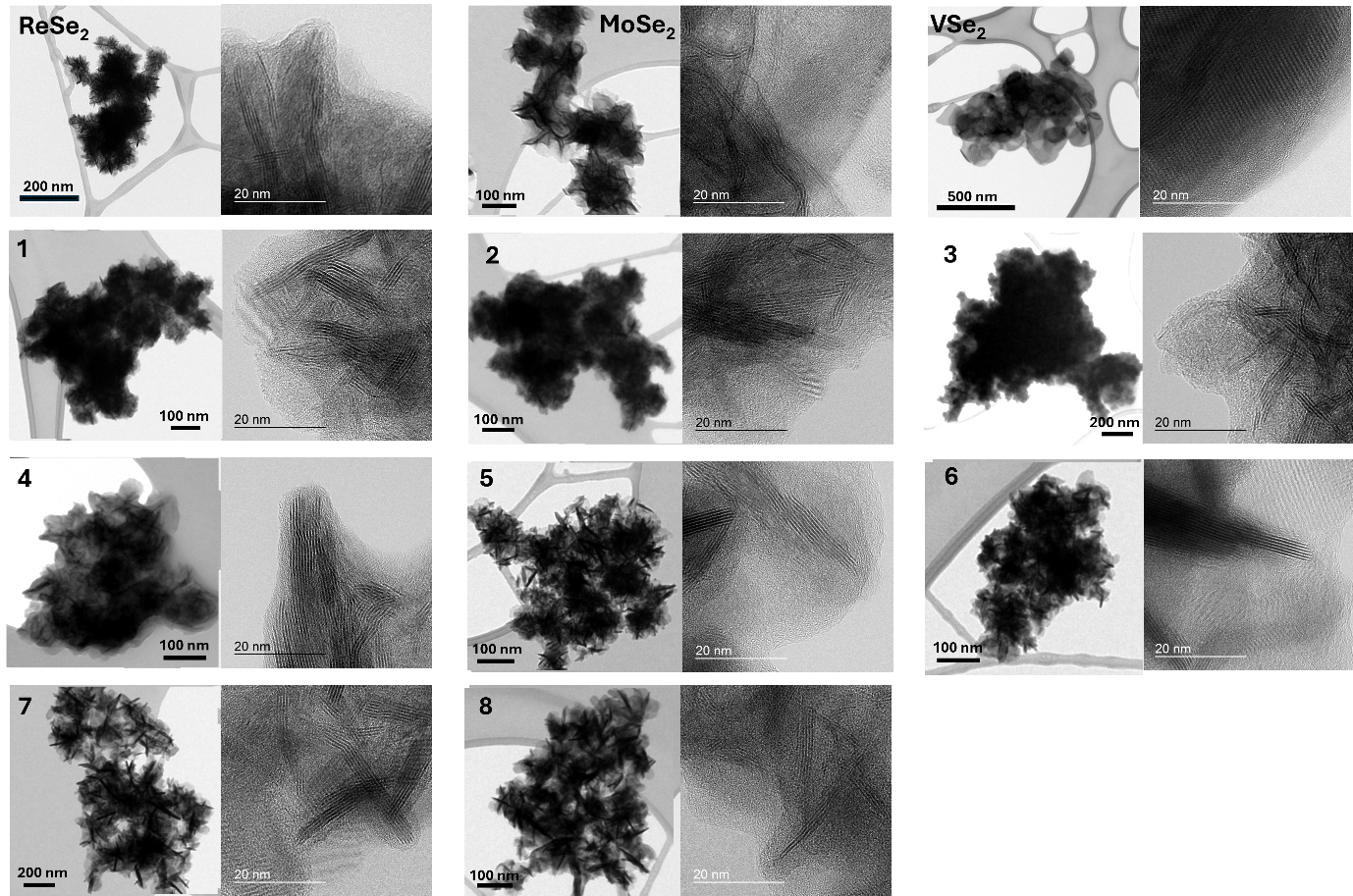

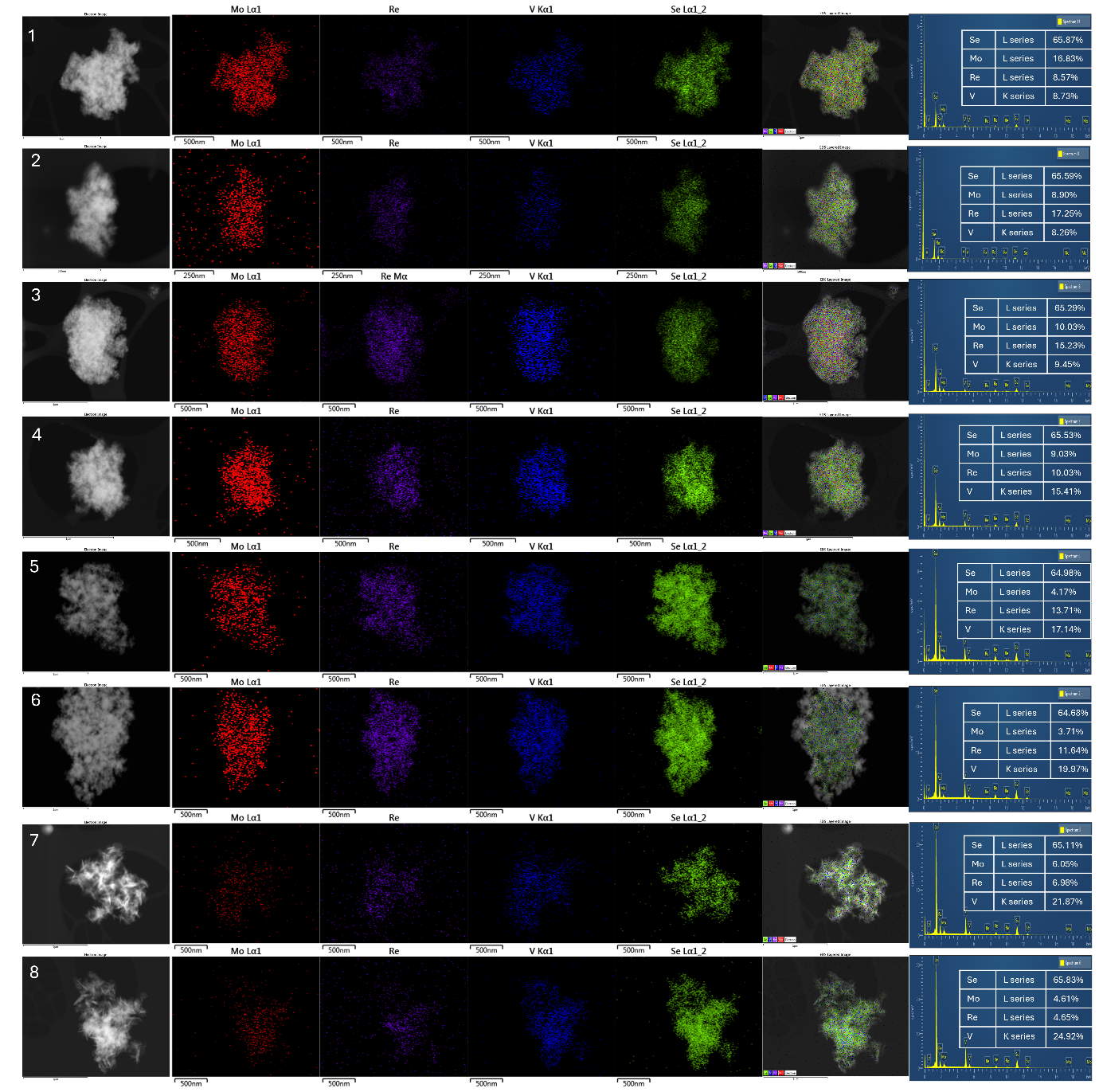
 **Figure S2**. SEM, HRTEM, and EDX data (HAADF STEM images, elemental mapping, and spectrum) of (ReMoV)Se_2_ samples. The Re (M shell), Mo (L shell), V (K shell), and Se (L shell) distributed homogeneously in entire nanosheets. The metal composition was successfully controlled. The ReSe_2_ nanosheets comprised 2–5 layers (thickness: ~2 nm) that aggregated into nanoflowers with a size of ~100 nm. By contrast, the thickness and size of the VSe_2_ nanosheets were approximately 10 and 200 nm, respectively. The MoSe_2_ nanosheets had a thickness of ~2 nm and were bundled into nanoflowers ~100 nm in size. After alloying, the size of the nanoflowers decreased to less than 50 nm. The interlayer distance (*d*_001_) was ~6 Å. The samples show [S]/[Metal] = 1.9 (to C_VSe_ = 5%) in average.

(a)


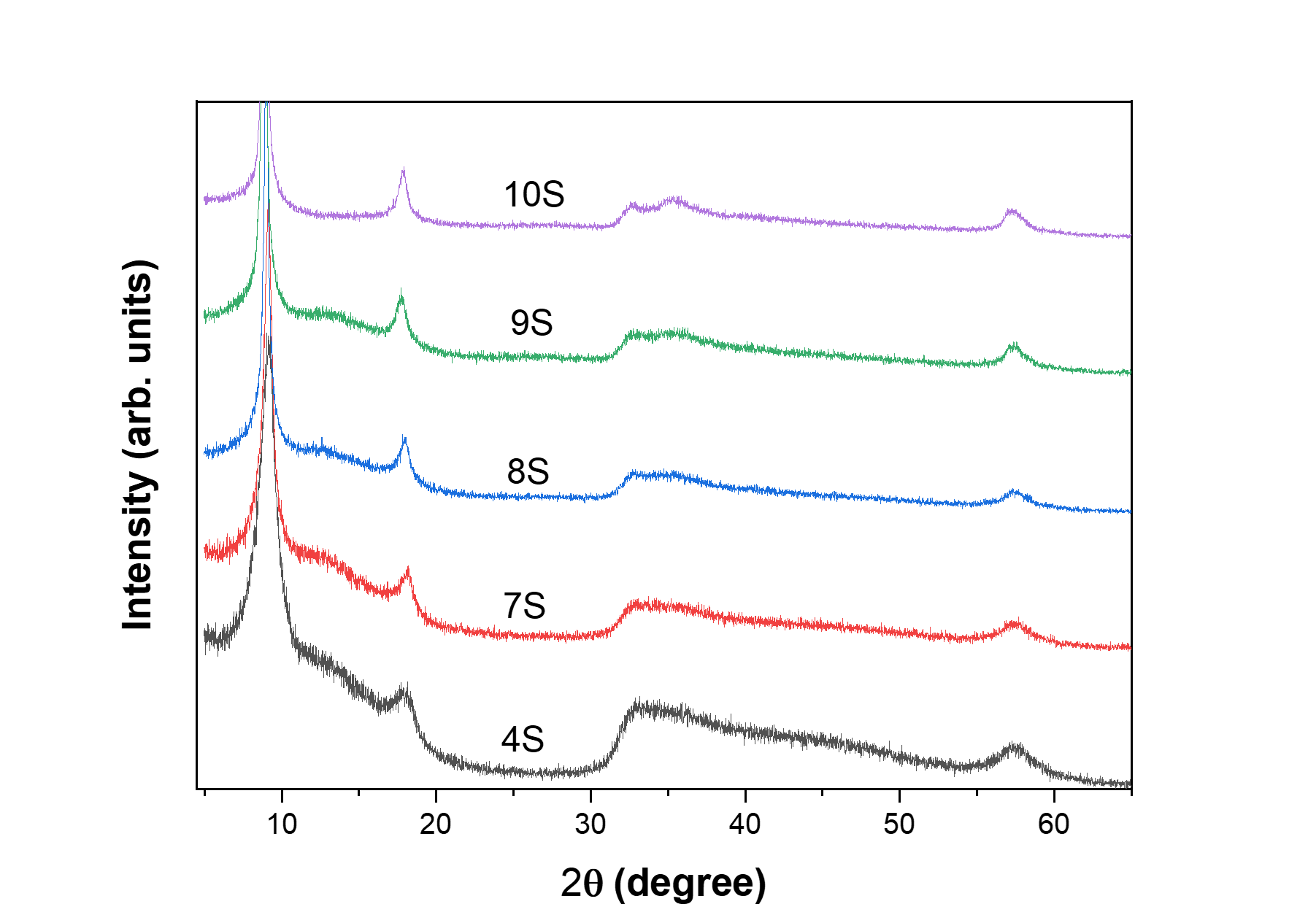


(b)


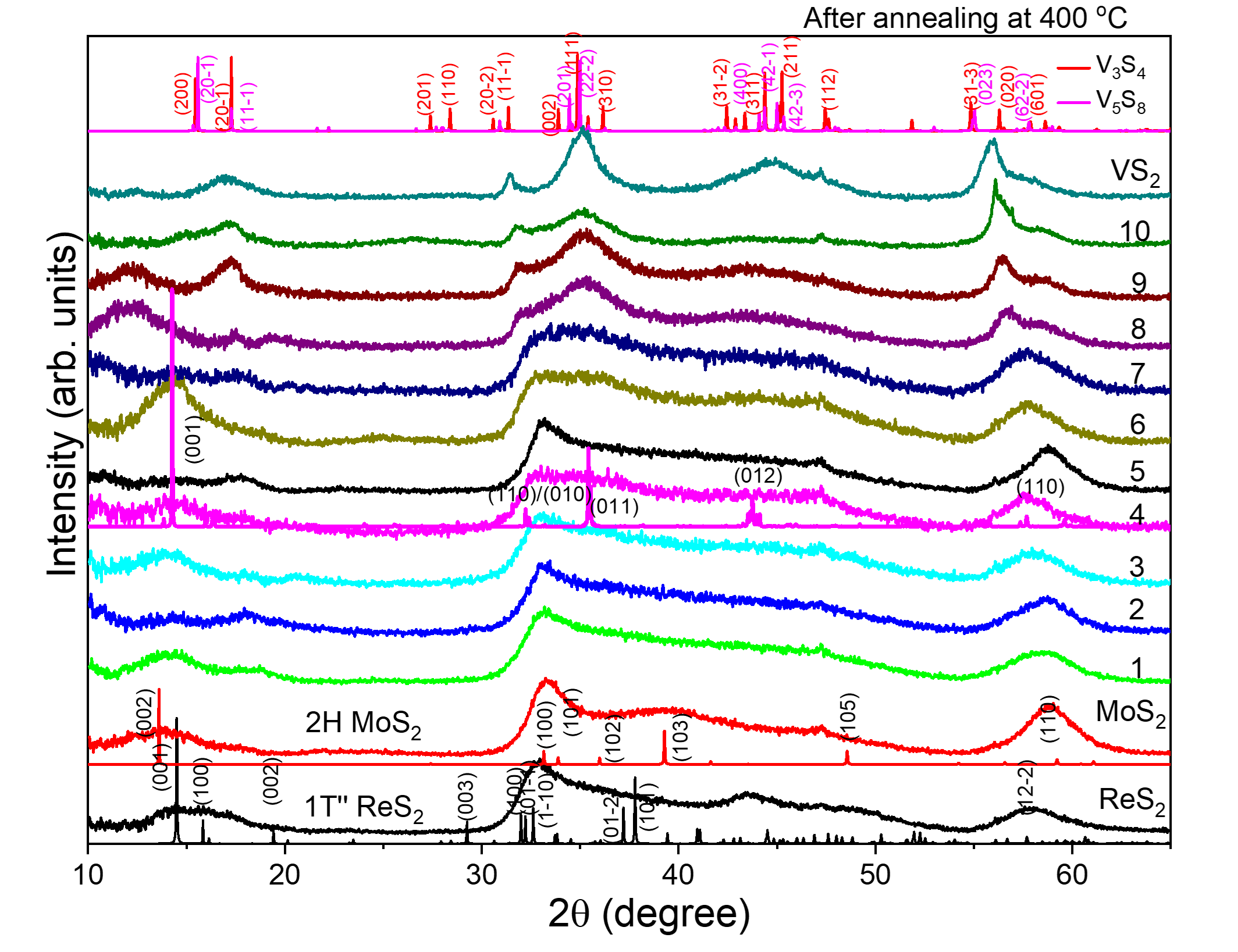


**Figure S3**. (a) XRD data of as-grown ternary alloy samples to prove the repeatability of XRD shown in Figure 2a. (b) XRD data of annealed ReS_2_, MoS_2_, VS_2_, and their ternary alloy samples. The XRD peaks of annealed ReS_2_ are matched to the calculated lattice constants; *a* = 6.49 Å, *b* = 6.39 Å, *c* = 6.43 Å, α = 105.10°, β = 91.82°, and γ = 118.82°. The lattice constant is close to the reference value (JCPDS No. 24-0922; P, *a* = 6.455 Å, *b* = 6.362 Å, *c* = 6.401 Å, α = 105.04°, β = 91.60°, and γ = 118.97°). The annealed MoS_2_ has the lattice constant of 2H phase (*a* = 3.165 Å and *c* = 12.35 Å), which is close to the reference value (JCPDS No. 87-2416; *a* = 3.160 Å and *c* = 12.290 Å). The VS_2_ samples show the XRD peaks of monoclinic V_3_S_4_ phase (C2/m, *a* = 6.7556 Å, *b* = 3.26585 Å, *c* = 5.84651 Å, β = 115.2984°) and/or V_5_S_8_ (F2/m, *a* = 11.36951 Å, *b* = 6.68002 Å, *c* = 7.99487 Å, β = 133.7836°). The *d*_L_ (interlayer distance) value is 5.286 and 5.772 Å, respectively. The 3R-1T phase VS_2_ underwent the phase change into V_3_S_4_ and/or V_5_S_8_ upon annealing. The peaks of V_3_S_4_/V_5_S_8_ appears from the samples **6** and **7**. Therefore, the phase transition to V_3_S_4_/V_5_S_8_ occurs at *x*_V_ = 0.42-0.5, consistently with the as-grown samples.

For the sample **4**, we plotted the calculated XRD pattern using the lattice constants of 1T-like structure with the lattice constants of (*a*, *b*, *c*) = (12.91, 12.97, 6.2) Å and (*α*, *β*, *γ*) = (89.72°, 90.66°, 120.18°). The experimental XRD peaks fit well the calculated ones, indicating that the ternary samples have the 1T-like structure.


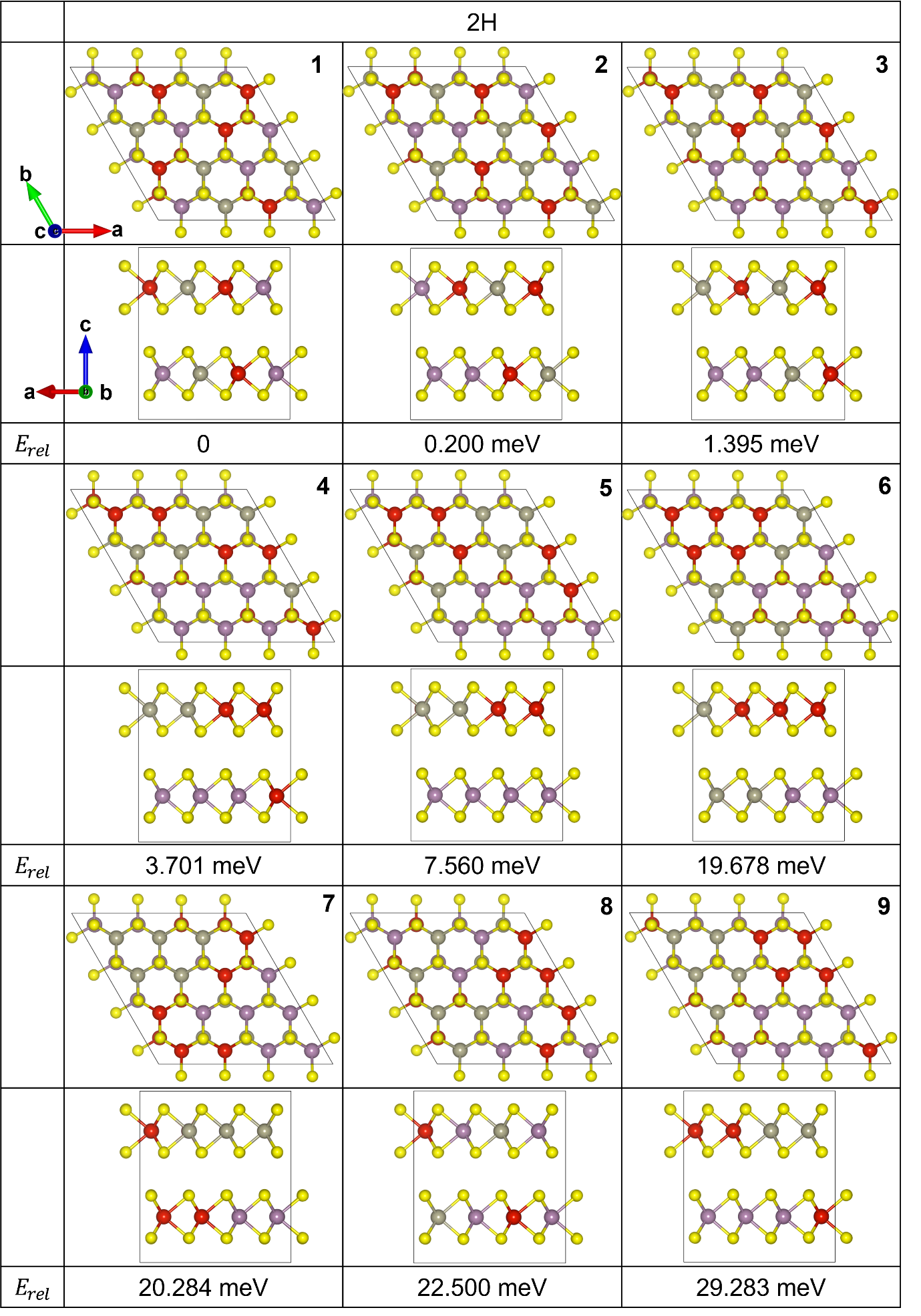


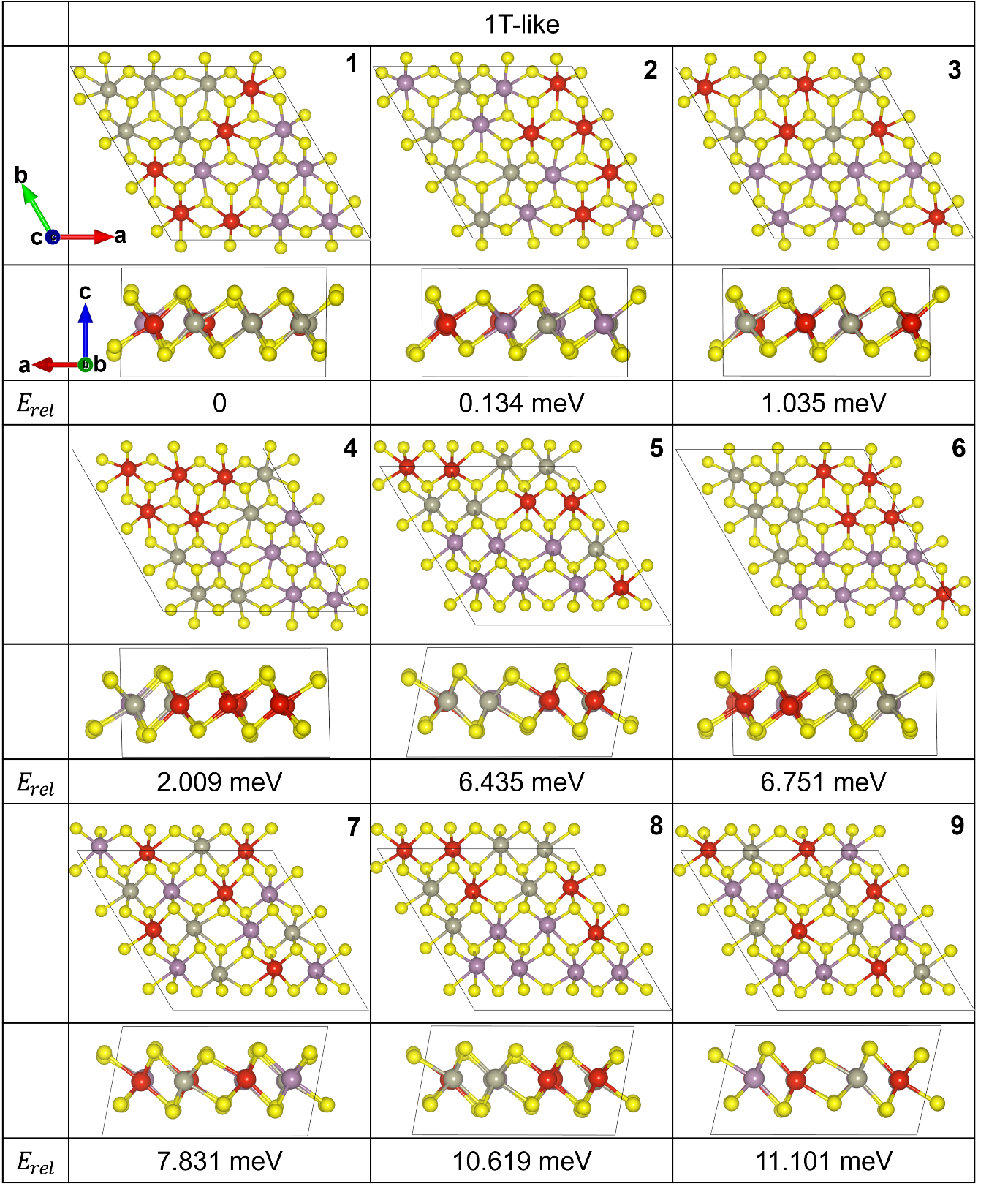


**Figure S4**. Configurations for 2H and 1T-like phase of Re_0.31_Mo_0.38_V_0.31_S_2_, projected along the basal plane (*c* axis, top) and *b*-axis (bottom) and their relative energy (E_rel_). They were built from (4 × 4 × 1) supercell. The number of each configuration is matched to that of **Table S2**. The relative energy per atom (E_rel_) is with respect to the most stable configuration. Gray, violet, red, and yellow balls represent Re, Mo, V, and S atoms, respectively.

(a) Survey scan


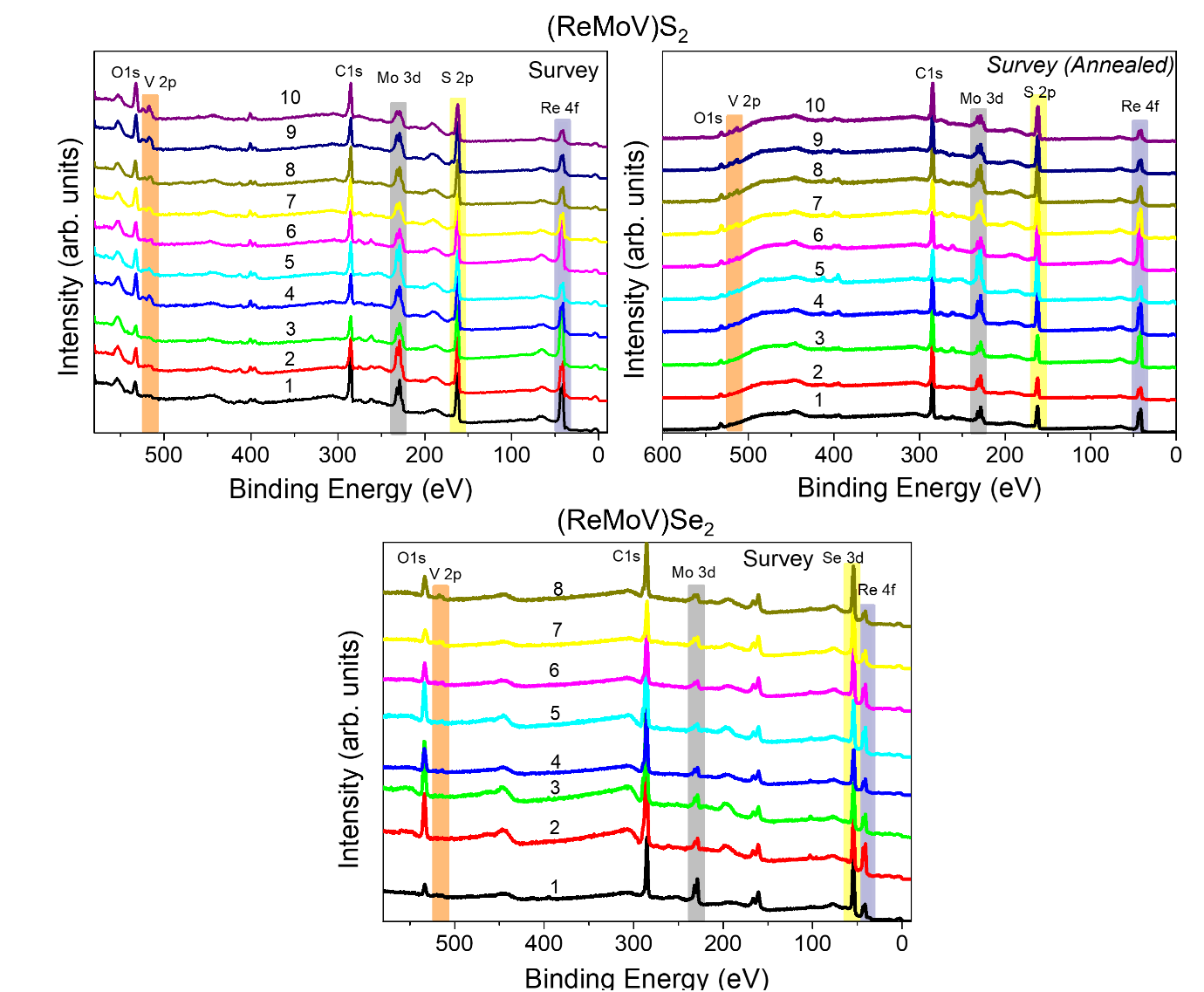


(b) Valence Band spectum (VBS)


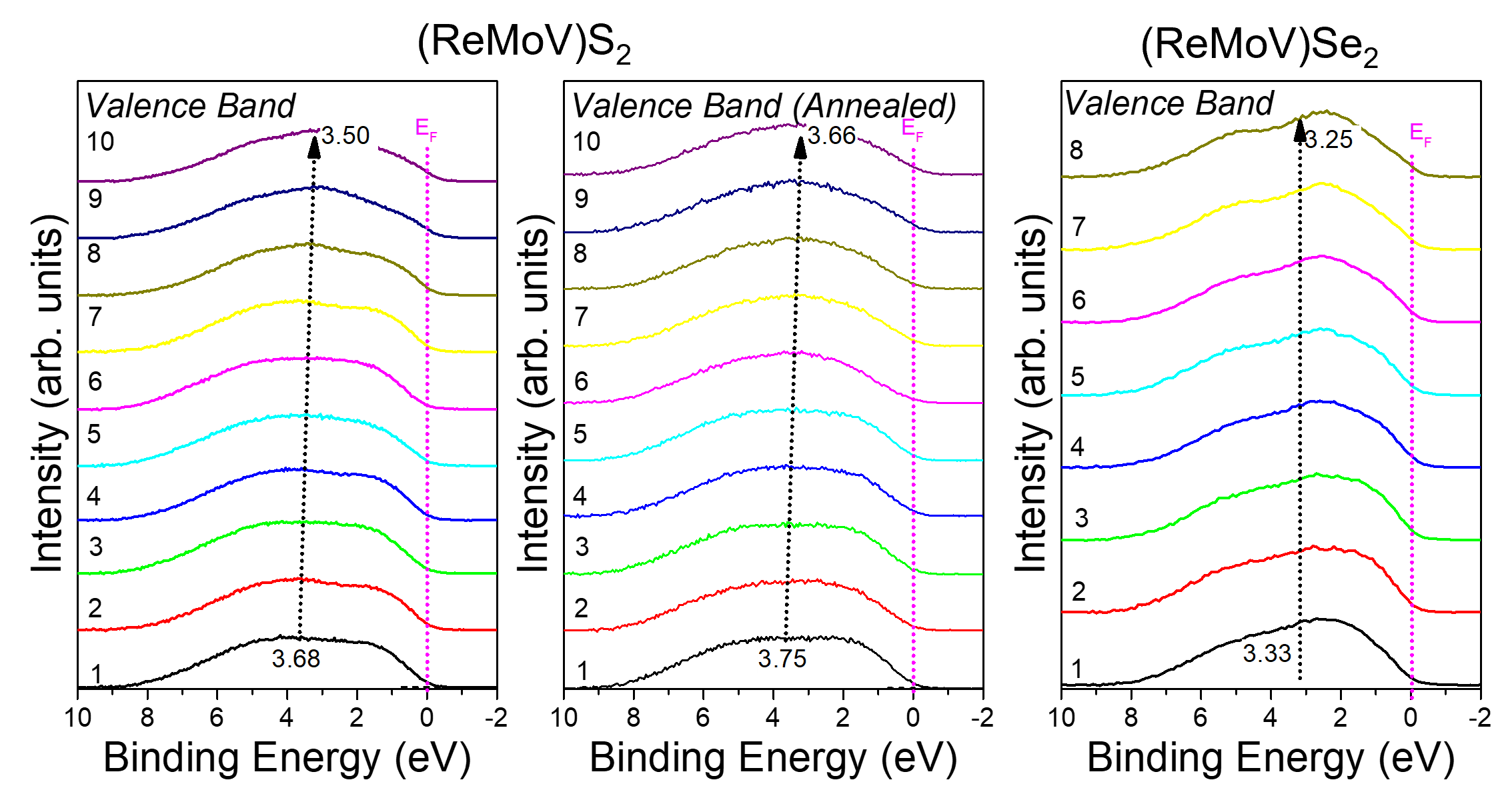


(c) Re 4*f*
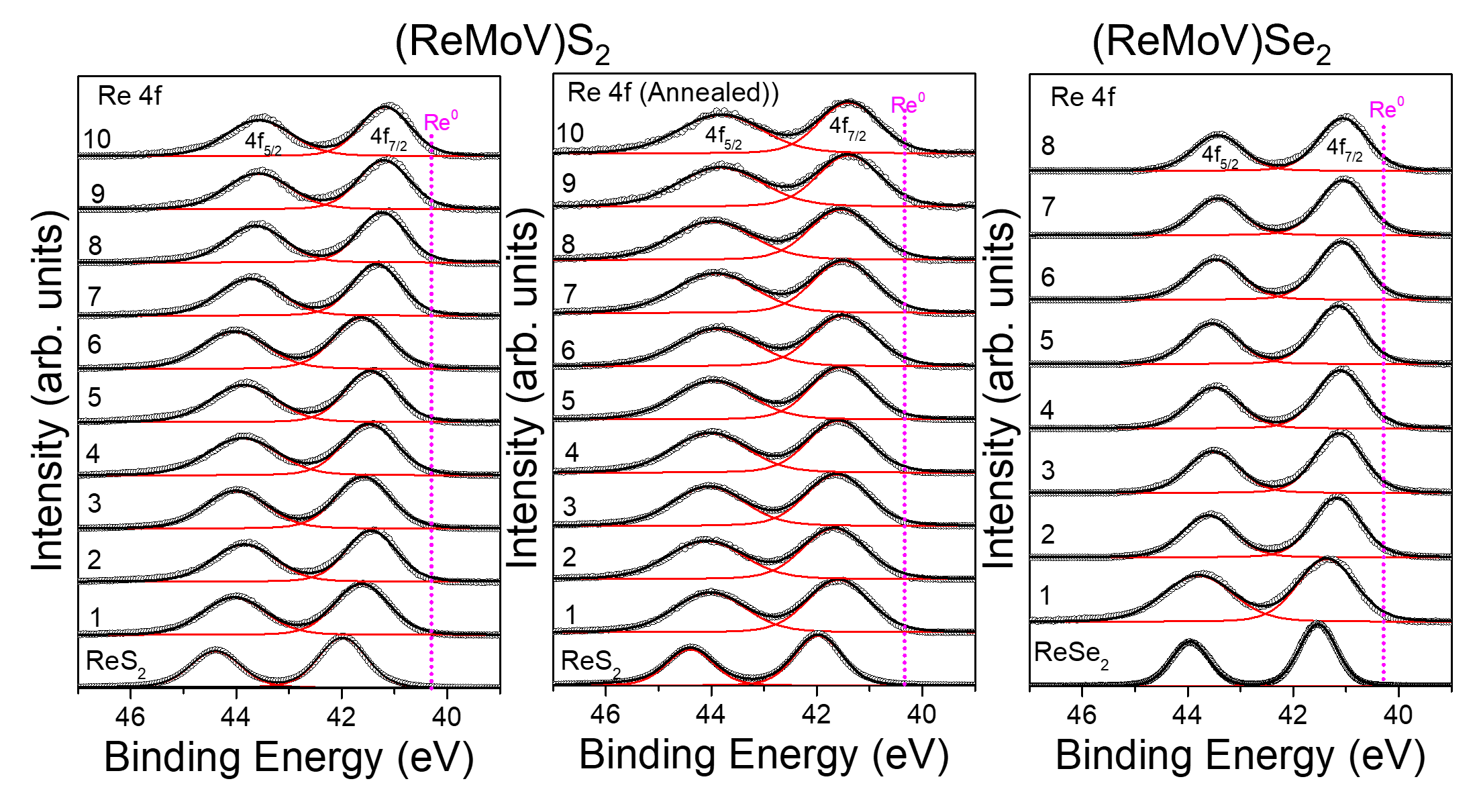


(d) Mo 3*d*
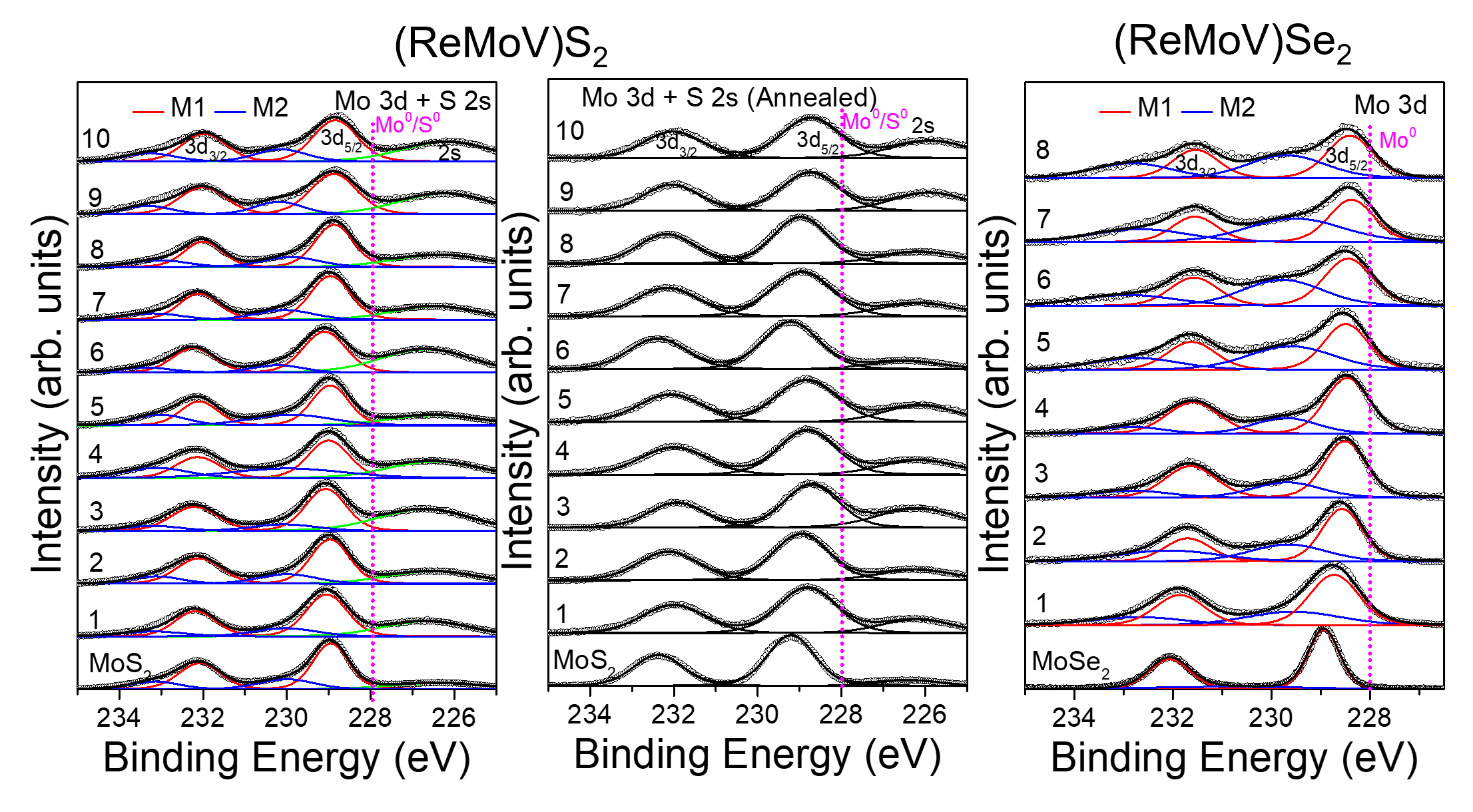


(e) V 2*p*


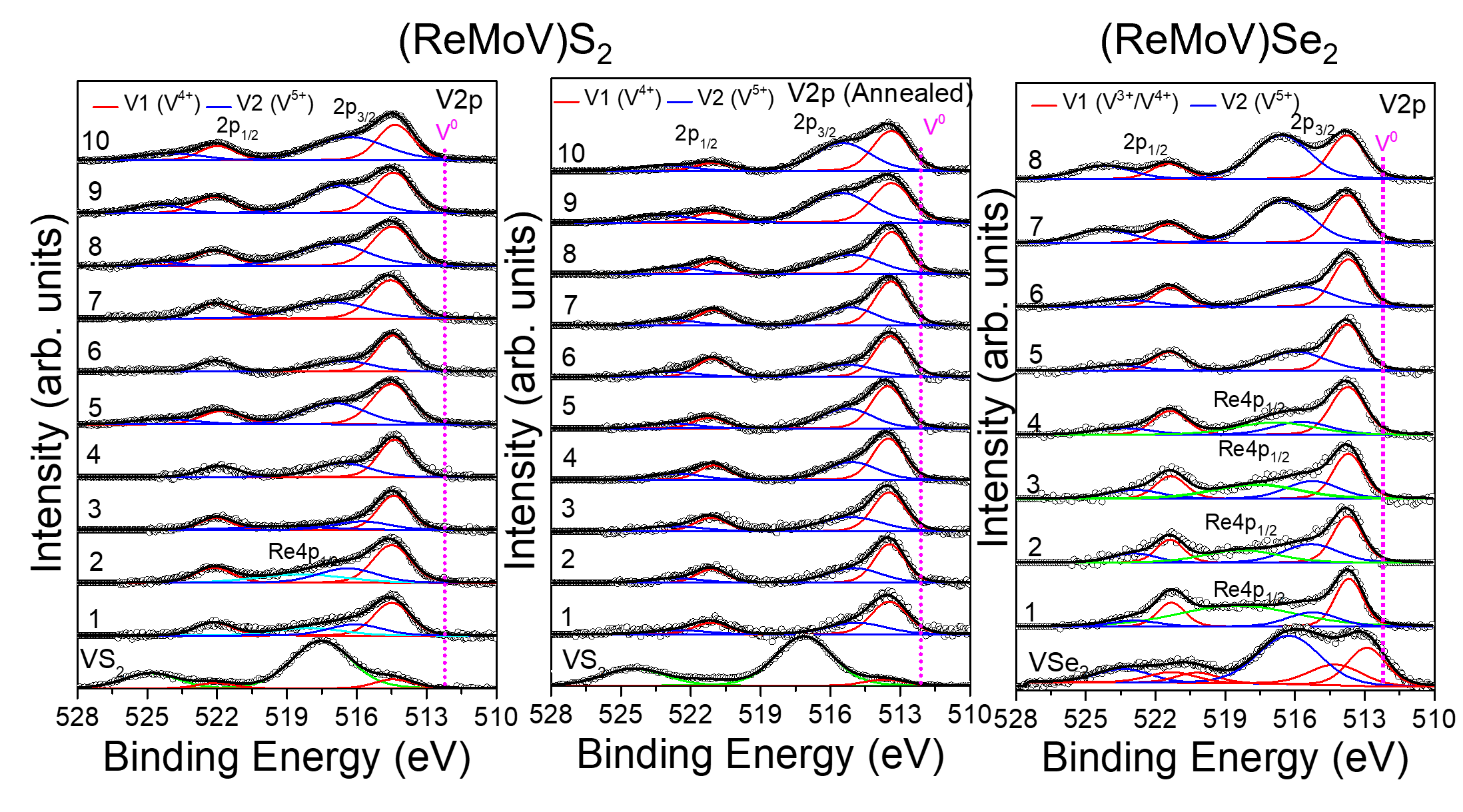


(f) S 2*p* or Se 3*d*


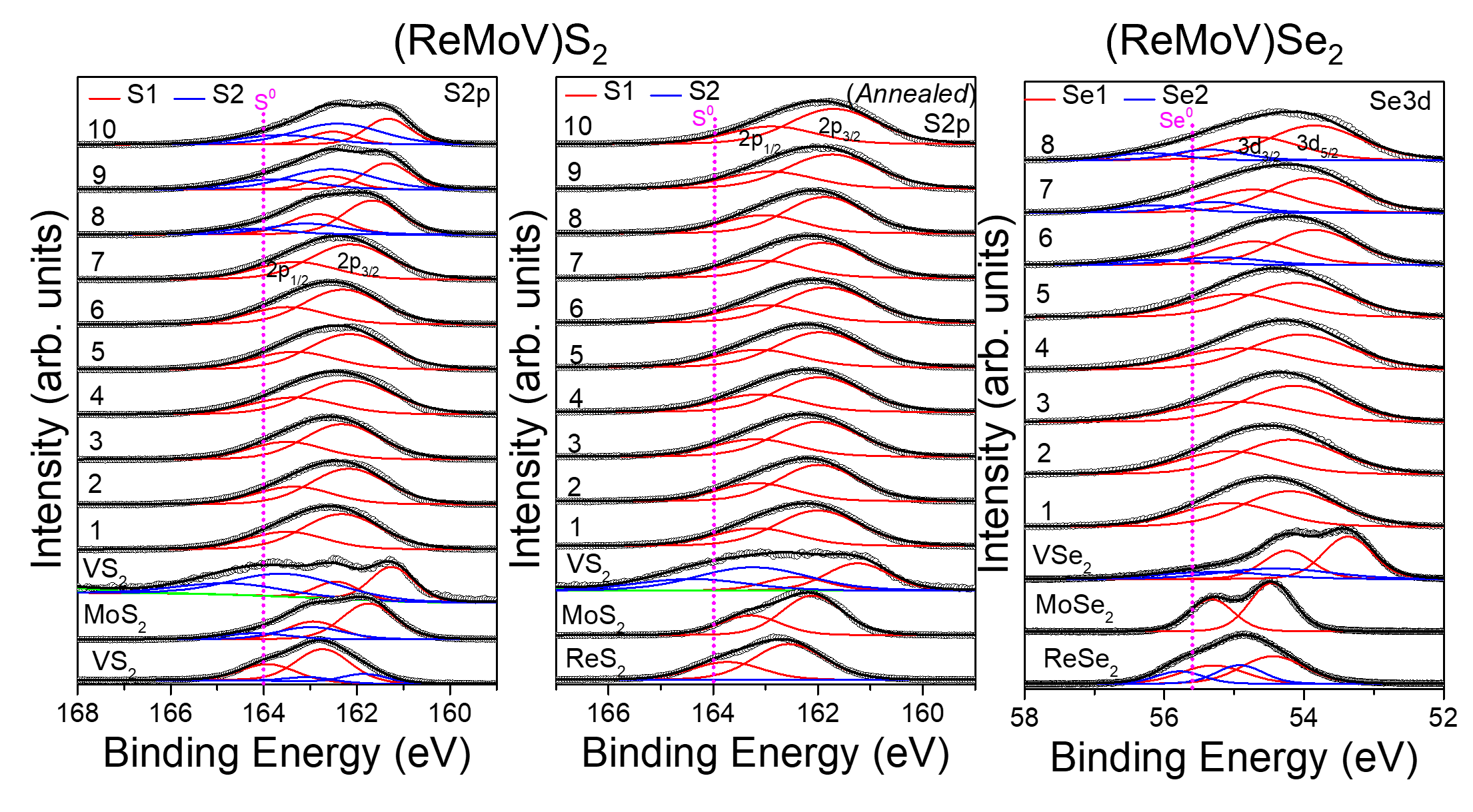


(g) N 1s


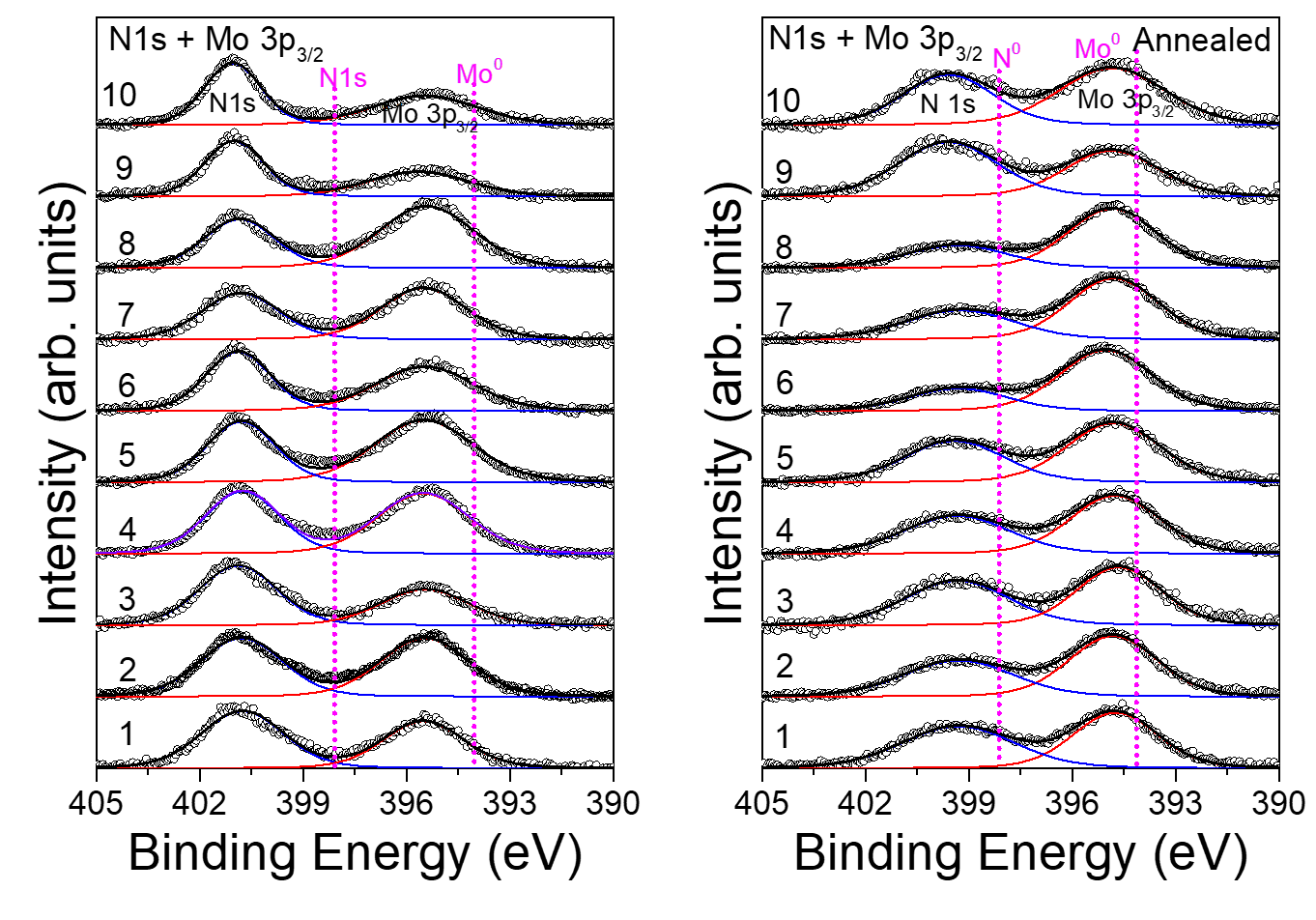


(h)


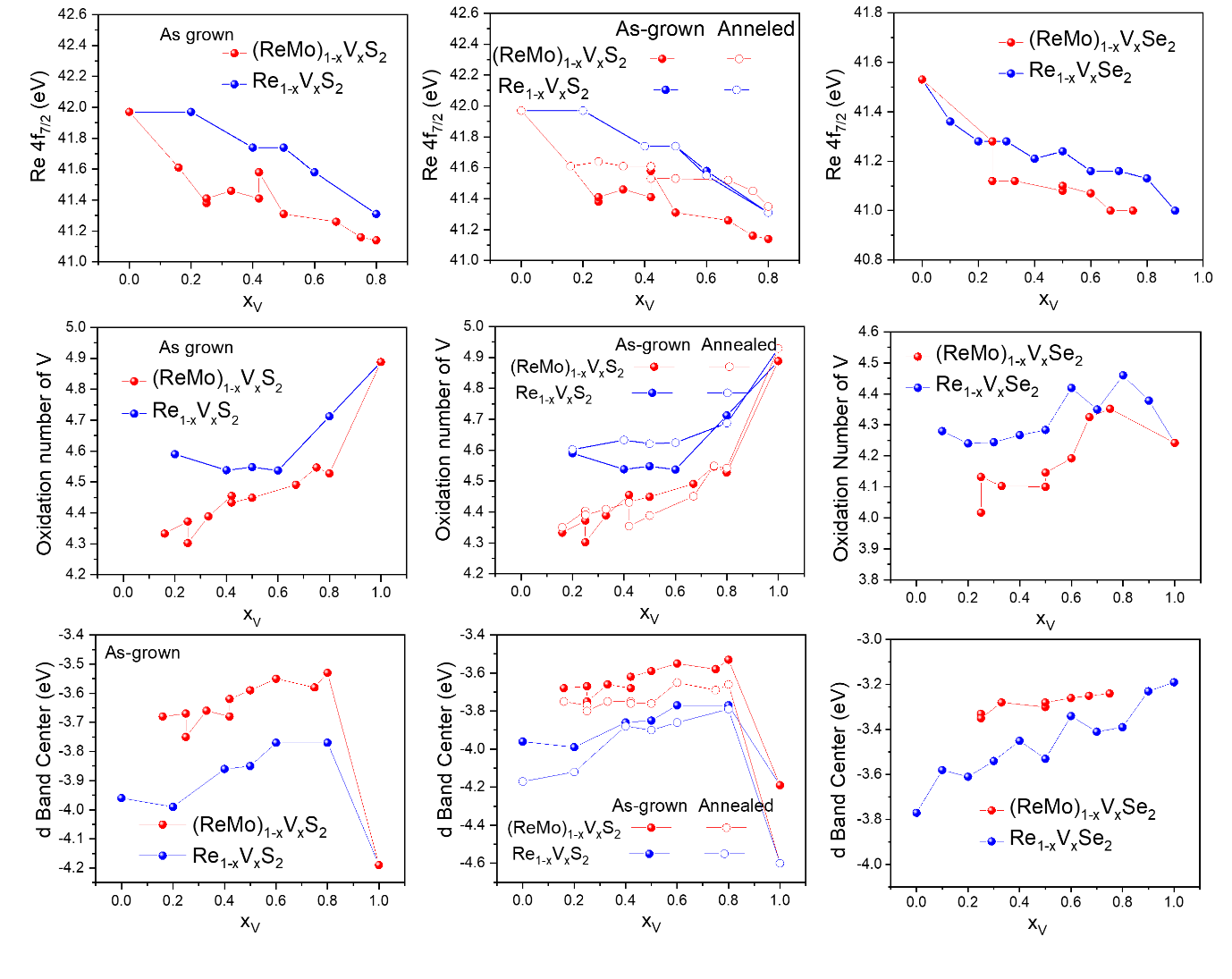


**Figure S5**. XPS data of as-grown/annealed (ReMoV)S_2_ and (ReMoV)Se_2_ samples using the photon energy of 650 eV: (a) survey scan, (b) valence band spectrum (VBS), fine-scan (c) Re 4*f*, (d) Mo 3*d*, (e) V 2*p*, (f) S 2*p/*Se 3*d*, and (g) N 1*s*. The background was corrected using a Shirley-type baseline. The experimental data (open circles) in (c)-(g), are fitted by a Voigt function, after the background correction with a Shirley-type baseline. The sum of the resolved bands is represented by a black line. Positions of Fermi level (E_F_ at 0 eV), neutral Re^0^ (4*f*_7/2_ at 31.4 eV), Mo^0^ (3*d*_5/2_ at 228.0 eV), S^0^ (1s at 228.0 eV), V^0^ (2*p*_3/2_ at 512.2 eV), S^0^ (2*p*_3/2_ at 164.0 eV), Se^0^ (3*d*_5/2_ at 55.6 eV), N^0^ (1*s* at 398.1 eV), and Mo^0^ (3*p*_3/2_ at 394.0 eV) are marked by dotted vertical lines. (h) Position of Re 4f_7/2_, oxidation number of V atoms, and the *d*-band center *vs.* *x*_V_.

(a) Survey scan shows that the V peak intensity increases with *x*_V_, while Re and Mo peaks decreases.

(b) The position of the valence band maximum (VBM) below the Fermi energy level (E_F_) was evaluated by linear extrapolation of the onset. All samples show VBM = 0 eV. The integration of VBS up to 10.0 eV with respect to E_F_ (binding energy = 0 eV) provided the *d-*band center (ε_d_). The *d*-band center upshifts continuously with increasing *x*_V_, as indicated by arrows. that the *d-*band center upshifts continuously with increasing *x*, due to the increase of metallic characters. Annealing of sulfide samples shifts the ε_d_ value to be larger negative due to the oxidation.

(c) The Re 4*f*_7/2_ and 4*f*_5/2_ peaks are separated by 2.43 eV. As-grown/annealed ReS_2_ samples shows the Re 4*f*_7/2_ peak at 42.0 eV. As *x*_V_ increases, the peak of as-grown samples redshifts continuously from 41.7 eV (sample **1**) to 41.2 eV (sample **10**) by 0.5 eV. The annealed samples show the redshift from 41.5 eV (sample **1**) to 41.1 (sample 10) by 0.4 eV. The ReSe_2_ samples shows the Re 4*f*_7/2_ peak at 41.5 eV. As *x*_V_ increases, the peak of selenide samples redshifts continuously from 41.3 eV (sample **1**) to 41.0 eV (sample **8**) by 0.3 eV. The red shift indicates that the alloying increased the metallicity.

(d) The Mo 3*d*_5/2_ and 3*d*_3/2_ peaks are separated by 3.13 eV. The S 1*s* peak appears at 226.5 eV, which is redshifted from S^0^ (at 228.0 eV). As-grown and annealed MoS_2_ samples shows the Mo 3*d*_5/2_ peak at 229.0 and 229.2 eV, respectively. As *x*_V_ increases, the peak of as-grown sulfide samples redshifts from 229.0 eV (sample **1**) to 228.8 eV (sample **10**) by 0.2 eV. The Mo 3*d* peaks were resolved into the M1 and M2 bands that correspond to Mo-S bonds and defects, respectively. The S 1*s* peak appears at 226.5 eV, which is redshifted from that of S^0^ at 228.0 eV. The annealed sulfide samples showed the redshift from to 228.8-228.9 eV at all compositions. The MoSe_2_ samples shows the Mo 3*d*_5/2_ peak at 228.8 eV. As *x*_V_ increases, the peak redshifts from 228.7 eV (sample **1**) to 228.4 eV (sample **8**) by 0.3 eV.

(e) The V 2*p*_3/2_ and V 2*p*_1/2_ peaks are separated by 7.64 eV. For the sulfide samples, each peak was resolved into two bands (V1 and V2). For the V 2*p*_3/2_ peak, The V1 band at 514 eV and V2 band at 517-518 eV are assigned to the V-S and V-O bonding structures, respectively. For VS_2_, the fraction of V2 band becomes close to 1, indicating that the VS_2_ nanosheets are highly oxidized. The samples **1**(S) and **2**(S) show the low-intensity band at 519 eV, which is ascribed to Re 4*p*_1/2_ that is blue shifted from neutral Re at 518.0 eV. The fraction of V2 band shows an increase with increasing *x*_V_. The oxidation number of V is calculated using the fraction of V1 and V2 bands of V 2*p*_3/2_ peak and plotted in (h). The annealed samples exhibit the higher oxidation number than that of as-grown samples.

For selenide samples, each peak was resolved into three bands (V1 and V2). The V1 and V2 bands are assigned to the V-S bonding structures with V^3+^/V^4+^ and V^5+^ ionic states, respectively. The V2 band is assigned to the V-O bonding structures. The samples **1~4** show the Re 4*p*_1/2_ band at 519 eV. The fraction of V2 band shows an increase with increasing *x*_V_. The oxidation number of V atom is calculated using the fraction of two bands of V 2*p*_3/2_ peak and plotted in (h).

(f) The S 2*p*_3/2_ and S 2*p*_1/2_ peaks are separated by 1.18 eV. The *x*_V_ increase induces gradually redshift of the peak. The peak was usually resolved into six bands: two each for the S^2-^ (S1 band) and the bridge (S_2_^2-^, defects) anions (S2 band). The VS_2_ sample shows the S3 band at 169 eV, which is assigned to the oxide form as S-O bonding structures. The position of S1 and S2 bands is 161-162 and 163-164 eV, respectively, which are 2-3 eV and 0-1 eV redshifted from S^0^ (at 164.0 eV). The S1 band redshifts with increasing *x*_V_, because of more metallic phase at higher *x*_V_.

The Se 3*d*_5/2_ and Se 3*d*_3/2_ peaks are separated by 0.86 eV. The *x*_V_ increase induces gradually redshift of the peak from 54.5 eV (sample **1**) to 54 eV (sample **8**). The ReS_2_ and VSe_2_ show two each for the Se binding with V^3+^/V^4+^ (Se1) and defects (Se2), respectively. The peak of samples **6**-**8** was resolved into four bands: two each for the Se binding with Re^4+^/V^3+/4+^ (Se1 band), and defects (Se2 band), respectively. The MoSe_2_ show one Se1 band. For the samples **1-5**, the peak was resolved with Se1 bond. The S1 band redshifts with increasing *x*_V_, because of more metallic phase at higher *x*_V_.

(g) The peak position of N 1s at 400 eV corresponds to the amine/amide form of intercalants. The Mo 3*p*_3/2_ peak appears at 395.3 eV, which is blue shifted from Mo^0^ (3*p*_3/2_ at 394.0 eV). After annealing, the peak intensity of N 1s decreases significantly.

(h) The position of Re 4*f*_7/2_ *vs. x*_V_ is plotted for as-grown/annealed ternary alloy and binary alloy samples. For the as-grown sulfides, the redshift (from 42.0 eV of ReS_2_ to 41.1 eV at *x*_V_ = 0.8) of ternary samples is more significant than that (41.3 eV at *x*_V_ = 0.8) of binary counterparts, indicating more metallic properties than the binary alloys. The annealed samples also exhibited more redshift than that of binary counterparts. For selenides, the redshift (from 41.5 eV to 41.0 eV at *x*_V_ =0.8) is more significant than that of Re_1-_*_x_*V*_x_*Se_2_ (to 41.1 eV at *x*_V_ = 0.8), indicating more metallic properties than the binary alloys. The oxidation number of V atoms *vs.* *x*_V_ is plotted for as-grown/annealed ternary alloy and binary alloy samples. The ternary samples show the smaller oxidation number than the binary samples. The *d*-band center (ε_d_) *vs.* *x*_V_ is plotted for as-grown/annealed ternary alloy and binary alloy samples. The ternary samples show the smaller (negative) ε_d_ value than the binary samples.

(a)


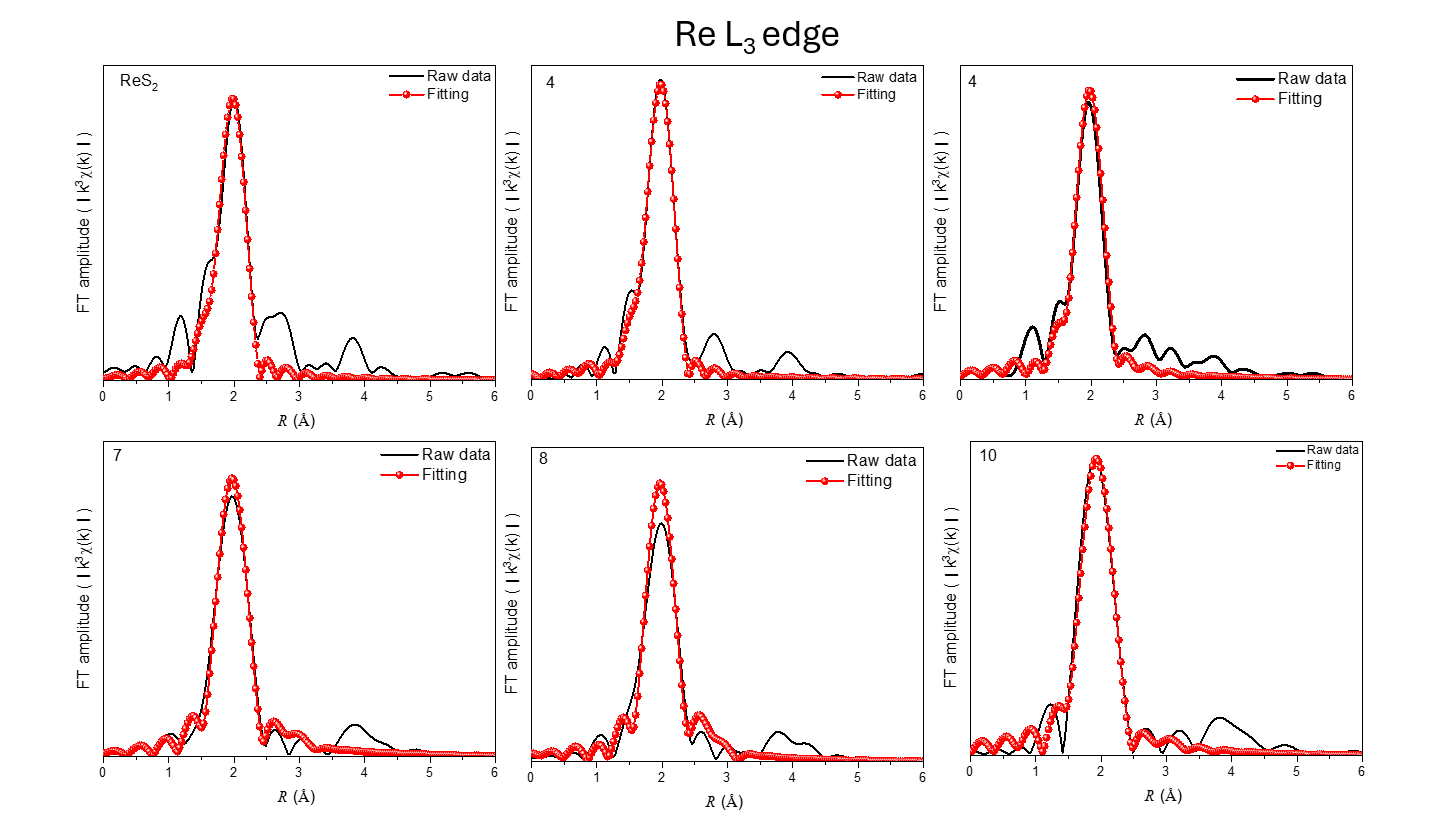

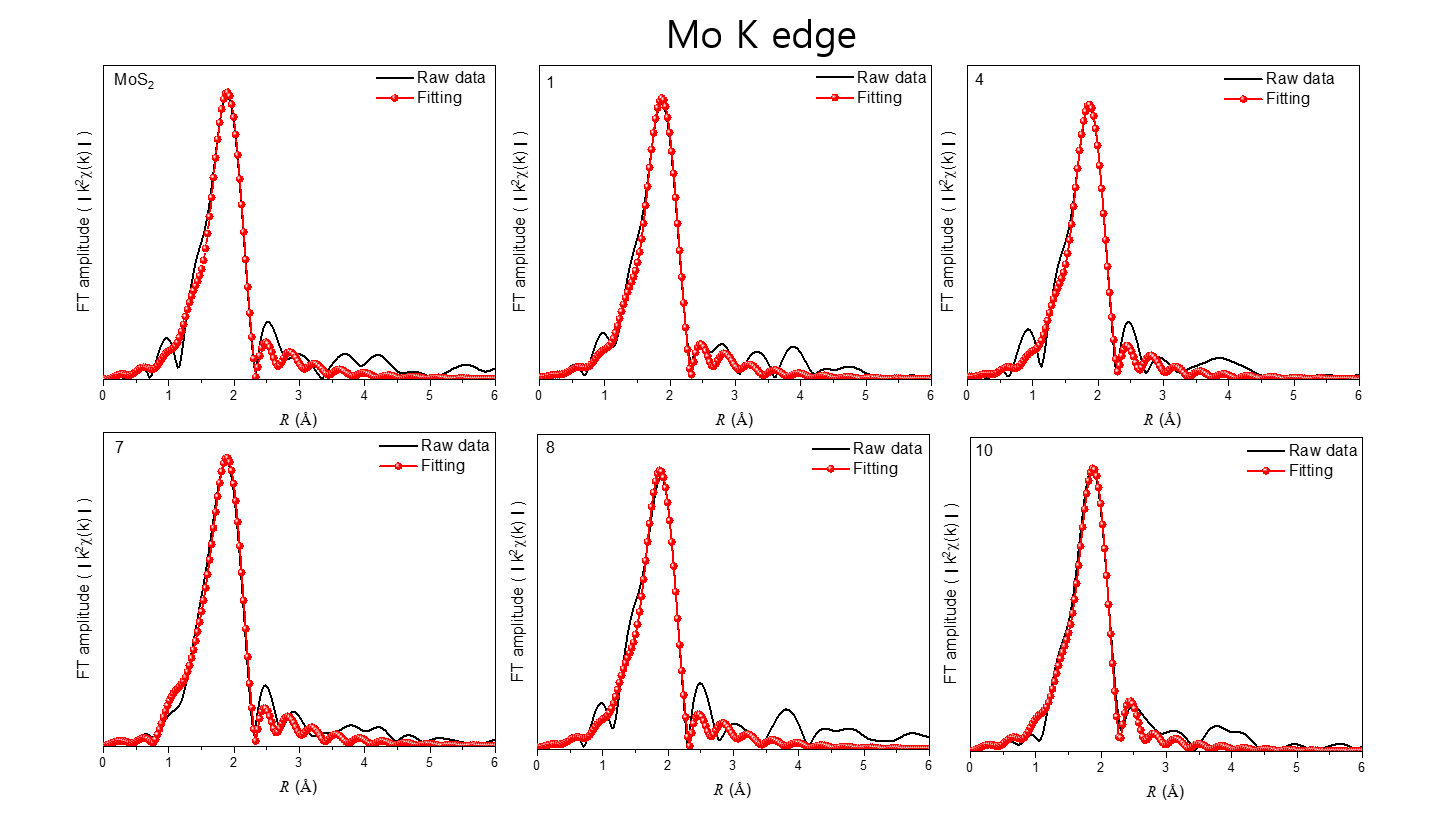


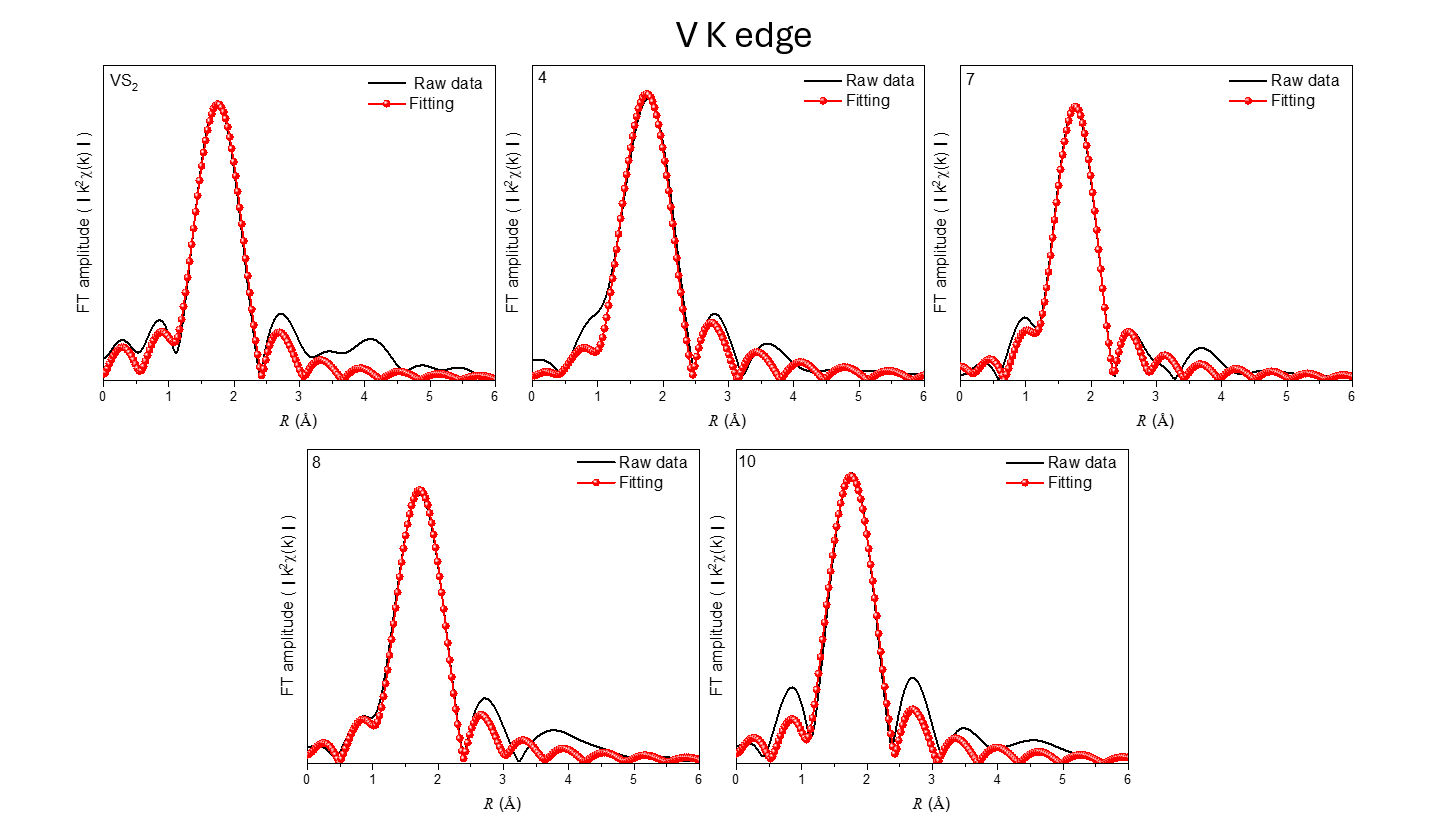


(b)


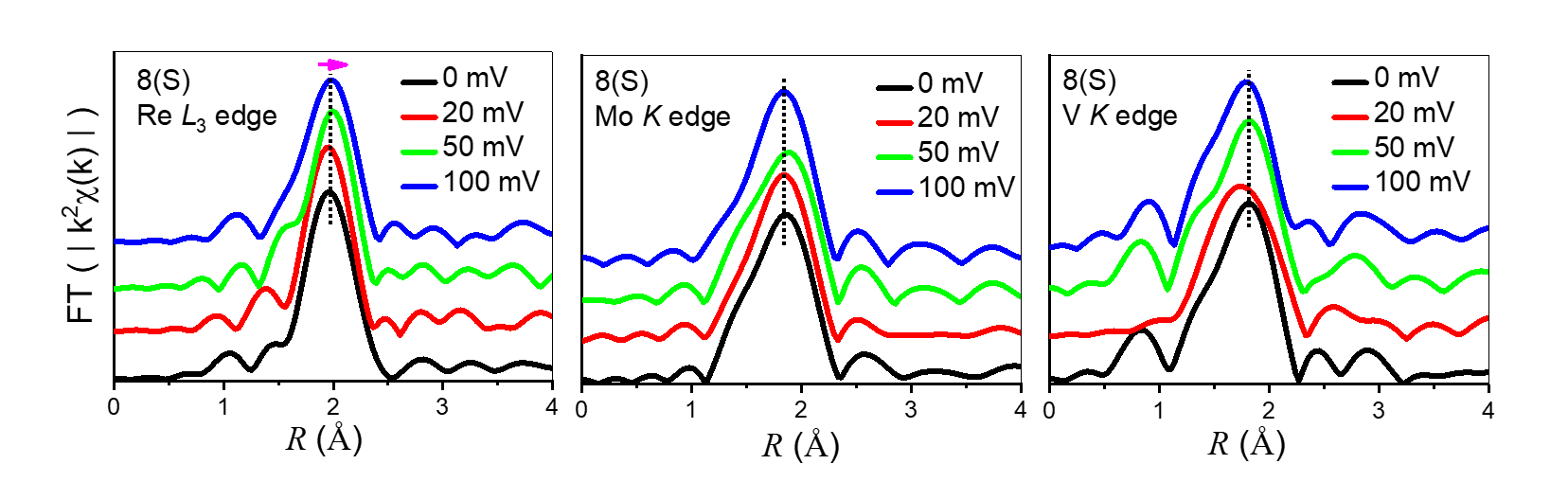


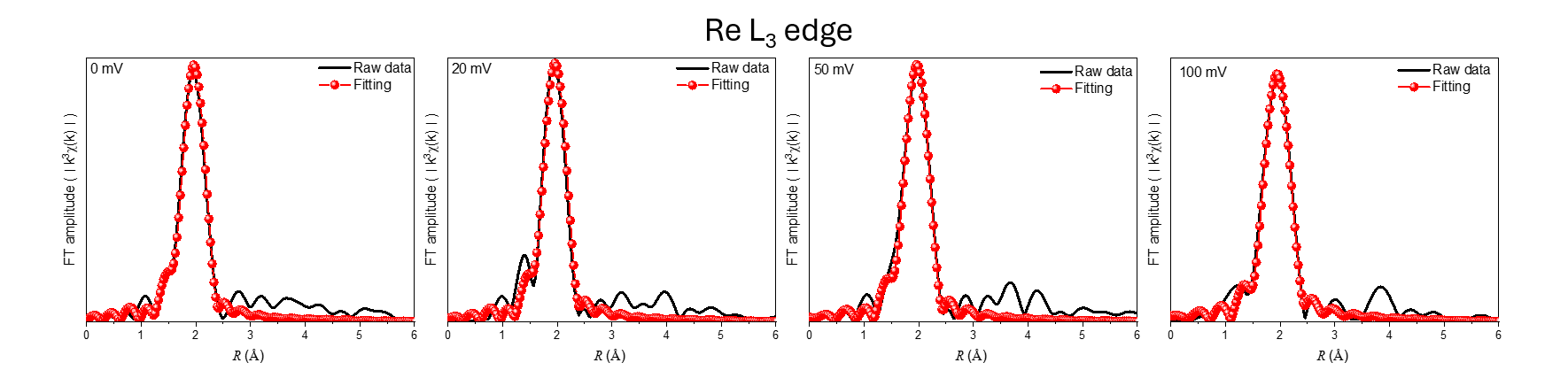


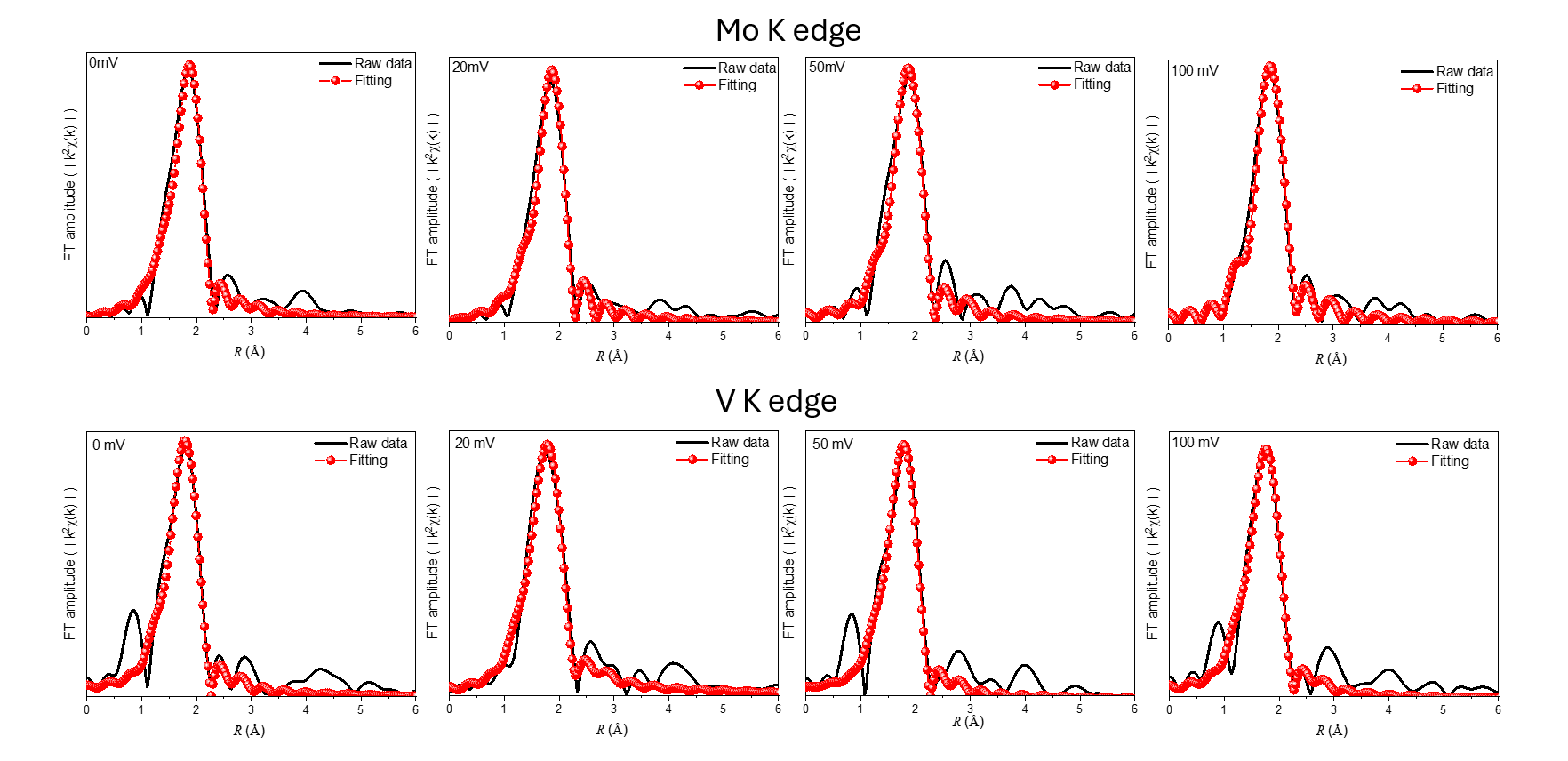


**Figure S6**. Curve fitting of k^2^-weighted FT EXAFS above the Re L_3_-edge (10.53 keV), Mo K-edge (20.01 keV), and V K-edge (5.46 keV) of (a) as-grown (ReMoV)S_2_ samples. (b) *Ex situ* (non-phase-corrected) *k*^2^-weighted FT EXAFS above Re L_3_ edge, Mo K edge, and V K edge for *x*_V_ = 0.67 (sulfide sample **8**S) after applying overpotential (𝜂) = 0–100 mV versus RHE) under HER conditions (H_2_-saturated 0.5 M H_2_SO_4_), and their curve fitting curve. Interatomic distance and coordination number were obtained by the curve fitting. The fitting parameters are summarized in **Table S3**.


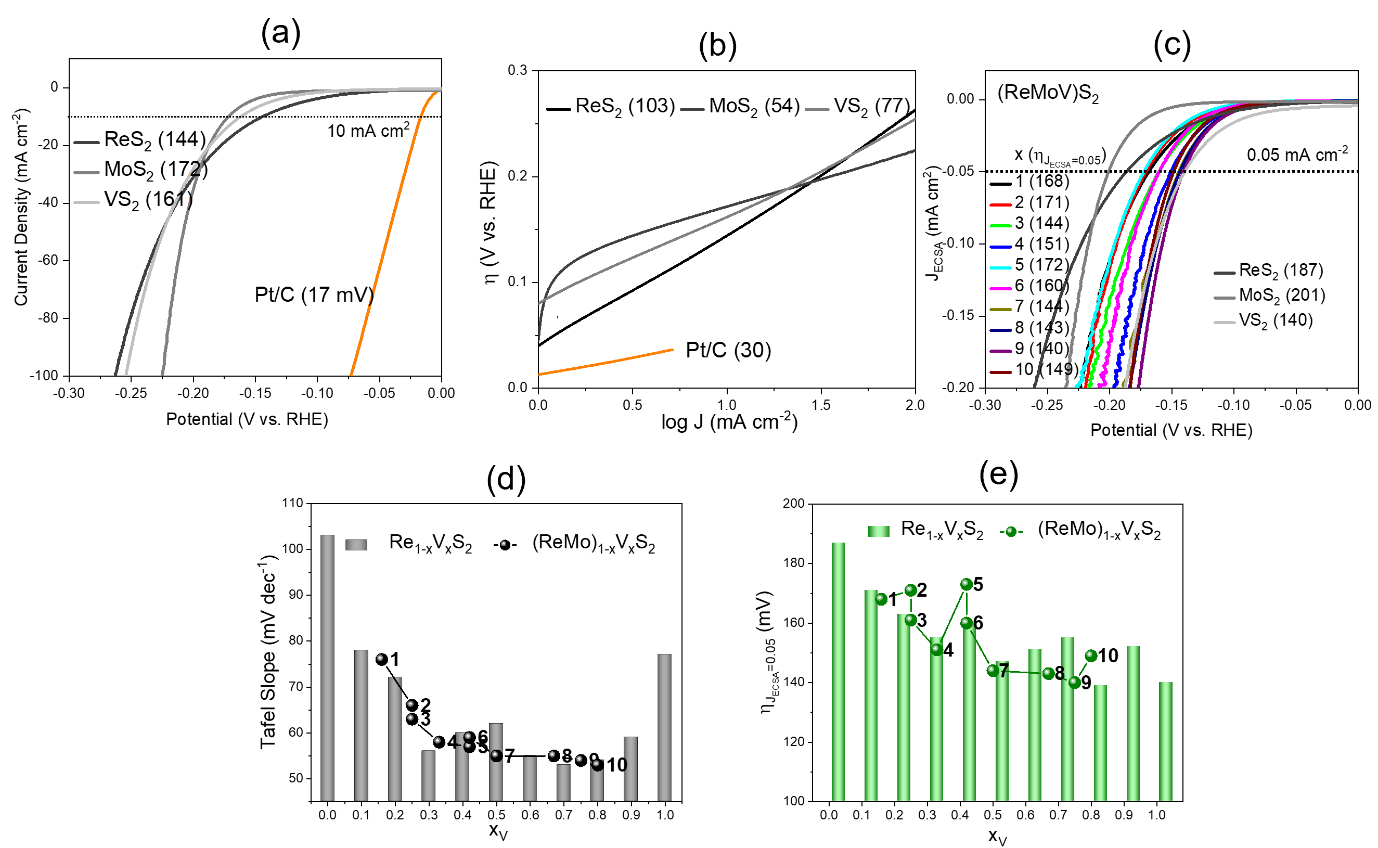
**Figure S7**. (a) HER LSV curves (scan rate: 2 mV s^–1^) and (b) Tafel plots for ReS_2_, MoS_2_, and VS_2_, in H_2_-saturated 0.5 M H_2_SO_4_. ECSA-normalized LSV curves for (c) ReS_2_, MoS_2_, VS_2_, and (ReMo)_1-_*_x_*V*_x_*S_2_ samples. (d) Tafel slope and (e) η_JECSA=0.05_ *vs. x*_V_ for the Re_1-_*_x_*V*_x_*S_2_ (columns) and (ReMo)_1-_*_x_*V*_x_*S_2_ samples (sphere symbols).


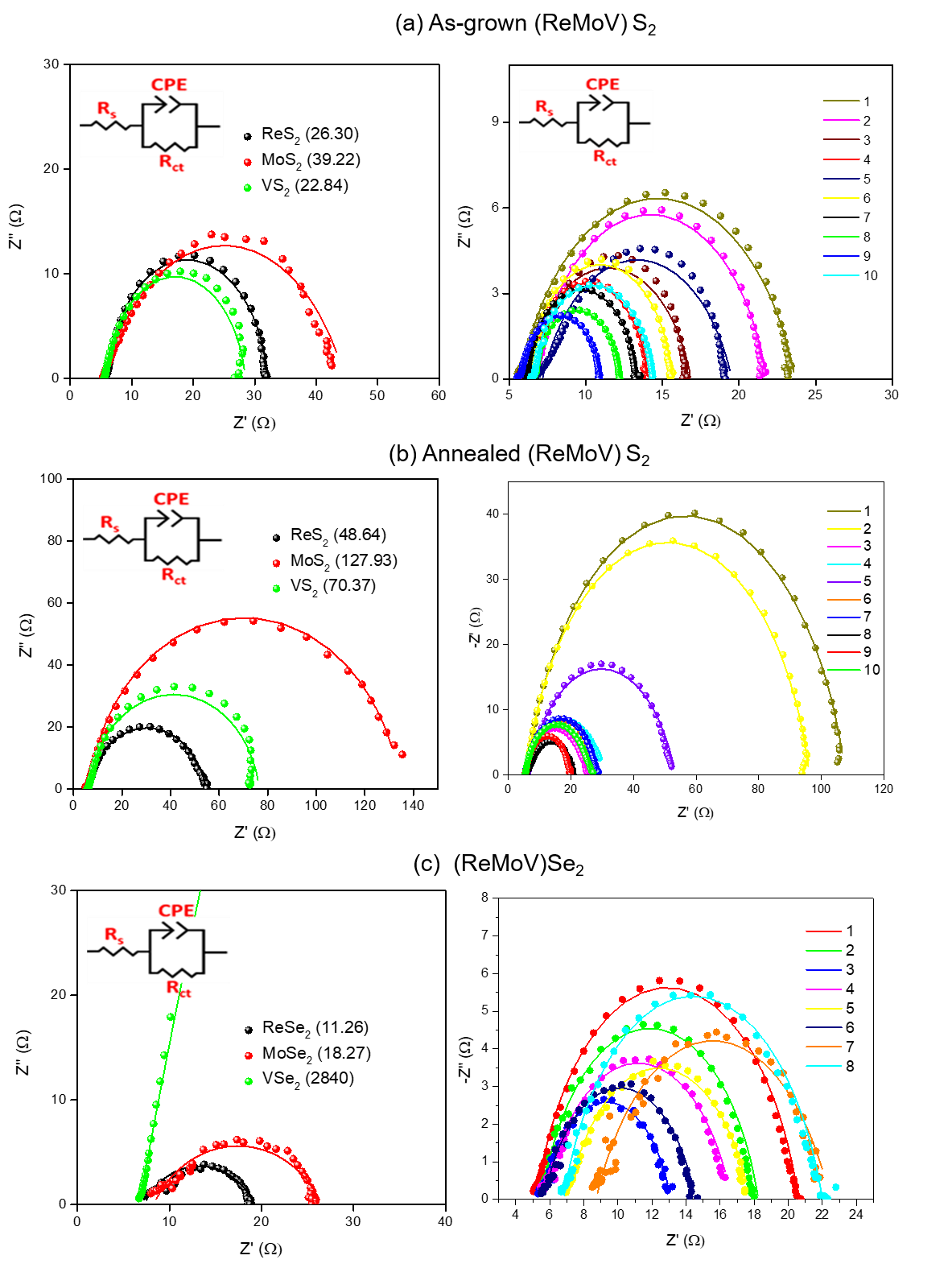


(d)


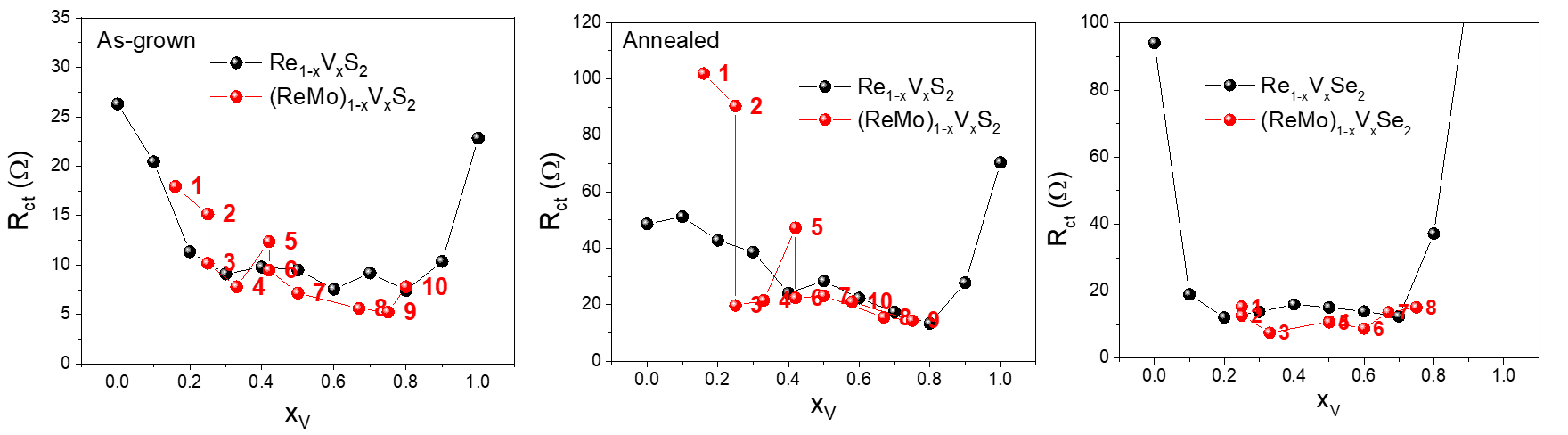
 **Figure S8**. Nyquist plots of (a) as-grown and (b) annealed ReS_2_, MoS_2_, VS_2_, and (ReMoV)S_2_ (= (ReMo)_1-_*_x_*V*_x_*S_2_), and (c) ReSe_2_, MoSe_2_, VSe_2_, and (ReMoV)Se_2_ (= (ReMo)_1-_*_x_*V*_x_*Se_2_) samples using the frequency in the range from 100 kHz to 0.1 Hz at a potential of -0.15 V (η = 150 mV). The modified Randles circuit for fitting is shown in the inset. The *R*_ct_ value *vs.* *x*_V_ for as-grown/annealed (ReMo)_1-_*_x_*V*_x_*S_2_ and (ReMo)_1-_*_x_*V*_x_*Se_2_ in comparison with Re_1-_*_x_*V*_x_*S_2_ (Ref. S30) and Re_1-_*_x_*V*_x_*Se_2_ (Ref. S29).

Electrochemical impedance spectroscopy (EIS) measurements of the samples were performed using an amplitude of 10 mV. In the high-frequency limit and under non-Faradaic conditions, the electrochemical system is approximated by the modified Randles circuit shown in the inset, where *R*_s_ denotes the solution resistance, CPE is a constant-phase element related to the double-layer capacitance, and *R*_ct_ is the charge-transfer resistance from any residual Faradaic processes. A semicircle in the low-frequency region of the Nyquist plots represents the charge transfer process, with the diameter of the semicircle reflecting the charge-transfer resistance. The real (*Z*′) and negative imaginary (-*Z*′′) components of the impedance are plotted on the *x* and *y* axes, respectively. The simulation of the EIS spectra using an equivalent circuit model allowed us to determine the charge transfer resistance, *R*_ct_, which is a key parameter for characterizing the catalyst-electrolyte charge transfer process. The fitting parameters are summarized as follows.

The *R_ct_* value of ternary samples was much larger than that of the unitary samples. A smaller *R_ct_* with increasing *x*_V_ implies more facile electron transfer kinetics that enhances the catalytic activity. In average, the *R*_ct_ value of (ReMo)_1-_*_x_*V*_x_*X_2_ samples was lower than that of the Re_1-_*_x_*V*_x_*X_2_ samples.

[Fitting Impedance parameters for the equivalent circuit]

(a) As-grown (ReMoV)S_2_ samples

| Sample No*.* | R_S_(Ω) | CPE (mF) | R_ct_ (Ω) |
| --- | --- | --- | --- |
| ReS_2_ | 5.87 | 0.12 | 26.30 |
| MoS_2_ | 5.39 | 0.15 | 39.22 |
| VS_2_ | 5.64 | 6.00 | 22.84 |
| 1 | 5.71 | 7.05 | 17.95 |
| 2 | 6.62 | 0.10 | 15.15 |
| 3 | 6.09 | 0.15 | 10.77 |
| 4 | 6.23 | 0.16 | 7.79 |
| 5 | 7.17 | 0.19 | 12.37 |
| 6 | 6.18 | 0.20 | 9.47 |
| 7 | 6.14 | 0.13 | 7.16 |
| 8 | 6.62 | 0.26 | 5.60 |
| 9 | 5.70 | 0.24 | 5.23 |
| 10 | 6.58 | 0.22 | 7.80 |

(b) Annealed (ReMoV)S_2_ samples

| *x* | R_S_(Ω) | CPE (mF) | R_ct_ (Ω) |
| --- | --- | --- | --- |
| ReS_2_ | 5.94 | 7.41 | 48.64 |
| MoS_2_ | 5.61 | 0.32 | 127.93 |
| VS_2_ | 6.11 | 7.62 | 70.37 |
| 1 | 5.70 | 1.96 | 102.9 |
| 2 | 5.64 | 1.81 | 90.43 |
| 3 | 5.25 | 0.12 | 19.74 |
| 4 | 5.89 | 0.18 | 21.54 |
| 5 | 6.06 | 0.73 | 47.27 |
| 6 | 5.80 | 0.17 | 22.37 |
| 7 | 5.27 | 0.13 | 23.20 |
| 8 | 5.77 | 0.24 | 15.54 |
| 9 | 5.35 | 0.20 | 14.37 |
| 10 | 5.30 | 0.10 | 21.02 |

(c) (ReMoV)Se_2_ samples

| *x* | R_S_(Ω) | CPE (mF) | R_ct_ (Ω) |
| --- | --- | --- | --- |
| ReSe_2_ | 7.26 | 4.21 | 11.26 |
| MoS_2_ | 7.92 | 3.60 | 18.27 |
| VS_2_ | 6.30 | 1.23 | 284022 |
| 1 | 5.19 | 6.27 | 15.40 |
| 2 | 5.50 | 5.56 | 12.74 |
| 3 | 5.50 | 0.11 | 7.60 |
| 4 | 5.68 | 0.10 | 10.98 |
| 5 | 7.0 | 9.32 | 10.73 |
| 6 | 5.66 | 0.11 | 8.87 |
| 7 | 8.70 | 0.11 | 13.76 |
| 8 | 6.80 | 9.16 | 15.20 |


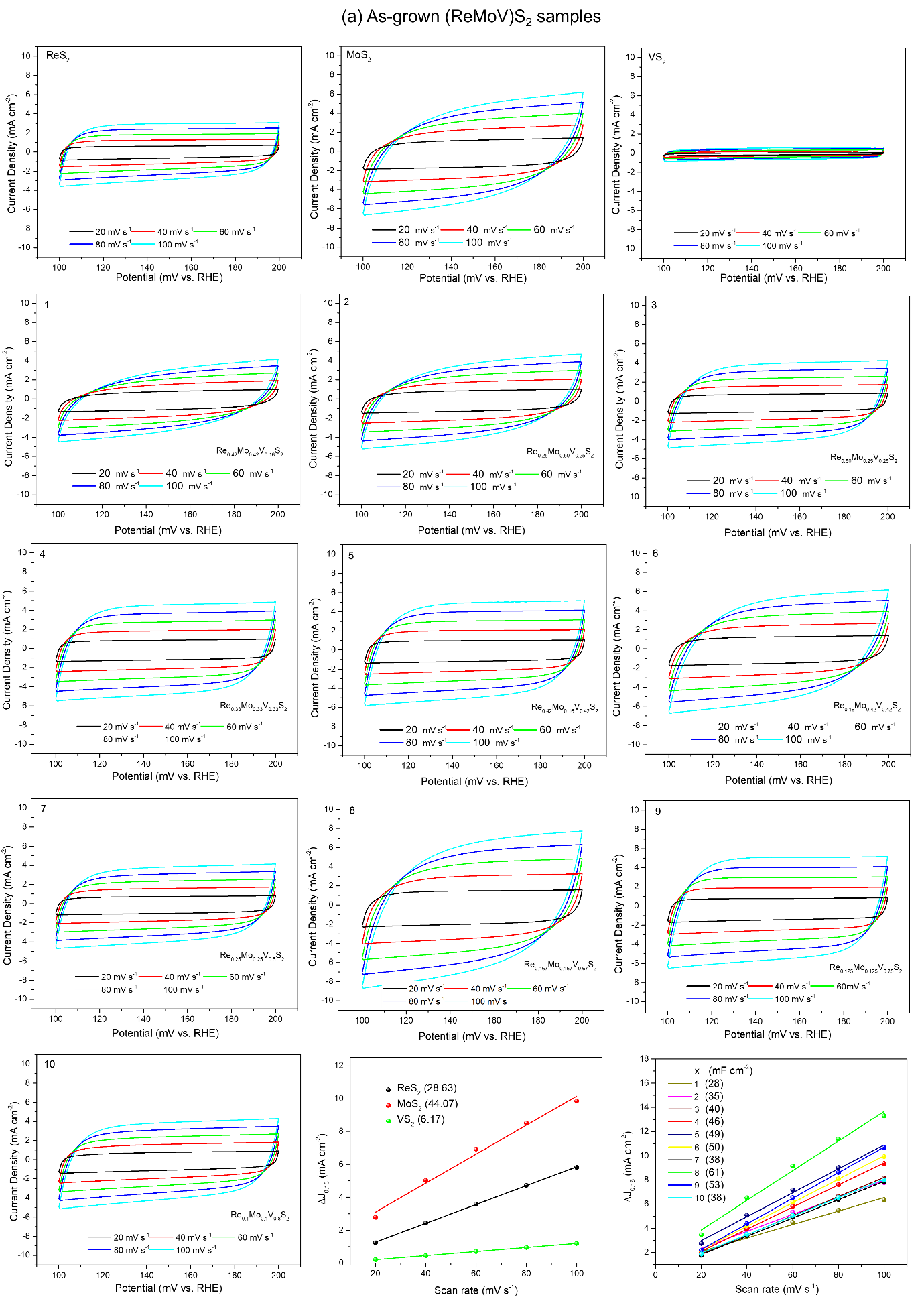

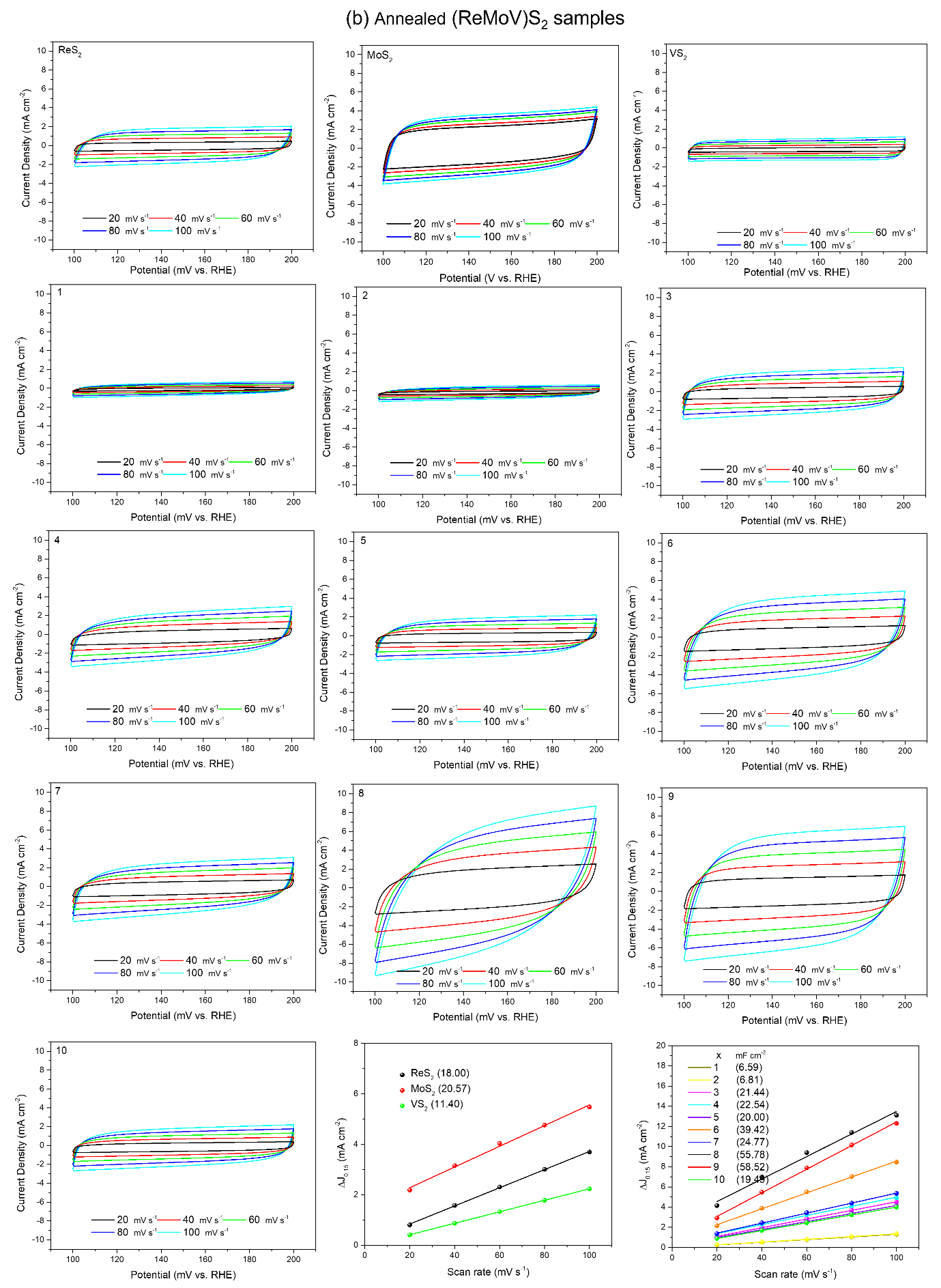

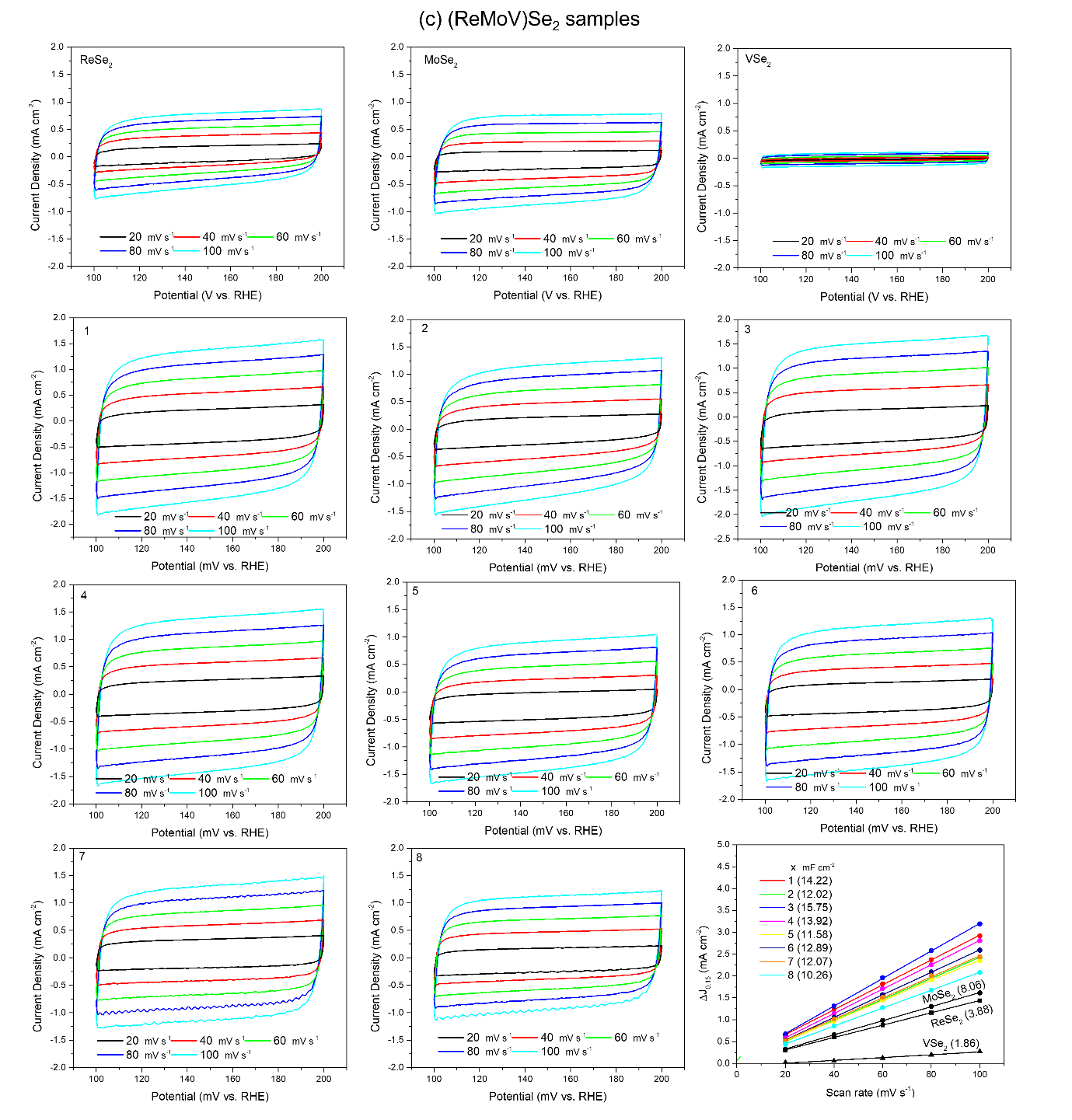
 (d)


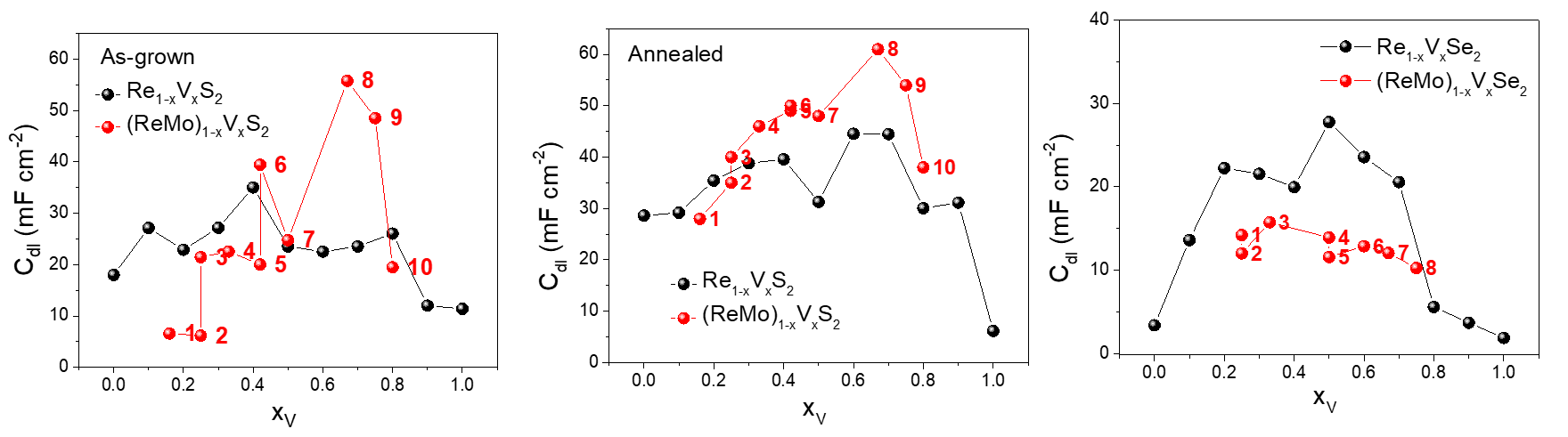


**Figure S9**. Cyclic voltammetry (CV) curves (in 0.5 M H_2_SO_4_) of (a) as-grown and (b) annealed ReS_2_, MoS_2_, VS_2_, and (ReMoV)S_2_ (= (ReMo)_1-_*_x_*V*_x_*S_2_), and (c) ReSe_2_, MoSe_2_, VSe_2_, and (ReMoV)Se_2_ (= (ReMo)_1-_*_x_*V*_x_*Se_2_) samples in a non-Faradaic region at 20-100 mV s^-1^ scan rates (with a step of 20 mV s^-1^). The scan range is 0.1−0.2 V *vs.* RHE, in 0.5 M H_2_SO_4_. Difference (Δ*J*) between the anodic charging and cathodic discharging currents measured at 0.15 V *vs.* RHE and plotted as a function of the scan rate. The value in parenthesis represents the C*_dl_*, obtained by the half of the linear slope. The C*_dl_* value of ternary samples was much larger than that of the unitary samples. (d) The C*_dl_* value *vs.* *x*_V_ for as-grown/annealed (ReMo)_1-_*_x_*V*_x_*S_2_ and (ReMo)_1-_*_x_*V*_x_*Se_2_ in comparison with Re_1-_*_x_*V*_x_*S_2_ (Ref. S30) and Re_1-_*_x_*V*_x_*Se_2_ (Ref. S29). The C*_dl_* value of sulfide ternary samples was generally larger than that of the binary samples. In contrast. selenide ternary samples exhibited the smaller C*_dl_* than that of the binary samples.


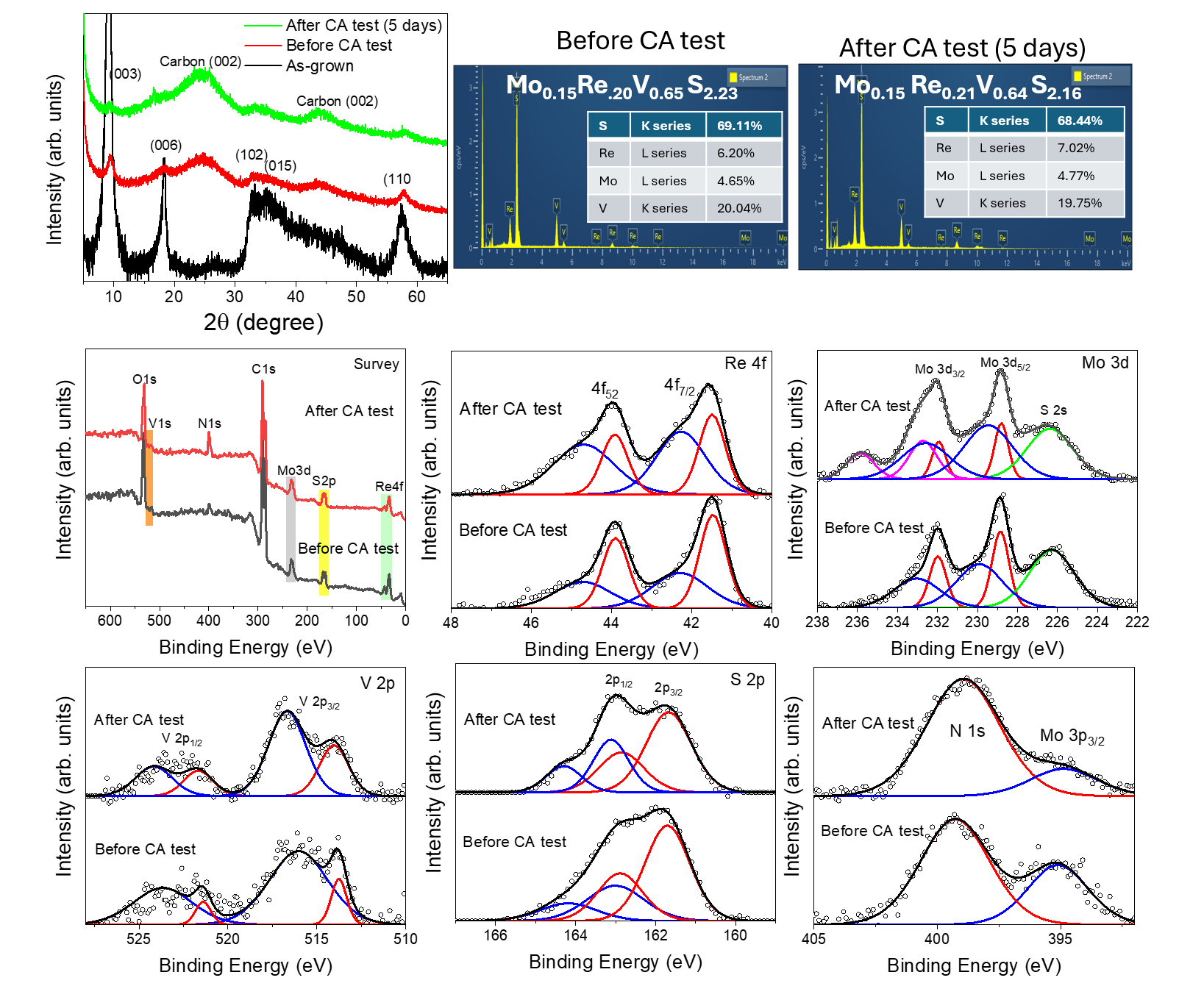
**Figure S10**. Characterization of *x*_V_ *=* 0.67 sample (sulfide sample **8**S) before/after 5 days CA test. XRD patterns showed that the phase with expanded interlayers maintains after CA test. The EDX spectrum shows that the composition of Re, Mo, V, and S atoms remain the same. No Pt contamination from the dissolved Pt counter electrode was not detected. The XPS (using Al K α line) also confirmed that no Pt deposition occurred, and the metallic properties of the samples retained during the HER. The experimental data (open circles) are fitted by a Voigt function, after the background correction with a Shirley-type baseline. After the CA test, the adsorption of electrolyte results in the increase of the higher-oxidation states (blue color bands) for Re, Mo, V, and S. Nevertheless, the metallic properties remained.


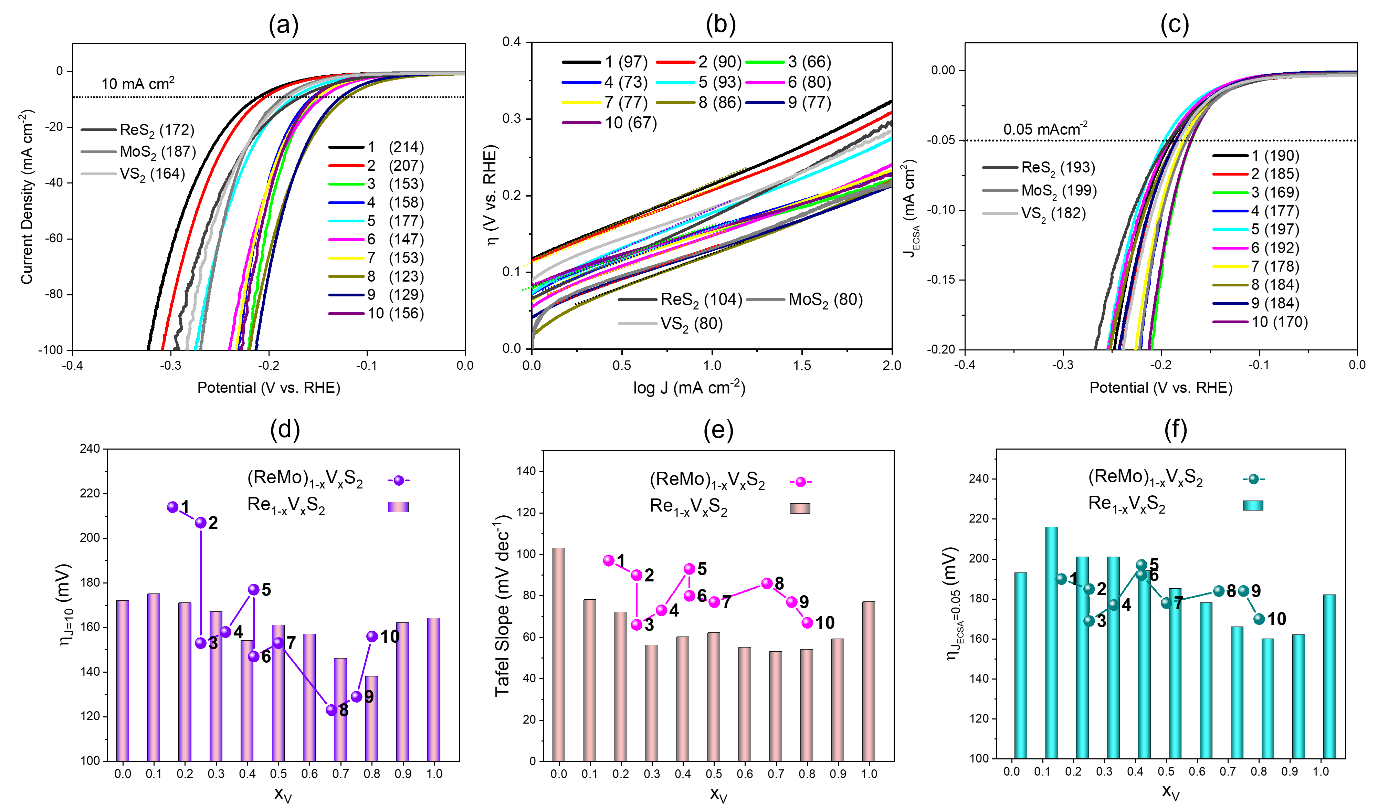
**Figure S11**. (a) LSV curves (scan rate: 2 mV s^–1^) for annealed (ReMoV)S_2_ (= (ReMo)_1-_*_x_*V*_x_*S_2_) samples toward HER in H_2_-saturated 0.5 M H_2_SO_4_. (b) Tafel plots derived from the LSV curves. (c) ECSA-normalized LSV curves. (d) η_J=10_, (e) Tafel slope, and (f) η_JECSA=0.05_ *vs. x*_V_ for the annealed (ReMo)_1-_*_x_*V*_x_*S_2_ (sphere symbols) and the annealed Re_1-_*_x_*V*_x_*S_2_ samples (columns, Ref. S30).

The electrocatalytic properties of annealed (ReMoV)S_2_ samples in 0.5 M H_2_SO_4_ were also studied. The measured parameters are summarized in **Table S4**. LSV curves exhibited the lowest η_J=10_ (123 mV) for sample **8**. The *R*_ct_ and *C*_dl_ values were also measured as shown in **Figures S9** and **S10**, respectively. The LSV curves using *J*_ECSA_ (= *J* divided by ECSA) shows the overpotential at *J*_ECSA_ = 0.05 mA cm^–2^ is in the range of 169-197 mV, which is in the narrower range than that of η_J=10_ (123-214 mV). The η_J_*_=_*_10_, Tafel slope, and η_JECSA_*_=_*_0.05_ values at each *x* are plotted for these annealed (ReMo)_1-_*_x_*V*_x_*S_2_ samples with those of the annealed Re_1-_*_x_*V*_x_*S_2_ samples. The comparison of η_J_*_=_*_10_ shows that the incorporation of MoS_2_ enhances the HER performance. The η_JECSA_*_=_*_0.05_ of the ternary alloy exhibited the lower value for the samples **1**-**7**. The η_JECSA_*_=_*_0.05_ of samples **8**-**10** is higher, probably due to much reduced ECSA that caused by the nonlayered V_5_S_8_/V_3_S_4_ phase. As shown in **Figure S5**, the metallicity increases with increasing *x*_V_ and the annealing makes less metallic due to the oxidation. The best HER performance of samples **8**-**10** is due to the increased metallicity.


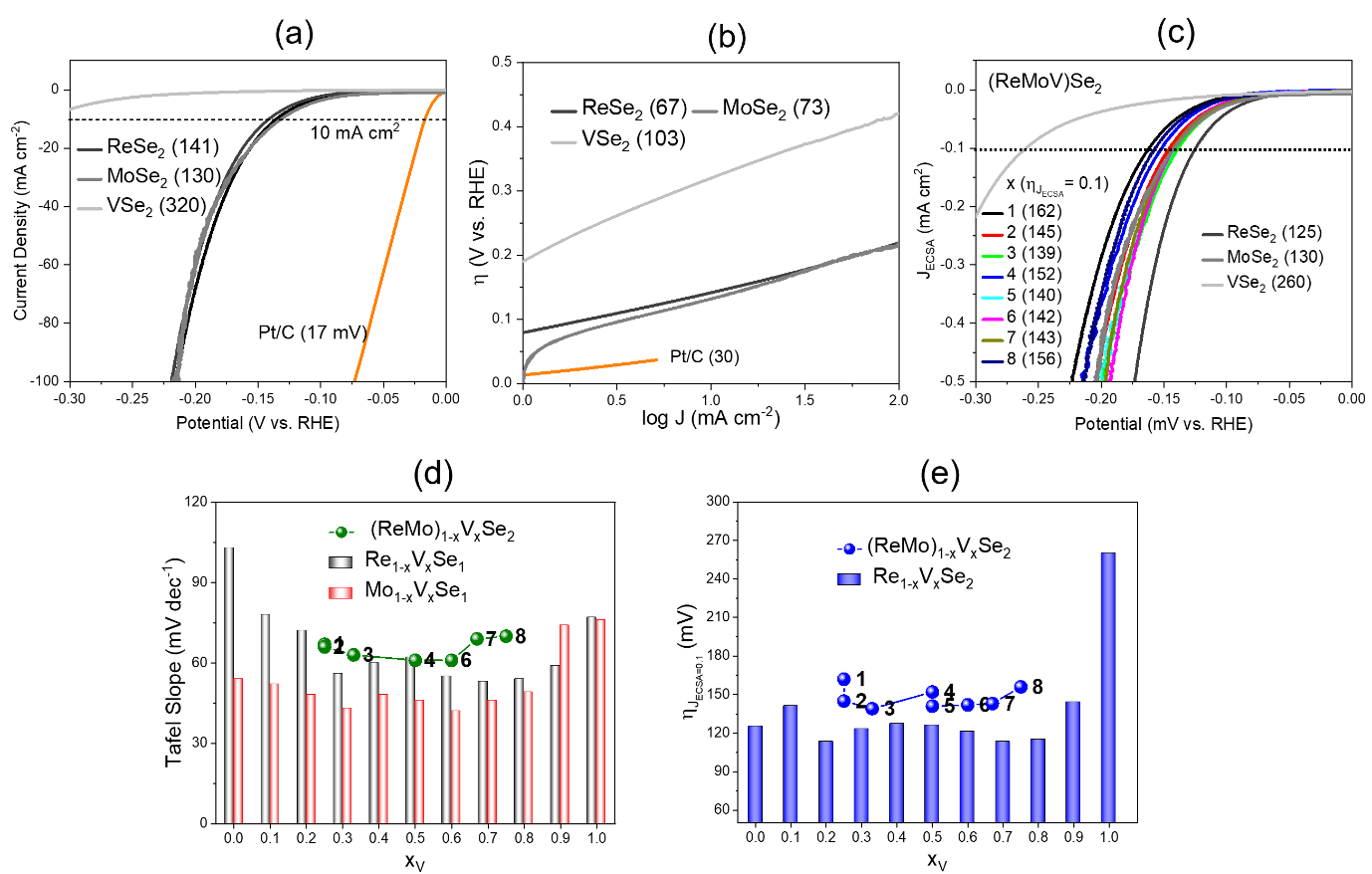
 **Figure S12**. (a) HER LSV curves (scan rate: 2 mV s^–1^) and (b) Tafel plots for ReSe_2_, MoSe_2_, and VSe_2_, in H_2_-saturated 0.5 M H_2_SO_4_. ECSA-normalized LSV curves for (c) ReSe_2_, MoSe_2_, VSe_2_, and (ReMo)_1-_*_x_*V*_x_*Se_2_ samples. (d) Tafel slope and (e) η_JECSA=0.1_ *vs. x*_V_ for (ReMo)_1-_*_x_*V*_x_*Se_2_ (sphere symbols) and Re_1-_*_x_*V*_x_*Se_2_ (Ref. S30)/Mo_1-_*_x_*V*_x_*Se_2_ (Ref. S24) samples (column).

**References**

1. (a) Li, L.; Qin, Z.; Ries, L.; Hong, S.; Michel, T.; Yang, J.; Salameh, C.; Bechelany, M.; Miele, P.; Kaplan, M.; Chhowalla, M.; Voiry, D. Role of Sulfur Vacancies and Undercoordinated Mo Regions in MoS_2_ Nanosheets toward the Evolution of Hydrogen. *ACS Nano* **2019**, *13*, 6824-6834. (b) Yin, Y.; Han, J.; Zhang, Y.; Zhang, X.; Xu, P.; Yuan, Q.; Samad, L.; Wang, X.; Wang, Y.; Zhang, Z.; Zhang, P.; Cao, X.; Song, B.; Jin, S. Contributions of Phase, Sulfur Vacancies, and Edges to the Hydrogen Evolution Reaction Catalytic Activity of Porous Molybdenum Disulfide Nanosheets. *J. Am. Chem. Soc*. **2016**, 138, 7965-7972.
2. Kresse, G.; Furthmüller, J. Efficient Iterative Schemes for *Ab initio* Total-Energy Calculations Using a Plane-Wave Basis Set. *Phys. Rev.* *B* **1996**, *54*, 11169-11186.
3. Kresse, G.; Furthmüller, J. Efficiency of *Ab initio* Total-Energy Calculations for Metals and Semiconductors Using a Plane-Wave Basis Set. *Comput. Mater. Sci.* **1996**, *6*, 15-50.
4. Blöchl, P. E. Projector augmented-wave method. *Phys. Rev. B* **1994**, *50*, 17953-17979.
5. Grimme, S.; Antony, J.; Ehrlich S.; Krieg, H. A Consistent and Accurate Ab Initio Parametrization of Density Functional Dispersion Correction (DFT-D) for the 94 Elements H-Pu *J. Chem. Phys*., **2010**, *132*, 154104.
6. Dudarev, S. L.; Botton, G. A.; Savrasov, S. Y.; Humphreys, C. J.; Sutton, A. P. Electron-Energy-Loss Spectra and the Structural Stability of Nickel Oxide: An LSDA+U Study. Phys. Rev. B **1998**, ***57***, 1505-1509.
7. Esters, M. R; Hennig, G.; Johnson, D. C. Dynamic Instabilities in Strongly Correlated VSe_2_ Monolayers and Bilayers. *Phys. Rev. B* **2017**, *96*, 235147.
8. Mou, J.; Gao, Y.; Wang, J.; Ma, J.; Ren, H. Hydrogen Evolution Reaction Activity Related to the Facet-Dependent Electrocatalytic Performance of NiCoP from First Principles*. RSC Advances* **2019**, *9*, 11755-11761.
9. Cramer C. J. *Essentials of Computational Chemistry: Theories and Models, 2^nd^ Edition*; John Wiley & Sons Ltd.: West Sussex, **2004**; Chapter 10.
10. Fujita, T.; Ito, Y.; Tan, Y.; Yamaguchi, H.; Hojo, D.; Hirata, A.; Voiry, D.; Chhowalla, M.; Chen, M. Chemically Exfoliated ReS_2_ Nanosheets. *Nanoscale* **2014**, *6*, 12458-12462.
11. Huang, J.; Gaod, H.; Xia, Y.; Sun, Y.; Xiong, J.; Li, Y.; Cong, S.; Guo, J.; Du, S.; Zou, G. Enhanced Photoelectrochemical Performance of Defect-Rich ReS_2_ Nanosheets in Visible-Light Assisted Hydrogen Generation. *Nano Energy* **2018**, *46*, 305-313.
12. Xu, X.; Zhao, H.; Wang, R.; Zhang, Z.; Dong, X.; Pan, J.; Hu, J.; Zeng, H. Identification of Few-Layer ReS_2_ as Photo-Electro Integrated Catalyst for Hydrogen Evolution. *Nano Energy* **2018**, *48*, 337-344.
13. Zhou, Y.; Song, E.; Zhou, J.; Lin, J.; Ma, R.; Wang, Y.; Qiu, W.; Shen, R.; Suenaga, K.; Liu, Q.; Wang, J.; Liu, Z.; Liu, J. Auto-Optimizing Hydrogen Evolution Catalytic Activity of ReS_2_ through Intrinsic Charge Engineering. *ACS Nano* **2018**, *12*, 4486-4493.
14. Zhou, G.; Guo, Z.; Shan, Y.; Wu, S.; Zhang, J.; Yan, K.; Liu, L.; Chu, P. K.; Wu, X. High-Efficiency Hydrogen Evolution from Seawater Using Hetero-Structured T/Td Phase ReS_2_ Nanosheets with Cationic Vacancies. *Nano Energy* **2019**, *55*, 42-48.
15. Han, X.; Li, N.; Kang, Y. B.; Dou, Q.; Xiong, P.; Liu, Q.; Lee, J. Y.; Dai, L.; Park, H. S. Unveiling Trifunctional Active Sites of a Heteronanosheet Electrocatalyst for Integrated Cascade Battery/Electrolyzer Systems. *ACS Energy Lett.* **2021**, *6*, 2460−2468.
16. Adofo, L. A; Kim, S. J.; Kim, H. -J.; Choi, S. H.; Lee, S. J.; Won, Y. S.; Balakrishan Kirubasankar, B.; Kim, J. W.; Oh, C. S. Ben-Smith, A.; Elorm, A. E.; Jeong, H. Y.; Lee, Y. H.; Kim, Y. -M.; Han, Y. -K.; Kim, S. M.; Kim, K. K. Universal Platform for Robust Dual-Atom Doped 2D Catalysts with Superior Hydrogen Evolution in Wide pH Media. *Small* **2024**, *20*, 2308672.
17. Lei, Y.; Pakhira, S.; Fujisawa, K.; Wang, X.; Iyiola, O. O.; López, N. P.; Elías, A. L.; Rajukumar, L. P.; Zhou, C.; Kabius, B.; Alem, N.; Endo, M.; Lv, R.; Mendoza-Cortes, J. L.; Terrones, M. Low-temperature Synthesis of Heterostructures of Transition Metal Dichalcogenide Alloys (W_x_Mo_1-x_S_2_) and Graphene with Superior Catalytic Performance for Hydrogen Evolution. *ACS Nano* **2017**, *11*, 5103−5112.
18. Zhuang, M.; Gan, L. Y.; Zou, M.; Dou, Y.; Ou, X.; Liu, Z.; Ding, Y.; Abidi, I. H.; Tyagi, A.; Jalali, M.; You, J.; Cao, A.; Luo, Z. Engineering Sub-100 nm Mo_(1-x)_W_x_Se_2_ Crystals for Efficient Hydrogen Evolution Catalysis. *J. Mater. Chem. A* **2018**, *6*, 2900−2907.
19. Lai, Z.; Chaturvedi, A.; Wang, Y.; Tran, T. H.; Liu, X.; Tan, C.; Luo, Z.; Chen, B.; Huang, Y.; Nam, G. H.; Zhang, Z.; Chen, Y.; Hu, Z.; Li, B.; Xi, S.; Zhang, Q.; Zong, Y.; Gu, L.; Kloc, C.; Du, Y.; et al. Preparation of 1T′-Phase ReS_2_*_x_*Se_2(1‑_*_x_*_)_ (*x* = 0−1) Nanodots for Highly Efficient Electrocatalytic Hydrogen Evolution Reaction. *J. Am. Chem. Soc.* **2018**, *140*, 8563-8568.
20. Yang, S. -Z.; Gong, Y.; Manchanda, P.; Zhang, Y. Y.; Ye, G.; Chen, S.; Song, L.; Pantelides, S. T.; Ajayan, P. M.; Chisholm, M. F.; Zhou, W. Rhenium-Doped and Stabilized MoS_2_ Atomic Layers with Basal-Plane Catalytic Activity. *Adv. Mater.* **2018**, *30*, 1803477.
21. Hu, P.; Long, G.; Chaturvedi, A.; Wang, S.; Tan, K.; He, Y.; Zheng, L.; Liu, G.; Ke, Y.; Zhou, Y.; Jiang, H.; Liu, Z.; Zhang, M.; Long, Y. Agent-Assisted VSSe Ternary Alloy Single Crystals As an Efficient Stable Electrocatalyst For the Hydrogen Evolution Reaction. *J. Mater. Chem.* *A* **2019**, *7*, 15714–15721.
22. Kwak, I. H.; Debela, T. T.; Kwon, I. S.; Seo, J.; Yoo, S. J.; Kim, J. -G.; Ahn, J. -P.; Park, J.; Kang, H. S. Anisotropic Alloying of Re_1-x_Mo_x_S_2_ Nanosheets to Boost the Electrochemical Hydrogen Evolution Reaction. *J. Mater. Chem. A* **2020**, *8*, 25131-25141.
23. Kwak, I. H.; Kwon, I. S.; Debela, T. T.; Abbas, H. G; Park, Y. C.; Seo, J.; Ahn, J. -P.; Lee, J. H.; Park, J.; Kang, H. S. Phase Evolution of Re_1-x_Mo_x_Se_2_ Alloy Nanosheets and Their Enhanced Catalytic Activity toward Hydrogen Evolution Reaction. *ACS Nano* **2020**, *14*, 11995-12005.
24. Kwon, I. S.; Kwak, I. H.; Debela, T. T.; Kim, J. Y.; Yoo, S. J.; Kim, J. -G.; Park, J.; Kang, H. S. Phase-Transition Mo_1-_*_x_*V*_x_*Se_2_ Alloy Nanosheets with Rich V-Se Vacancies and Their Enhanced Catalytic Performance of Hydrogen Evolution Reaction. *ACS Nano* **2021**, *15*, 14672-14682.
25. Kwon, I. S.; Kwak, I. H.; Zewdie, G. M.; Lee, S. J.; Kim, J. Y.; Yoo, S. J.; Kim, J. -G.; Park, J.; Kang, H. S. WSe_2_-VSe_2_ Alloyed Nanosheets to Enhance the Catalytic Performance of Hydrogen Evolution Reaction. *ACS Nano* **2022***, 16,* 12569-12579.
26. Guo, X.; Wu, T.; Zhao, S.; Fang, Y.; Xu, S.; Xie, M.; Huang, F. Band Structure Engineering of W Replacement in ReSe_2_ Nanosheets for Enhancing Hydrogen Evolution. *Chem. Commun.***2022**, *58*, 2682–2685.
27. Nguyen, H. T. T.; Adofo, L. A.; Yang, S. H.; Kim, H. J.; Choi, S. H.; Kirubasankar, B.; Cho, B. W.; Ben-Smith, A.; Kang, J.; Kim, Y. M.; Kim, S. M.; Han, Y. K.; Kim, K. K. 1T’ Re_x_Mo_1-x_S_2_-2H MoS_2_ Lateral Heterojunction for Enhanced Hydrogen Evolution Reaction Performance. *Adv. Funct. Mater.* **2023**, *33*, 2209572.
28. Lee, H. J.; Choe, M.; Yang, W.; Lee, S. W.; Park, Y. J.; Hwang, H.; Chhowalla, Lee, Z.; Shin, H. S. Phase-Engineered WS_2_ Monolayer Quantum Dots by Rhenium Doping*. ACS Nano* **2023,** *17*, 25731-25738.
29. Kwak, I. H.; Kim, J. Y.; Zewdie, G. M.; Yang, J. H.; Lee, K. S.; Yoo, S. J.; Kwon, I. S.; Park, J.; Kang, H. S. Electrocatalytic Activation in ReSe_2_-VSe_2_ Alloy Nanosheets to Boost Water-Splitting Hydrogen Evolution Reaction. *Adv. Mater* **2024**, *35*, 2310769.
30. Ihsan, J.; Kwak, I. H.; Kim, J. Y.; Zewdie, G. M.; Choi, J. H.; Lee, S. -G.; Lee, K. -G.; Kwon, I. S.; Park, J.; Kang, H. S. Alloying of ReS_2_ and VS_2_ Nanosheets Enhances Electrocatalytic Hydrogen Evolution Reaction *Adv. Funct. Mater.* **2024**, *34*, 2406755.
